# Supplementary material for: Optimal iron content in ready-to-use therapeutic foods for the treatment of severe acute malnutrition in the community settings: a protocol for the systematic review and meta-analysis
Source: BMJ Open. 2022 Mar 9;12(3):e057389. doi: 10.1136/bmjopen-2021-057389 (PMC8915355; doi:10.1136/bmjopen-2021-057389)
Supplement: Supplementary data [file bmjopen-2021-057389supp001.pdf]

Appendix 1: Search strategies for different electronic databases

PubMed

("Iron"[MeSH Terms] OR "iron, dietary"[MeSH Terms] OR "Iron"[Title/Abstract] OR "56Fe"[Title/Abstract] OR "Fe"[Title/Abstract] OR "ferro"[Title/Abstract] OR "ferrum"[Title/Abstract]) AND ("food, formulated"[MeSH Terms] OR "food, fortified"[MeSH Terms] OR "RUTF"[Title/Abstract] OR "RUTFs"[Title/Abstract] OR "ready to use therapeutic food*"[Title/Abstract] OR "ready to use therapeutic feed*"[Title/Abstract] OR "therapeutic food*"[Title/Abstract] OR "therapeutic diet*"[Title/Abstract] OR "enriched food*"[Title/Abstract] OR "enriched diet*"[Title/Abstract] OR "fortified food*"[Title/Abstract] OR "fortified diet*"[Title/Abstract] OR "supplemented food*"[Title/Abstract] OR "supplemental food*"[Title/Abstract] OR "supplementary food*"[Title/Abstract] OR "supplement food*"[Title/Abstract] OR "supplemented diet*"[Title/Abstract] OR "supplemental diet*"[Title/Abstract] OR "supplementary diet*"[Title/Abstract] OR "supplement diet*"[Title/Abstract] OR "RUSF"[Title/Abstract] OR "RUSFs"[Title/Abstract] OR "ready to use supplemental food*"[Title/Abstract] OR "ready to use supplementary food*"[Title/Abstract] OR "lipid based nutrient supplement*"[Title/Abstract] OR "LNS"[Title/Abstract]) AND ("Severe Acute Malnutrition"[MeSH Terms] OR "Malnutrition"[MeSH Terms] OR "Cachexia"[MeSH Terms] OR "Wasting Syndrome"[MeSH Terms] OR "Severe Acute Malnutrition"[Title/Abstract] OR "SAM"[Title/Abstract] OR "Malnutrition"[Title/Abstract] OR "malnourish*"[Title/Abstract] OR "Cachexia"[Title/Abstract] OR "nutritional deficien*"[Title/Abstract] OR "undernutrition"[Title/Abstract] OR "deficient nutrition"[Title/Abstract] OR "undernourish*"[Title/Abstract] OR "wasting"[Title/Abstract] OR "wasted"[Title/Abstract]) AND ("Infant"[MeSH Terms] OR "Child"[MeSH Terms] OR "infant*"[Title/Abstract] OR "infancy"[Title/Abstract] OR "baby"[Title/Abstract] OR "babies"[Title/Abstract] OR "newborn*"[Title/Abstract] OR "neonat*"[Title/Abstract] OR "neo nat*"[Title/Abstract] OR "child*"[Title/Abstract] OR "toddler*"[Title/Abstract] OR "youth"[Title/Abstract] OR "juvenile*"[Title/Abstract] OR "girl*"[Title/Abstract] OR "boy"[Title/Abstract] OR "boys"[Title/Abstract] OR "preschool*"[Title/Abstract] OR "pre school*"[Title/Abstract])

CINAHL

S1 (MH "Infant+")
S2 (MH "Child+")
S3 TI (infant* OR infancy OR baby OR babies OR newborn* OR neonat* OR "neo nat*" OR child* OR toddler* OR youth OR juvenile* OR girl* OR boy OR boys OR preschool* OR "preschool*")
S4 AB (infant* OR infancy OR baby OR babies OR newborn* OR neonat* OR "neo nat*" OR child* OR toddler* OR youth OR juvenile* OR girl* OR boy OR boys OR preschool* OR "preschool*")
S5 S1 OR S2 OR S3 OR S4
S6 (MH "Iron")
S7 TI (iron OR 56Fe OR Fe Or ferro OR ferrum)
S8 AB (iron OR 56Fe OR Fe Or Ferro OR ferrum)
S9 S6 OR S7 OR S8
S10 (MH "Food, Formulated")
S11 (MH "Food, Fortified")
S12 TI (RUTF OR RUTFs OR "ready to use therapeutic food*" OR "ready to use therapeutic feed*" OR RUSF OR RUSFs OR "ready to use supplemental food*" OR "ready to use supplementary food*" OR "lipid-based nutrient supplement*" OR LNS)
S13 AB (RUTF OR RUTFs OR "ready to use therapeutic food*" OR "ready to use therapeutic feed*" OR RUSF OR RUSFs OR "ready to use supplemental food*" OR "ready to use supplementary food*" OR "lipid-based nutrient supplement*" OR LNS)
S14 TI((enriched OR fortified OR supplement* OR therapeutic) W1 (food* OR diet*))
S15 AB((enriched OR fortified OR supplement* OR therapeutic) W1 (food* OR diet*))
S16 S10 OR S11 OR S12 OR S13 OR S14 OR S15
S17 (MH "Cachexia")
S18 (MH "Malnutrition")
S19 (MH "Wasting Syndrome")

S20 TI ("Severe Acute Malnutrition" OR SAM OR Malnutrition OR malnourish* OR Cachexia OR undernutrition OR undernourish* OR wasting OR wasted)
S21 AB ("Severe Acute Malnutrition" OR SAM OR Malnutrition OR malnourish* OR Cachexia OR undernutrition OR undernourish* OR wasting OR wasted)
S22 TI (nutrition* N1 deficien*)
S23 AB (nutrition* N1 deficien*)
S24 S17 OR S18 OR S19 OR S20 OR S21 OR S22 OR S23
S25 S5 AND S9 AND S16 AND S24
S26 S25 Exclude MEDLINE records

Embase

#1 'iron'/exp OR 'iron intake'/exp
#2 iron:ti,ab OR 56fe:ti,ab OR fe:ti,ab OR ferro:ti,ab OR ferrum:ti,ab
#3 #1 OR #2
#4 'ready to use therapeutic food'/exp OR 'fortified food'/exp OR 'ready to use supplementary food'/exp OR 'lipid based nutrient supplement'/exp OR 'dietary supplement'/exp
#5 ((enriched OR fortified OR supplement* OR therapeutic) NEXT/1 (food* OR diet*)):ti,ab
#6 rutf:ti,ab OR rutfs:ti,ab OR 'ready to use therapeutic food*':ti,ab OR 'ready to use therapeutic feed*':ti,ab OR rusf:ti,ab OR rusfs:ti,ab OR 'ready to use supplemental food*':ti,ab OR 'ready to use supplementary food*':ti,ab OR 'lipid based nutrient supplement*':ti,ab OR lns:ti,ab
#7 #4 OR #5 OR #6
#8 'malnutrition'/exp OR 'wasting syndrome'/exp OR 'cachexia'/exp
#9 (nutrition* NEAR/1 deficien*):ti,ab
#10 'severe acute malnutrition':ti,ab OR sam:ti,ab OR malnutrition:ti,ab OR malnourish*:ti,ab OR cachexia:ti,ab OR undernutrition:ti,ab OR undernourish*:ti,ab OR wasting:ti,ab OR wasted:ti,ab
#11 #8 OR #9 OR #10
#12 'infant'/exp OR 'infancy'/exp OR 'baby'/exp OR 'newborn'/exp OR 'toddler'/exp OR 'child'/exp OR 'preschool child'/exp OR 'juvenile'/exp OR 'girl'/exp OR 'boy'/exp
#13 infant*:ti,ab OR infancy:ti,ab OR baby:ti,ab OR babies:ti,ab OR newborn*:ti,ab OR neonat*:ti,ab OR 'neo nat*':ti,ab OR child*:ti,ab OR toddler*:ti,ab OR youth:ti,ab OR juvenile*:ti,ab OR girl*:ti,ab OR boy:ti,ab OR boys:ti,ab OR preschool*:ti,ab OR 'pre school*':ti,ab
#14 #12 OR #13
#15 #3 AND #7 AND #11 AND #14
#16 #15 NOT [medline]/lim

SCOPUS

(TITLE-ABS (iron OR 56fe OR fe OR Ferro OR ferrum)) AND ((TITLE-ABS (enriched OR fortified OR supplement* OR therapeutic) PRE/1 TITLE-ABS (food* OR diet*)) OR (TITLE-ABS (rutf OR rutfs OR "ready to use therapeutic food*" OR "ready to use therapeutic feed*" OR rusf OR rusfs OR "ready to use supplemental food*" OR "ready to use supplementary food*" OR "lipid-based nutrient supplement*" OR lns))) AND ((TITLE-ABS (nutrition* W/1 deficien*)) OR (TITLE-ABS ("Severe Acute Malnutrition" OR sam OR malnutrition OR malnourish* OR cachexia OR undernutrition OR undernourish* OR wasting OR wasted))) AND (TITLE-ABS (infant* OR infancy OR baby OR babies OR newborn* OR neonat* OR neo-nat* OR child* OR toddler* OR youth OR juvenile* OR girl* OR boy OR boys OR preschool* OR pre-school*)) AND NOT INDEX (Medline)

CENTRAL

#1 MeSH descriptor: [Iron] in all MeSH products
#2 MeSH descriptor: [Iron, Dietary] explode all trees
#3 Iron:ti,ab OR 56Fe:ti,ab OR Fe:ti,ab OR ferro:ti,ab OR ferrum:ti,ab
#4 #1 OR #2 OR #3
#5 MeSH descriptor: [Food, Formulated] explode all trees
#6 MeSH descriptor: [Food, Fortified] explode all trees
#7 ((enriched OR fortified OR supplement* OR therapeutic) NEXT (food* OR diet*)):ti,ab

#8 (ready NEXT to NEXT use NEXT therapeutic NEXT food*):ti,ab
#9 (ready NEXT to NEXT use NEXT therapeutic NEXT feed*):ti,ab
#10 (ready NEXT to NEXT use NEXT supplemental NEXT food*):ti,ab
#11 (ready NEXT to NEXT use NEXT supplementary NEXT food*):ti,ab
#12 (lipid NEXT based NEXT nutrient NEXT supplement*):ti,ab
#13 rutf:ti,ab OR rutfs:ti,ab OR rusf:ti,ab OR rusfs:ti,ab OR lns:ti,ab
#14 #5 OR #6 OR #7 OR #8 OR #9 OR #10 OR #11 OR #12 OR #13
#15 MeSH descriptor: [Severe Acute Malnutrition] explode all trees
#16 MeSH descriptor: [Malnutrition] explode all trees
#17 MeSH descriptor: [Cachexia] explode all trees
#18 MeSH descriptor: [Wasting Syndrome] explode all trees
#19 (nutrition* NEAR/1 deficien*):ti,ab
#20 (severe NEXT acute NEXT malnutrition):ti,ab
#21 sam:ti,ab OR malnutrition:ti,ab OR malnourish*:ti,ab OR cachexia:ti,ab OR undernutrition:ti,ab OR undernourish*:ti,ab OR wasting:ti,ab OR wasted:ti,ab
#22 #15 OR #16 OR #17 OR #18 OR #19 OR #20 OR #21
#23 MeSH descriptor: [Infant] explode all trees
#24 MeSH descriptor: [Child] explode all trees
#25 infant*:ti,ab OR infancy:ti,ab OR baby:ti,ab OR babies:ti,ab OR newborn*:ti,ab OR neonat*:ti,ab OR neo NEXT nat*':ti,ab OR child*:ti,ab OR toddler*:ti,ab OR youth:ti,ab OR juvenile*:ti,ab OR girl*:ti,ab OR boy:ti,ab OR boys:ti,ab OR preschool*:ti,ab OR pre NEXT school*:ti,ab
#26 #23 OR #24 OR #25
#27 #4 AND #14 AND #22 AND #26
#28 "accession number" near pubmed
#29 #27 NOT #28

Web of Science

#1 TI=(iron OR 56fe OR fe OR ferro OR ferrum)
#2 AB=(iron OR 56fe OR fe OR ferro OR ferrum)
#3 #1 OR #2
#4 TI=(RUTF OR RUTFs OR "ready to use therapeutic food*" OR "ready to use therapeutic feed*" OR "therapeutic food*" OR "therapeutic diet*" OR "enriched food*" OR "enriched diet*" OR "fortified food*" OR "fortified diet*" OR "supplemented food*" OR "supplemental food*" OR "supplementary food*" OR "supplement food*" OR "supplemented diet*" OR "supplemental diet*" OR "supplementary diet*" OR "supplement diet*" OR RUSF OR RUSFs OR "ready to use supplemental food*" OR "ready to use supplementary food*" OR "lipid based nutrient supplement*" OR LNS)
#5 AB=(RUTF OR RUTFs OR "ready to use therapeutic food*" OR "ready to use therapeutic feed*" OR "therapeutic food*" OR "therapeutic diet*" OR "enriched food*" OR "enriched diet*" OR "fortified food*" OR "fortified diet*" OR "supplemented food*" OR "supplemental food*" OR "supplementary food*" OR "supplement food*" OR "supplemented diet*" OR "supplemental diet*" OR "supplementary diet*" OR "supplement diet*" OR RUSF OR RUSFs OR "ready to use supplemental food*" OR "ready to use supplementary food*" OR "lipid based nutrient supplement*" OR LNS)
#6 #4 OR #5
#7 TI=(nutrition* NEAR/1 deficien*)
#8 AB=(nutrition* NEAR/1 deficien*)
#9 TI=("Severe Acute Malnutrition" OR sam OR malnutrition OR malnourish* OR cachexia OR undernutrition OR undernourish* OR wasting OR wasted)
#10 AB=("Severe Acute Malnutrition" OR sam OR malnutrition OR malnourish* OR cachexia OR undernutrition OR undernourish* OR wasting OR wasted)
#11 #7 OR #8 OR #9 OR #10
#12 TI=(infant* OR infancy OR baby OR babies OR newborn* OR neonat* OR "neo nat*" OR child* OR toddler* OR youth OR juvenile* OR girl* OR boy OR boys OR preschool* OR "pre school*")

#13 **AB=(infant* OR infancy OR baby OR babies OR newborn* OR neonat* OR "neo nat*" OR child* OR toddler* OR youth OR juvenile* OR girl* OR boy OR boys OR preschool* OR "pre school*")**
#14 **#12 OR #13**
#15 **#3 AND #6 AND #11 AND #14**

LILACS

(mh:("Iron")) OR ((mh:("iron, dietary"))) OR ((ti:(iron OR 56fe OR fe OR ferro OR ferrum))) OR ((ab:(iron OR 56fe OR fe OR ferro OR ferrum))) AND ((mh:("food, formulated")) OR ((mh:("food, fortified"))) OR ((ti:(rutf OR rutfs OR "ready to use therapeutic food*" OR "ready to use therapeutic feed*" OR "therapeutic food*" OR "therapeutic diet*" OR "enriched food*" OR "enriched diet*" OR "fortified food*" OR "fortified diet*" OR "supplemented food*" OR "supplemental food*" OR "supplementary food*" OR "supplement food*" OR "supplemented diet*" OR "supplemental diet*" OR "supplementary diet*" OR "supplement diet*" OR rusf OR rusfs OR "ready to use supplemental food*" OR "ready to use supplementary food*" OR "lipid based nutrient supplement*" OR lns))) OR ((ab:(rutf OR rutfs OR "ready to use therapeutic food*" OR "ready to use therapeutic feed*" OR "therapeutic food*" OR "therapeutic diet*" OR "enriched food*" OR "enriched diet*" OR "fortified food*" OR "fortified diet*" OR "supplemented food*" OR "supplemental food*" OR "supplementary food*" OR "supplement food*" OR "supplemented diet*" OR "supplemental diet*" OR "supplementary diet*" OR "supplement diet*" OR rusf OR rusfs OR "ready to use supplemental food*" OR "ready to use supplementary food*" OR "lipid based nutrient supplement*" OR lns)))) AND ((mh:("Severe Acute Malnutrition")) OR ((mh:("Malnutrition"))) OR ((mh:("cachexia"))) OR ((mh:("wasting syndrome"))) OR ((ti:("Severe Acute Malnutrition" OR sam OR malnutrition OR malnourish* OR cachexia OR "nutritional deficien*" OR undernutrition OR "deficient nutrition" OR undernourish* OR wasting OR wasted))) OR ((ab:("Severe Acute Malnutrition" OR sam OR malnutrition OR malnourish* OR cachexia OR "nutritional deficien*" OR undernutrition OR "deficient nutrition" OR undernourish* OR wasting OR wasted)))) AND ((mh:(infant)) OR ((mh:(child))) OR ((ti:(infant* OR infancy OR baby OR babies OR newborn* OR neonat* OR "neo nat*" OR child* OR toddler* OR youth OR juvenile* OR girl* OR boy OR boys OR preschool* OR "pre school*"))) OR ((ab:(infant* OR infancy OR baby OR babies OR newborn* OR neonat* OR "neo nat*" OR child* OR toddler* OR youth OR juvenile* OR girl* OR boy OR boys OR preschool* OR "pre school*")))) AND (db:("LILACS"))

Global Index Medicus

((mh:("Iron")) OR ((mh:("iron, dietary"))) OR ((ti:(iron OR 56fe OR fe OR ferro OR ferrum))) OR ((ab:(iron OR 56fe OR fe OR ferro OR ferrum))) AND ((mh:("food, formulated")) OR ((mh:("food, fortified"))) OR ((ti:(rutf OR rutfs OR "ready to use therapeutic food*" OR "ready to use therapeutic feed*" OR "therapeutic food*" OR "therapeutic diet*" OR "enriched food*" OR "enriched diet*" OR "fortified food*" OR "fortified diet*" OR "supplemented food*" OR "supplemental food*" OR "supplementary food*" OR "supplement food*" OR "supplemented diet*" OR "supplemental diet*" OR "supplementary diet*" OR "supplement diet*" OR rusf OR rusfs OR "ready to use supplemental food*" OR "ready to use supplementary food*" OR "lipid based nutrient supplement*" OR lns))) OR ((ab:(rutf OR rutfs OR "ready to use therapeutic food*" OR "ready to use therapeutic feed*" OR "therapeutic food*" OR "therapeutic diet*" OR "enriched food*" OR "enriched diet*" OR "fortified food*" OR "fortified diet*" OR "supplemented food*" OR "supplemental food*" OR "supplementary food*" OR "supplement food*" OR "supplemented diet*" OR "supplemental diet*" OR "supplementary diet*" OR "supplement diet*" OR rusf OR rusfs OR "ready to use supplemental food*" OR "ready to use supplementary food*" OR "lipid based nutrient supplement*" OR lns)))) AND ((mh:("Severe Acute Malnutrition")) OR ((mh:("Malnutrition"))) OR ((mh:("cachexia"))) OR ((mh:("wasting syndrome"))) OR ((ti:("Severe Acute Malnutrition" OR sam OR malnutrition OR malnourish* OR cachexia OR "nutritional deficien*" OR undernutrition OR "deficient nutrition" OR undernourish* OR wasting OR wasted))) OR ((ab:("Severe Acute Malnutrition" OR sam OR malnutrition OR malnourish* OR cachexia OR "nutritional deficien*" OR undernutrition OR "deficient nutrition" OR undernourish* OR wasting OR wasted)))) AND ((mh:(infant)) OR ((mh:(child))) OR ((ti:(infant* OR infancy OR baby OR babies OR newborn* OR neonat* OR "neo nat*" OR child* OR toddler* OR youth OR juvenile* OR girl* OR boy OR boys OR preschool* OR "pre school*"))) OR ((ab:(infant* OR infancy OR baby OR babies OR newborn* OR neonat* OR "neo nat*" OR child* OR toddler* OR youth OR juvenile* OR girl* OR boy OR boys OR preschool* OR "pre school*"))))) AND (collection_gim:("IMSEAR" OR "IMEMR" OR "WPRIM"))
