# Supplementary material for: Optimal iron content in ready-to-use therapeutic foods for the treatment of severe acute malnutrition in the community settings: a protocol for the systematic review and meta-analysis
Source: BMJ Open. 2022 Mar 9;12(3):e057389. doi: 10.1136/bmjopen-2021-057389 (PMC8915355; doi:10.1136/bmjopen-2021-057389)
Supplement: Supplementary data [file bmjopen-2021-057389supp002.pdf]

Author	Publication year	Name of Data Extractor	Form: conference abstract only (1), journal publication (2), multiple publications of the same trial (3)	Study Design	Study Setting		Population
					Country	Year(s) of Data Collection	Description of Participants<br>Inclusion Criteria

Exclusion Criteria	% Female (N) (Give for total study population)

	Macronutrient composition of RUTF in the intervention group		Iron dos/100g of RUTF in the experimental group	Zinc dos/100 mg of RUTF in the experimental group	Frequency of RUTF (packets per day)
Total number of eligible participants in all the study groups					

Duration of intervention RUTF (How many days of intervention was given)	Characteristic of comparison		Study confounders		What outcome did authors report	Notes
	Standard RTUFT? (Yes or No)	**Iron content in Standard RUTF (Normal dose is 10-14 mg/100 g)**	Were results adjusted for confounders? (Y/N) (Fill only for observational studies)	What confounders were considered?		

Study (name, year)	**Hemoglobin at longest follow up**		
	No of events in interventional RUTF	Total number in interventional RUTF	number of events in control RUTF group

	**Summary estimate of intervention RUTF vs. Standard RUTF (OR, RR, HR)**		Intervention
number of participants in control RUTF group	notes	notes	Mean

RUTF		Control RUTF			Intervention in RUTF	
SD	Number in R	Mean	SD	Number in t	median	IQR

	Control RUTF		
Number in g	Median	IQR	Number in g

Summary estimate (mean difference or Standardized mean difference)

ɟroup

Notes

	A	B	C	D	E	F	G	H	I	J	K	L	M	N	O	P
1		**Anemia at longest follow up**				**Summary estimate of intervention RUTF vs. Standard RUTF (OR, RR, HR)**		Intervention RUTF			Control RUTF			Intervention in RUTF		
2	Study (name, year)	No of events in interventional RUTF	RUTF	control RUTF group	RUTF group	notes	notes	Mean	SD	RUTF	Mean	SD	control RUTF	median	IQR	group

	Q	R	S	T
1	Control RUTF			Summary estimate (mean difference or Standardized mean difference)
2	Median	IQR	group	

Study (name, year)	Iron deficiancy anemia at longest follow up		
	No of events in interventional RUTF	Total number in interventional RUTF	number of events in control RUTF group

	**Summary estimate of intervention RUTF vs. Standard RUTF (OR, RR, HR)**		Intervention RUTF			Control RUT
number of participants in control RUTF group	notes	notes	Mean	SD	Number in RUTF interventional group	Mean

TF		Intervention in RUTF			Control RUTF		
SD	Number in the control RUTF group	median	IQR	Number in group	Median	IQR	Number in group

Summary estimate (mean difference or Standardized mean difference)

Study (name, year)	**SAM Recovery at longest follow up**				**Summary estimate of intervention RUTF vs. Standard RUTF (OR, RR, HR)**	
	No of events in interventional RUTF	Total number in interventional RUTF	number of events in control RUTF group	number of participants in control RUTF group	notes	notes

Study (name, year)	**Adverse effects at longest follow up**				**Summary estimate of intervention RUTF vs. Standard RUTF (OR, RR, HR)**
	No of events in interventional RUTF	Total number in interventional RUTF	number of events in control RUTF group	number of participants in control RUTF group	notes

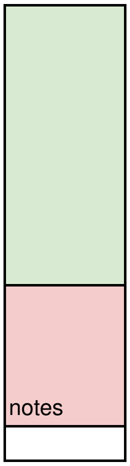
notes

Study (name, year)	Adverse effects at longest follow up				Summary estimate of intervention RUTF vs. Standard RUTF (OR, RR, HR)	
	No of events in interventional RUTF	Total number in interventional RUTF	number of events in control RUTF group	number of participants in control RUTF group	notes	notes

	A	B	C	D	E	F	G	H	I	J	K	L	M	N
1	Study Name	Intervention RUTF			Control RUTF			Intervention in RUTF			Control RUTF			Summary esti
2		Mean	SD	Number in RUTF inter	Mean	SD	Number in the	median	IQR	Number in gro	Median	IQR	Number in group	
3														
4														

Study (name, year)	**Adverse effects at longest follow up**				**Summary estimate of intervention RUTF vs. Standard RUTF (OR, RR, HR)**	
	No of events in interventional RUTF	Total number in interventional RUTF	number of events in control RUTF group	number of participants in control RUTF group	notes	notes

	A	B	C	D	E	F	G	H	I	J	K	L	M	N	O	P	Q	R	S	T
1	Study ID (Last name and yea		Z scores						Kg						Change in weight (Kg) or Z scores					
2			Intervention			Control			Intervention			Control			Intervention			Control		
3			Mean	SD	Number in gro	Mean	SD	Number in gro	Mean	SD	Number in gro	Mean	SD	Number in gro	Mean	SD	Number in gro	Mean	SD	Number in gro
4																				
5																				

BMJ Publishing Group Limited (BMJ) disclaims all liability and responsibility arising from any reliance placed on this supplemental material which has been supplied by the author(s)

	U
1	Comments
2	
3	up
4	
5	

	A	B	C	D	E	F	G	H	I	J	K	L	M	N	O	P	Q	R	S	T
1	Study ID (Last	name and yea	Z scores						Kg						Change in weight (Kg) or Z scores					
2			Intervention			Control			Intervention			Control			Intervention			Control		
3			Mean	SD	Number in gro	Mean	SD	Number in gro	Mean	SD	Number in gro	Mean	SD	Number in gro	Mean	SD	Number in gro	Mean	SD	Number in gro
4																				
5																				

	U
1	Comments
2	
3	up
4	
5	

	A	B	C	D	E	F	G	H	I	J	K	L	M	N	O	P	Q	R	S	T	U
1	Study ID (Last name and year)		Z scores						Centimeters						Change in height (cm) or Z scores						Comments
2			Intervention			Control			Intervention			Control			Intervention			Control			
3			Mean	SD	Number in gro	Mean	SD	Number in gro	Mean	SD	Number in gro	Mean	SD	Number in gro	Mean	SD	Number in gro	Mean	SD	Number in group	
4																					
5																					

	A	B	C	D	E	F	G	H	I	J	K	L	M	N	O	P	Q	R	S	T
1	Study ID (Last name and yea		Z scores						Centimeters						Change in height (cm) or Z scores					
2			Intervention			Control			Intervention			Control			Intervention			Control		
3			Mean	SD	Number in gro	Mean	SD	Number in gro	Mean	SD	Number in gro	Mean	SD	Number in gro	Mean	SD	Number in gro	Mean	SD	Number in gro
4																				
5																				

	U
1	Comments
2	
3	up
4	
5	

	A	B	C	D	E	F	G	H	I	J	K	L	M	N	O	P	Q	R	S	T
1	Study ID (Last name and year)		Z scores						Centimeters						Change in height (cm) or Z scores					
2			Intervention			Control			Intervention			Control			Intervention			Control		
3			Mean	SD	Number in gro	Mean	SD	Number in gro	Mean	SD	Number in gro	Mean	SD	Number in gro	Mean	SD	Number in gro	Mean	SD	Number in gro
4																				
5																				

	U
1	Comments
2	
3	up
4	
5	

	A	B	C	D	E	F	G	H	I	J	K	L	M	N	O	P	Q	R	S	T
1	Study ID (Last name and year)		Z scores						BMI						Change in BMI or Z scores					
2			Intervention			Control			Intervention			Control			Intervention			Control		
3			Mean	SD	Number in gro	Mean	SD	Number in gro	Mean	SD	Number in gro	Mean	SD	Number in gro	Mean	SD	Number in gro	Mean	SD	Number in gro
4																				
5																				

	U
1	Comments
2	
3	up
4	
5	

Study ID (Last name and year	Z scores						BMI						Change in BMI or Z scores						Comments
	Intervention			Control			Intervention			Control			Intervention			Control			
	Mean	SD	Number in gro	Mean	SD	Number in gro	Mean	SD	Number in gro	Mean	SD	Number in gro	Mean	SD	Number in gro	Mean	SD	Number in group	

Study ID (Last name and year	Z scores						BMI						Change in BMI or Z scores						Comments
	Intervention			Control			Intervention			Control			Intervention			Control			
	Mean	SD	Number in gro	Mean	SD	Number in gro	Mean	SD	Number in gro	Mean	SD	Number in gro	Mean	SD	Number in gro	Mean	SD	Number in group	
