# Supplementary material for: Optimal iron content in ready-to-use therapeutic foods for the treatment of severe acute malnutrition in the community settings: a protocol for the systematic review and meta-analysis
Source: BMJ Open. 2022 Mar 9;12(3):e057389. doi: 10.1136/bmjopen-2021-057389 (PMC8915355; doi:10.1136/bmjopen-2021-057389)
Supplement: Author's manuscript [file bmjopen-2021-057389.draft_revisions.pdf]

BMJ Open is committed to open peer review. As part of this commitment we make the peer review history of every article we publish publicly available.

When an article is published we post the peer reviewers' comments and the authors' responses online. We also post the versions of the paper that were used during peer review. These are the versions that the peer review comments apply to.

The versions of the paper that follow are the versions that were submitted during the peer review process. They are not the versions of record or the final published versions. They should not be cited or distributed as the published version of this manuscript.

BMJ Open is an open access journal and the full, final, typeset and author-corrected version of record of the manuscript is available on our site with no access controls, subscription charges or pay-per-view fees (http://bmjopen.bmj.com).

If you have any questions on BMJ Open's open peer review process please email info.bmjopen@bmj.com

BMJ Open

Optimal iron content in ready-to-use therapeutic foods for the treatment of severe acute malnutrition in the community settings. A protocol for the systematic review and meta-analysis

Journal:	*BMJ Open*
Manuscript ID	bmjopen-2021-057389
Article Type:	Protocol
Date Submitted by the Author:	14-Sep-2021

Complete List of Authors:	Imdad, Aamer; The Aga Khan University<br>François, Melissa; The Aga Khan University<br>Chen, Fanny F; The Aga Khan University<br>Smith, Abigail; The Aga Khan University<br>Tsistinas, Olivia; The Aga Khan University<br>Tanner-Smith, Emily; The Aga Khan University<br>Das, Jai K.; The Aga Khan University<br>Bhutta, Zulfiqar; The Aga Khan University

Keywords:	PAEDIATRICS, NUTRITION & DIETETICS, PUBLIC HEALTH

SCHOLARONE™
Manuscripts

Optimal iron content in ready-to-use therapeutic foods for the treatment of severe acute malnutrition in the community settings. A protocol for the systematic review and meta-analysis

Aamer Imdad, Melissa François, Fanny F Chen, Abigail Smith, Olivia J Tsistinas, Emily Tanner-Smith, Jai Das, Zulfiqar Ahmed Bhutta

Authors' information

Contact Author

Aamer Imdad, MBBS, MPH

Assistant Professor of Pediatrics

Division of Pediatric Gastroenterology, Hepatology and Nutrition

SUNY Upstate Medical University, Syracuse, NY

725 Irving Street, Syracuse, NY 13210

Email: imdada@upstate.edu

Phone: 315-464-8444

Fax: 315-464-8443

Melissa François

SUNY Upstate Medical University

Syracuse, New York, USA

FrancoMe@upstate.edu

Fanny Chen

SUNY Upstate Medical University

Syracuse, New York, USA

ChenF@upstate.edu

Abigail Smith

SUNY Upstate Medical University

Syracuse, New York, USA

SmithAb@upstate.edu

Olivia Tsistinas

For peer review only

SUNY Upstate Medical University
Syracuse, New York, USA
TsistiJO@upstate.edu

Emily Tanner-Smith
University of Oregon
etanners@uoregon.edu
Eugene, Oregon, USA

Jai Das
Aga Khan University
Karachi, Sindh, Pakistan
Jai.das@aku.edu

For peer review only

Zulfiqar Ahmed Bhutta
Institute for Global Health & Development, The Aga Khan University
Karachi, Sindh, Pakistan
zulfiqar.bhutta@aku.edu

Word Count: 3471

For peer review only

ABSTRACT

Introduction

The current standard of care for children with severe acute malnutrition involves the use of ready-to-use therapeutic food (RUTF) to promote growth; however, the precise formulation to achieve optimal recovery remains unclear. Emerging research suggests that alternative RUTF formulations may be more effective in correcting SAM related complications such as anemia and iron deficiency. This systematic review commissioned by the WHO aims to synthesize the most recent research on iron content in RUTF and related products in the community based treatment of uncomplicated severe malnutrition in children aged 6 months and older.

Methods and Analysis

We will search multiple electronic databases. We will include randomized controls trials and non-randomized studies with a control arm. The intervention group will be infants who received RUTF treatments other than the current recommended guidelines set forth by the WHO. The comparison group is children receiving RUTF containing iron at the current WHO recommended level of 1.9 mg/100 kcal (10-14 mg/100 g). The primary outcomes of interest include blood hemoglobin concentration, any anemia, severe anemia, iron deficiency anemia, recovery from severe acute malnutrition and any adverse outcome. We will use meta-analysis to pool findings if sufficient homogeneity exists and data are available from at least two studies. Risk of bias in studies will be evaluated using the Cochrane risk of bias (ROB 2.0) and Cochrane ROBINS-I tools. We will use the Grading of Recommendations Assessment, Development, and Evaluation (GRADE) approach to examine the overall certainty of evidence.

Ethics and Dissemination: This is a systematic review and will not involve direct contact with human subjects. The findings of this review will be published in a peer-review journal and will guide the WHO's recommendation on optimal iron content in ready-to-use therapeutic foods for the treatment of severe acute malnutrition in children aged 6-59 months.

Key Words: Iron, ready-to-use therapeutic food, wasting, malnutrition, growth failure

For peer review only

ARTICLE SUMMARY: STRENGTHS AND LIMITATIONS OF THIS STUDY

- We will search several databases for relevant literature and will included randomised and non-randomised studies.
- We will assess risk-of-bias for each outcome and use the Grading of Recommendations Assessment, Development, and Evaluation to assess the overall quality of evidence.
- We will conduct subgroup analysis for age group (<24 months vs. 24-59 months vs. > 59 months), country, type of ready-to-use therapeutic food (RUTF), role of RUTF (RUTF as main treatment vs. RUTF as a supplementary intervention), type of participants (Studies that included children with HIV vs. Studies with children without HIV), hospitalization, iron compound and dose.
- We will conduct meta-analyses if data are available from more than one study, and if there is clinical and methodological homogeneity in the included studies.
- All the included studies may not report the data for all the outcomes mentioned in this review which will be a limitation of the study.

For peer review only

For peer review only

Full manuscript

BACKGROUND

The World Health Organization (WHO) estimates over 45 million children under the age of five worldwide suffered from wasting in 2020 alone[1]. Malnourished children have a 5-20 fold increased risk of mortality compared to well-nourished children and are at greater risk for immunodeficiency and neurodevelopmental delay[2]. Severe acute malnutrition involves deficits of several key macronutrients and micronutrients, with studies showing higher rates of anemia in children suffering from malnutrition [3-5]. The prevalence of anemia in severely acutely malnourished children ranges from 40% to 90%, and about half of the anemia is attributed to iron deficiency[6]. Iron deficiency in severely malnourished children could be due to low intake, increased losses, higher demand, and poor absorption. Iron is essential for adequate catch-up growth and neurological development in children with severe acute malnutrition[3,7,8].

The current standard of care for children with severe acute malnutrition involves the use of ready-to-use therapeutic food (RUTF) to promote growth[9]; however, the precise formulation to achieve optimal recovery remains unclear.[10] Moreover, recent studies suggest alternative

RUTF formulations may be more effective in correcting anemia and iron deficiency in children with severe acute malnutrition compared to current standard RUTF formulations [3,11,12]. In addition to the need for sufficient iron intake to treat iron deficiency, it remains unclear whether improving the iron status among the undernourished children increases the risk of complications, including the risk of morbidities such as diarrhea, malaria, and undesired changes in the microbiome[13-15]. This aspect is especially of concern in malaria-endemic regions in Africa because nearly a third (27%) of all children affected by wasting worldwide reside in Africa[2,15]. Due to the critical role of RUTF in the medical management of children with severe acute malnutrition, more information is needed to generate formulations with optimal iron levels for treatment of anemia and optimal growth and development in children suffering from malnutrition [7,8,11]. Therefore, this review aims to synthesize the most recent research on the iron content in RUTF and related products in the treatment of severe malnutrition in children 6-59 months of age.

OBJECTIVE

Primary Objective*:* In children aged 6 months or older with uncomplicated severe acute malnutrition being treated in the community settings, does increased iron dose in RUTF compared with the WHO recommendation for iron fortification of RUTF improve outcomes such as blood hemoglobin concentration, recovery from iron-deficiency anemia, etc.?

Secondary Objective*:* In children aged 6 months or older with uncomplicated severe acute malnutrition being treated in the community settings, what mechanisms other than increased dose of iron in RUTF (such as type, composition, zinc, phytate content etc.) can help increase the bioavailability of iron and improve the recovery from iron deficiency anemia?

METHODS AND ANALYSIS

We will follow the standard guidelines of the Cochrane Collaboration to conduct the systematic review. We will register a detailed protocol on PROSPERO and report the systematic review findings according to PRISMA 2020 guidelines[16].

For peer review only

Study type: We will consider randomized trials and that may include individual and cluster randomized trials. We will also consider non-randomized studies if there are not enough randomized studies. The data will be analyzed separately for randomized and non-randomized studies. We will include the following non-randomized studies: Non-randomized controlled clinical trials, and controlled before and after studies (CBAs). The definitions of eligible studies design and consideration for inclusion in this review are available in table 1. Case-control studies, interrupted time series, program evaluations, case reports, case series, and commentaries will be excluded.

Population

We will include studies that examined children age 6 months or older with severe acute malnutrition being managed in the outpatient settings. We will include studies that defined severe acute malnutrition based on weight for height z scores (< -3 SD for WHO growth standards) or mid-upper arm circumference (< 115 mm) or presence of bilateral edema[17]. We will exclude studies of children admitted to the hospital, as those participants might have complications such as pneumonia and severe diarrhea, and the optimal route of delivery of iron might be different for these children. However, we will consider studies that included children who were previously admitted to hospital and are now being rehabilitated in the community settings with the help of RUTF. We will consider studies that included children infected with human immunodeficiency virus (HIV). We will also include studies that included participants with Malaria. We will exclude studies done specifically on participants who have chronic diseases, genetic disorders, or congenital anomalies.

Intervention

We will include studies that assessed the use of RUTF for the treatment of severe acute malnutrition in community settings. We will include studies in which different formulations/doses of iron fortified in RUTF were studied for the treatment of severe acute malnutrition. We will include studies of iron-fortified RUTF and exclude those studies where iron was given separately as supplements, such as tablets, syrup, multiple micronutrient powder, etc. We will include studies where RUTF is given as the main intervention, and it met total daily requirements or was given as a supplementary intervention to the usual diet for children with severe acute malnutrition recovering at home. WHO recommends that a standard RUTF should

have at least 1.9 mg/100 kcal (or 10-14 mg/100 g) of iron to treat severe acute malnutrition in children 6-59 months of age. We will include studies irrespective of the type of RUTF used, i.e., standard RUTF vs. low milk-based vs. non-milk-based vs. locally prepared. We will exclude studies where RUTF was given to healthy children for the prevention of severe acute malnutrition or to those with moderate acute malnutrition. Studies that only include the use of traditional/family/home foods as part of complementary feeding will be excluded. We will also exclude studies that address the food used as part of complementary feedings.

Comparison

For the primary objective of the review, the comparison group will be children receiving RUTF containing iron at the current WHO recommended level of 1.9 mg/100 kcal (10-14 mg/100 g) to treat severe acute malnutrition. For the secondary objective of the review, we will consider studies where different types/doses/techniques of RUTF are studied to improve the bioavailability of iron even though the iron content may be the same in all kinds of RUTF given to children. We will exclude studies where the comparison group did not receive any RUTF.

OUTCOMES

Primary Outcomes

- Blood hemoglobin concentration (continuous outcome) at 1 month, 3 months, 6 months, and the longest follow-up.
- Any Anemia (dichotomous outcome, as defined by authors) at 1 month, 3 months, 6 months, and the longest follow-up.
- Severe anemia (dichotomous outcome, as defined by authors) at 1 month, 3 months, 6 months, and the longest follow-up.
- Iron deficiency anemia (dichotomous outcome as defined by authors) at 1 month, 3 months, 6 months, and the longest follow-up.
- Recovery from severe acute malnutrition (dichotomous outcome, as defined by authors) at 1 months, 3 months, 6 months, and the longest follow-up.
- Any Adverse events (Dichotomous outcome)

Secondary Outcomes

- Serum ferritin level (continuous outcome) at 1 month, 3 months, 6 months, and the longest follow-up.

For peer review only

- Serum zinc level (continuous outcome) at 1 month, 3 months, 6 months, and the longest follow-up.
- Serum copper level (continuous outcome) at 1 month, 3 months, 6 months, and the longest follow-up.
- Serum iron level (continuous outcome) at 1 month, 3 months, 6 months, and the longest follow-up.
- Adverse events Malaria: (as defined by authors) (dichotomous outcome) at 1 month, 3 months, 6 months, and the longest follow-up.
- Relapse (dichotomous outcome) at 1 months, 3 month, 6 months, and the longest follow-up.
- All-cause mortality (dichotomous outcome) at 1 month, 3 months, 6 months, and the longest follow-up.
- Admission to inpatient facility. (dichotomous outcome) at 1 month, 3 months, 6 months, and the longest follow-up.
- Withdrawal from trial (Dichotomous outcome)
- Constipation (<3 bowel movements per week) (dichotomous outcome) at 1 month, 3 months, 6 months, and the longest follow-up.
- Adverse events Diarrhea: (>3 loose stools per day) (dichotomous outcome) at 1 month, 3 months, 6 months, and the longest follow-up.
- Adverse events: Pneumonia (as defined by authors) (dichotomous outcome) at 1 month, 3 months, 6 months, and the longest follow-up.
- Weight for age (kg or Z scores) at 1 month, 3 months, 6 months, and the longest follow-up.
- Height for age (cm or Z scores) at 1 month, 3 months, 6 months, and the longest follow-up.
- Weight for height Z score at 1 month, 3 months, 6 months, and the longest follow-up.
- Microbiome outcomes: Alpha diversity and Beta Diversity
- Neurodevelopmental outcomes (continuous outcome) at 1 year and the longest follow-up.

  The term neurodevelopment is a composite term that refers to cognitive, neurological and/or sensory outcomes. This assessment may include intellectual disability as measured on the Mental Developmental Index of the Bayley Scales of Infant

For peer review only

Development, gross motor delay measured on Gross Motor Function Classification System, and so on.

If a study does not report outcomes at 1 month of follow up but instead reports outcomes between 2 to 7 weeks of follow up, it will be included with outcomes at 1 month. Similarly, if a study does not report outcomes at 3 months of follow up but instead reports outcomes between 2 and 4 months, it will be counted at 3 months. For 6 months follow up, it will be included with outcomes reported between 4 to 7 months of follow up.

LITERATURE SEARCH

We will conduct systematic electronic queries using key terms in several databases, including PubMed, EMBASE, the Cochrane Central Register for Controlled Trials, Web of Science, CINHAL,
Scopus, LILACS, and WHO Global Index Medicus. No search restrictions will be used to exclude studies based on the outcome, publication year, publication status, or language. The references of formerly published reviews and recently published studies will be examined for potential inclusion. We will also use the citation tracking function of the included studies in the PubMed to look for any eligible studies. In addition to the above resources, ClinicalTrials.gov will be used to identify studies currently underway. We will also use the clinical trial registration number to find all the relevant studies published from a particular trial. Finally, we will search the websites of pertinent international agencies such as WHO (including WHO's Reproductive Health Library, electronic Library of Evidence for Nutrition Actions and Global database on the Implementation of Nutrition Action), UNICEF, Global Alliance for Improved Nutrition, International Food Policy Research Institute, International Initiative for Impact Evaluation (3ie), Nutrition International, World Bank, USAID and affiliates (e.g., FANTA, SPRING), and the World Food Program. Proposed search strategies for different electronic databases are shown in Appendix 1 supplementary document.

DATA EXTRACTION AND SYNTHESIS

Selection of studies

Searches from all the databases will be combined in bibliographic software (EndNote), and duplicates will be removed. Two authors will use a three-phase approach in duplicate to screen

For peer review only

studies identified from the search for eligibility. In the first phase, authors will screen the titles and abstracts to identify potentially eligible ones; studies selected during this initial phase will then go on to a full-text review as the second phase. Lastly, studies determined to be eligible subsequent to full-text review will undergo data extraction during the third and final phase. The software Covidence14[18] will be used to assist the screening process. Two authors will independently extract data from screened studies and compare their findings. Potential conflicts will be resolved through discussion, and the senior author on the team may assist as needed to resolve any conflicts. If a study is only available in abstract form, we will write to authors to obtain details on methods and results. If we cannot get full details of the study methods available in the abstract, we will decide about inclusion based on details available in the abstract. If a study is available in a language other than English, we will attempt to complete the translation using local resources. If a study was published in more than one report (multiple publications), we will count those multiple reports as a single study and extract information from all the available reports as needed.

Data extraction

A data extraction form will be designed to support the review and data extraction process. Two authors will independently extract the data and compare their findings. Any conflict will be resolved by discussion and with the help of the senior author on the team if needed. The following information will be extracted for each study where available: study design (randomized control trial, quasi-randomized experimental design or cohort study, CBA), study site (country/region), study year, study type, intervention (dose, duration, frequency and composition), exposures, comparison, outcomes, whether the results were adjusted for confounders, and risk of bias. To avoid reviewer bias, we are deciding a priori the order of preference for extracting outcomes when data might be available in several formats. For randomized studies, we will prefer data that require the least manipulation by authors or inference by reviewers. We will extract the raw values (for example, means and standard deviations) rather than calculated effect sizes (e.g. Cohen's d). For mortality data, we will give preference to denominators in the following order: number with the definite outcome known (or imputed as described below), number randomised, and child-years. For morbidity outcomes to which both survivors and non-survivors may have contributed data (for example, the incidence of pneumonia), we will give preference to child years, number with the definite outcome known, and number randomized.

For peer review only - http://bmjopen.bmj.com/site/about/guidelines.xhtml

Studies with missing data

We will document attrition during data extraction. If the authors report the missing data and report results using imputations for the missing data, we will use the latter. If data are missing for some cases, or if reasons for dropout are not reported, we will contact the trial authors to request the full data. In the event that a study does not report the standard deviation (SD) for the continuous outcome and the SD cannot be calculated from the reported data (such as standard error, confidence interval, p value), we will write to the authors to request the data for SD. If the SD data are not available from authors, we will use SD from a similar study that has a similar study population. We will prefer to use the final values of a continuous outcome for a given follow-up. If the final values are not available but the difference between the end and the start of the study, we will write to authors to request the final values. If the final values are not available, we will use the difference or rate of change. We will use the data based on intention to treat analysis. If the data for intention to treat analysis is not given in the study, we will create our own intention to treat analysis for participants with known outcomes. If there is significant attrition between the randomized participants vs. participants completing the study, we will include such a study but will investigate further with sensitivity analysis.

Assessment of risk of bias in included studies

The risk of bias in studies will be evaluated using the Cochrane risk of bias (ROB 2.0) and Cochrane ROBINS-I for non-randomized and observational studies [19,20]. Two review authors will independently evaluate and agree on the risk of bias for the individual studies for an outcome. The discussion will resolve any disagreements, and if no agreement can be made, the senior review author will be consulted. We will assess the effect of assignment to intervention (the intention-to-treat effect) for randomized trials by addressing five domains of signaling questions in ROB-2 including: bias arising from the randomization process, bias due to deviations from intended interventions, bias due to missing outcome data, bias in measurement of the outcome and bias in the selection of the reported result. Each domain will receive a ranking of one of the following: low risk of bias, some concerns of bias, or high risk of bias. We will include quotes from the study for each signaling question as evidence for our ranking decision. The overall risk of bias will be determined based on the lowest ranking for individual domains. For example, if only one domain is ranked 'some concerns,' then the overall risk of bias will be 'some concerns'.

For peer review only - http://bmjopen.bmj.com/site/about/guidelines.xhtml

Data Synthesis

The findings from the systematic review will be reported both qualitatively and quantitatively. A narrative synthesis will be used to report the characteristics of all included studies. We will also narratively describe the data in a table for the secondary objective of this review (table 2). A random-effects meta-analysis will be employed when at least two studies possess sufficient clinical and methodological uniformity for synthesis for the primary objective of the review. We will use the generic inverse method to pool the studies in the meta-analysis. The software RevMan will be utilized for statistical analysis[21]. Dichotomous outcomes will be assessed using relative risk effect sizes and presented with 95% confidence intervals. In the case of morbidity outcomes, we will combine all available data whenever possible if outcomes are measured in different ways. For example, we will include all types of diarrhea (mild, moderate, and severe) as a dichotomous value (yes/no) if participants had greater than 3 instances of loose stools per day. We will include the occurrence of anemia, iron deficiency anemia, and pneumonia throughout the study as dichotomous values (yes/no). For continuous outcomes, we will pool the data to obtain an average mean difference and report it with its 95% CI. In the event that data are reported in different units (e.g., few studies report weight in kg and the others report in Z scores), we will use a standardized mean difference effect size and report it with its 95% CI. We will pool the data separately from randomized and observational studies. If an observational study report both adjusted and unadjusted values, we will use the adjusted values for meta-analysis.

We will consider the following pairs of comparisons:
High iron content in RUTF vs. WHO standard iron content in RUTF

The Grading of Recommendations Assessment, Development, and Evaluation (GRADE) approach will be used to evaluate overall evidence quality using the software GRADEpro[22]. The GRADE approach is a comprehensive framework used to assess the overall quality of evidence for an outcome using characteristics such as study design, heterogeneity, directness of evidence, risk of bias, publication bias, and precision of effect estimates[23]. The results of the GRADE assessment will be included in a summary of findings table. The table will contain quality ratings characterized as very low (we have very little confidence in the effect estimate), low (we have limited confidence in the effect estimate), moderate (we have moderate confidence in the effect estimate; the true effect is likely close to

For peer review only

the estimate of the effect), or high based (we have high confidence that the true effect lies close to that of the estimate of the effect) on the primary outcomes of each study (Table 3).

Patient and public involvement

No patient or public involvement was considered in the preparation of this protocol.

UNIT OF ANALYSIS ISSUES

Multiple-arm trial

Studies with multiple treatment arms will be included if eligible. For multiple-arm trials, we will include data so that the only difference between the groups is the use of iron in RUTF.

Cluster trials

Cluster assignment trials will be analyzed together with individually randomized trials. We will use the cluster adjusted values; if the trial results are not adjusted for cluster design, we will adjust the result by methods given in the Cochrane[24] handbook for systematic reviews.

Assessment of heterogeneity

Statistical heterogeneity of effect sizes within any given meta-analysis will be assessed using the χ^2, I^2, and tau statistics. We will assess statistical heterogeneity by visual inspection of forest plots, performing

the χ^2test (assessing the p-value), and calculating the Tau^2 and I^2 statistic. Statistical heterogeneity will be considered significant if the p-value is less than 0.10, I^2 value exceeds 50%, and the examination of forest plots shows substantial variability in the effect of the intervention. We will perform subgroup analysis to determine the reasons for any identified statistical heterogeneity.

Assessment of reporting bias

Small study and publication bias will be assessed using funnel plots and regression tests for funnel plot asymmetry when a meta-analysis includes at least ten studies.

Subgroup analyses

- Settings: Country: Low-income country vs. middle-income country vs. high-income country

For peer review only

- Type of RUTF: Standard RUTF vs. Non-standard
- Role of RUTF: RUTF as main treatment vs. RUTF as a supplementary intervention
- Type of participants: Studies that included children with HIV vs. Studies with children without HIV.
- Age: <24 months vs. 24-59 months vs. > 59 months
- Hospitalization: Children hospitalized (due to medical complication) prior to starting RUTF vs. Children not hospitalized prior to starting RUTF
- Iron compound (formulation/chemical compound and amount)
- Dose: The intervention groups with dose of iron > standard WHO RUTF Vs. Intervention group receiving dose lower the Standard WHO RUTF

Sensitivity analyses:

-Studies with a high risk of bias

-Random vs. fixed effect model

Amendments

We will do the literature searches, screening of titles, selection of studies, data extraction and analysis according to the aforementioned plan described in this protocol. If we do any additional analysis or change any of the a priori strategies, we will clearly describe that in the methods section.

Consent for publication

The authors give consent for the publication of the review.

Competing interests

None declared.

Availability of data and materials

We will provide data available for review by the editors and peer reviewers and provide raw data for the general public on request.

Funding

For peer review only

This work is funded by World Health Organization grant no 202712614. WHO also provided technical support for this work.

For peer review only

Acknowledgements

We are very thankful to Allison Daniel, Jaden Bendabenda, Lisa Rogers, and Maria De Las Nieves Garcia Casal for their input to improve this protocol.

Authors' contributions

AI and MF wrote the first draft of the manuscript. AS and OT developed the search strategy. ET-S

provided the support for methodology. ZAB, FC and JD provided the support for the content being evaluated in the review. All authors edited and reviewed the manuscript.

ETHICS AND DISSEMINATION

This is a systematic review and will not involve direct contact with human subjects. The findings of this review will be published in a peer-review journal and will guide the WHO's recommendation on optimal iron content in ready-to-use therapeutic foods for the treatment of severe acute malnutrition in children aged 6-59 months.

For peer review only

REFERENCES

1. United Nations Children's Fund (UNICEF), World Health Organization, International Bank for Reconstruction and Development/The World Bank. *Levels and trends in child malnutrition: key findings of the 2021 edition of the joint child malnutrition estimates*. Geneva: World Health Organization; 2021.
2. nnpfm RBR. Human milk lyophilisate - Phase 1 and 2 study: safety, tolerability and effectiveness. *http://wwwwhoint/trialsearch/Trial2aspx?TrialID=RBR-8nnpfm*. 2019.
3. Akomo P, Bahwere P, Murakami H, et al. Soya, maize and sorghum ready-to-use therapeutic foods are more effective in correcting anaemia and iron deficiency than the standard ready-to-use therapeutic food: randomized controlled trial. *BMC Public Health.* 2019;19(1):806.
4. Savadogo LGB, Zoetaba I, Ilboudo B, Kinda M, Donnen P. Impact of anemia on mortality and nutritional recovery among hospitalized severely malnourished children in Burkina Faso. *Open Journal of Pediatrics.* 2014;2014.
5. Thakur N, Chandra J, Pemde H, Singh V. Anemia in severe acute malnutrition. *Nutrition.* 2014;30(4):440-442.
6. Kangas ST, Salpeteur C, Nikiema V, et al. Vitamin A and iron status of children before and after treatment of uncomplicated severe acute malnutrition. *Clin Nutr.* 2020;39(11):3512-3519.
7. Babirekere-Iriso E, Mortensen CG, Mupere E, et al. Changes in whole-blood PUFA and their predictors during recovery from severe acute malnutrition. *Br J Nutr.* 2016;115(10):1730-1739.
8. Jones KD, Ali R, Khasira MA, et al. Ready-to-use therapeutic food with elevated n-3 polyunsaturated fatty acid content, with or without fish oil, to treat severe acute malnutrition: a randomized controlled trial. *BMC Med.* 2015;13:93.
9. WHO W, UNSCN, UNICEF. Community-based management of severe acute malnutrition. A joint statement by the World Health Organization, World Food Programme, United Nations Standing Committee on Nutrition, United Nations Children's Fund. Geneva, World Health Organization. 2007.
10. Weisstaub G, Medina M, Pizarro F, Araya M. Copper, iron, and zinc status in children with moderate and severe acute malnutrition recovered following WHO protocols. *Biol Trace Elem Res.* 2008;124(1):1-11.

For peer review only

11. Lopriore C, Guidoum Y, Briend A, Branca F. Spread fortified with vitamins and minerals induces catch-up growth and eradicates severe anemia in stunted refugee children aged 3-6 y. *Am J Clin Nutr.* 2004;80(4):973-981.
12. Bahwere P, Balaluka B, Wells JC, et al. Cereals and pulse-based ready-to-use therapeutic food as an alternative to the standard milk- and peanut paste-based formulation for treating severe acute malnutrition: a noninferiority, individually randomized controlled efficacy clinical trial. *Am J Clin Nutr.* 2016;103(4):1145-1161.
13. Soofi S, Cousens S, Iqbal SP, et al. Effect of provision of daily zinc and iron with several micronutrients on growth and morbidity among young children in Pakistan: a cluster-randomised trial. *Lancet.* 2013;382(9886):29-40.
14. Paganini D, Zimmermann MB. The effects of iron fortification and supplementation on the gut microbiome and diarrhea in infants and children: a review. *Am J Clin Nutr.* 2017;106(Suppl 6):1688S-1693S.
15. Sazawal S, Black RE, Ramsan M, et al. Effects of routine prophylactic supplementation with iron and folic acid on admission to hospital and mortality in preschool children in a high malaria transmission setting: community-based, randomised, placebo-controlled trial. *Lancet.* 2006;367(9505):133-143.
16. Page MJ, McKenzie JE, Bossuyt PM, et al. The PRISMA 2020 statement: an updated guideline for reporting systematic reviews. *BMJ.* 2021;372:n71.
17. Organization WH. Management of severe malnutrition : a manual for physicians and other senior health workers. 1999.
18. Covidence systematic review software vhi, Melbourne, Australia. Available: www.covidence.org.
19. Sterne JA, Hernan MA, Reeves BC, et al. ROBINS-I: a tool for assessing risk of bias in non-randomised studies of interventions. *BMJ.* 2016;355:i4919.
20. Sterne JAC, Savovic J, Page MJ, et al. RoB 2: a revised tool for assessing risk of bias in randomised trials. *BMJ.* 2019;366:l4898.
21. Review Manager (RevMan) [Computer program]. Version 5.3. Copenhagen: The Nordic Cochrane Centre TCC. Version 5.3. Copenhagen: The Nordic Cochrane Centre, The Cochrane Collaboration. Published 2014. Accessed. .
22. GRADEpro GDT: GRADEpro Guideline Development Tool [Software]. McMaster University, 2020 (developed by Evidence Prime, Inc.). Available from gradepro.org.

For peer review only

23. Guyatt G, Oxman AD, Akl EA, et al. GRADE guidelines: 1. Introduction-GRADE evidence profiles and summary of findings tables. *J Clin Epidemiol.* 2011;64(4):383-394.

24. Higgins JPT TJ CJ, Cumpston M. Cochrane handbook for systematic reviews of interventions version 6.2, 2021. https://training.cochrane.org/handbook/PDF/v6.2.

25. (EPOC) CEPaOoC. What study designs can be considered for inclusion in an EPOC review and what should they be called? Avaialble at https://epoc.cochrane.org/sites/epoc.cochrane.org/files/public/uploads/EPOC%20Study%20Designs%20About.pdf. July 2021.

For peer review only

Table 1: The definitions of Eligible Study Designs

Study Designs	Definition	Notes
Randomized controlled trial (RCT) OR randomized trial	An experimental study in which people are allocated to different interventions using methods that are random.”	We will include both individual and cluster randomized trials. In individual randomized trials, the randomized is done at the individual levels while in cluster randomized trials, the randomized is based on cluster or groups of individuals. We will also consider factorial design trials where multiple interventions are studies in the same trial.
Non-randomised controlled trial (NRCT) OR non-randomised trial	“An experimental study in which people are allocated to different interventions using methods that are not random.”	We will avoid using the term quasi experimental studies as it means differently by different authors. We will exclude the experimental studies where there was no control group.
Controlled before-after study (CBA)	“A study in which observations are made before and after the implementation of an intervention, both in a group that receives the intervention and in a control group that does not.”	We will require two minimum criteria for the inclusion of CBAs.<br>-Data Collection: We will include CBAs if the data for the intervention and control groups were collected prospectively in the same time frame<br>-Choice of Control: We will include CBAs that include a control at a second site to avoid contamination of the intervention to the control group if the settings and populations are the same for the intervention and control groups.

Footnotes: The definitions of study designs were adopted from The Cochrane Effective Practice and Organization of Care (EPOC) group[25]

For peer review only

Table 2: Sample table to describe the studies that address delivery mechanism to increase the bioavailability of iron via RUTF in children 6 months and older with severe acute malnutrition

Study	Study Participants	Formulations used	Comparison group	Outcomes	Notes

For peer review only

Table 3: GRADE Assessment

Study Design	Quality of Evidence	Lower Certainty Score If	Higher Certainty Score If
Randomized trial	High	Risk of Bias<br>-1 Serious<br>-2 Very serious<br><br>Inconsistency<br>-1 Serious<br>-2 Very serious<br><br>Indirectness<br>-1 Serious<br>-2 Very serious<br><br>Imprecision<br>-1 Serious	Large effect<br>+1 Large<br>+2 Very large<br><br>Dose-response<br>+1 evidence of a gradient<br><br>All plausible confounding would:<br>+1 Reduce a demonstrated effect<br><br>+1 Suggest a spurious effect when results show no effect
	Moderate		
Observational study	Low		

	Very Low	-2 Very serious<br><br>Publication Bias<br>-1 Likely<br>-2 Very likely	

For peer review only

Appendix 1: Search strategies for different electronic databases

PubMed

("Iron"[MeSH Terms] OR "iron, dietary"[MeSH Terms] OR "Iron"[Title/Abstract] OR "56Fe"[Title/Abstract] OR "Fe"[Title/Abstract] OR "ferro"[Title/Abstract] OR "ferrum"[Title/Abstract]) AND ("food, formulated"[MeSH Terms] OR "food, fortified"[MeSH Terms] OR "RUTF"[Title/Abstract] OR "RUTFs"[Title/Abstract] OR "ready to use therapeutic food*"[Title/Abstract] OR "ready to use therapeutic feed*"[Title/Abstract] OR "therapeutic food*"[Title/Abstract] OR "therapeutic diet*"[Title/Abstract] OR "enriched food*"[Title/Abstract] OR "enriched diet*"[Title/Abstract] OR "fortified food*"[Title/Abstract] OR "fortified diet*"[Title/Abstract] OR "supplemented food*"[Title/Abstract] OR "supplemental food*"[Title/Abstract] OR "supplementary food*"[Title/Abstract] OR "supplement food*"[Title/Abstract] OR "supplemented diet*"[Title/Abstract] OR "supplemental diet*"[Title/Abstract] OR "supplementary diet*"[Title/Abstract] OR "supplement diet*"[Title/Abstract] OR "RUSF"[Title/Abstract] OR "RUSFs"[Title/Abstract] OR "ready to use supplemental food*"[Title/Abstract] OR "ready to use supplementary food*"[Title/Abstract] OR "lipid based nutrient supplement*"[Title/Abstract] OR "LNS"[Title/Abstract]) AND ("Severe Acute Malnutrition"[MeSH Terms] OR "Malnutrition"[MeSH Terms] OR "Cachexia"[MeSH Terms] OR "Wasting Syndrome"[MeSH Terms] OR "Severe Acute Malnutrition"[Title/Abstract] OR "SAM"[Title/Abstract] OR "Malnutrition"[Title/Abstract] OR "malnourish*"[Title/Abstract] OR "Cachexia"[Title/Abstract] OR "nutritional deficien*"[Title/Abstract] OR "undernutrition"[Title/Abstract] OR "deficient nutrition"[Title/Abstract] OR "undernourish*"[Title/Abstract] OR "wasting"[Title/Abstract] OR "wasted"[Title/Abstract]) AND ("Infant"[MeSH Terms] OR "Child"[MeSH Terms] OR "infant*"[Title/Abstract] OR "infancy"[Title/Abstract] OR "baby"[Title/Abstract] OR "babies"[Title/Abstract] OR "newborn*"[Title/Abstract] OR "neonat*"[Title/Abstract] OR "neo nat*"[Title/Abstract] OR "child*"[Title/Abstract] OR "toddler*"[Title/Abstract] OR "youth"[Title/Abstract] OR "juvenile*"[Title/Abstract] OR "girl*"[Title/Abstract] OR "boy"[Title/Abstract] OR "boys"[Title/Abstract] OR "preschool*"[Title/Abstract] OR "pre school*"[Title/Abstract])

CINAHL

S1 (MH "Infant+")
S2 (MH "Child+")
S3 TI (infant* OR infancy OR baby OR babies OR newborn* OR neonat* OR "neo nat*" OR child* OR toddler* OR youth OR juvenile* OR girl* OR boy OR boys OR preschool* OR "pre school*")
S4 AB (infant* OR infancy OR baby OR babies OR newborn* OR neonat* OR "neo nat*" OR child* OR toddler* OR youth OR juvenile* OR girl* OR boy OR boys OR preschool* OR "pre school*")
S5 S1 OR S2 OR S3 OR S4
S6 (MH "Iron")
S7 TI (iron OR 56Fe OR Fe Or ferro OR ferrum)
S8 AB (iron OR 56Fe OR Fe Or ferro OR ferrum)
S9 S6 OR S7 OR S8
S10 (MH "Food, Formulated")
S11 (MH "Food, Fortified")
S12 TI (RUTF OR RUTFs OR "ready to use therapeutic food*" OR "ready to use therapeutic feed*" OR RUSF OR RUSFs OR "ready to use supplemental food*" OR "ready to use supplementary food*" OR "lipid based nutrient supplement*" OR LNS)
S13 AB (RUTF OR RUTFs OR "ready to use therapeutic food*" OR "ready to use therapeutic feed*" OR RUSF OR RUSFs OR "ready to use supplemental food*" OR "ready to use supplementary food*" OR "lipid based nutrient supplement*" OR LNS)
S14 TI((enriched OR fortified OR supplement* OR therapeutic) W1 (food* OR diet*))
S15 AB((enriched OR fortified OR supplement* OR therapeutic) W1 (food* OR diet*))
S16 S10 OR S11 OR S12 OR S13 OR S14 OR S15
S17 (MH "Cachexia")
S18 (MH "Malnutrition")
S19 (MH "Wasting Syndrome")

For peer review only

S20 TI ("Severe Acute Malnutrition" OR SAM OR Malnutrition OR malnourish* OR Cachexia OR undernutrition OR undernourish* OR wasting OR wasted)
S21 AB ("Severe Acute Malnutrition" OR SAM OR Malnutrition OR malnourish* OR Cachexia OR undernutrition OR undernourish* OR wasting OR wasted)
S22 TI (nutrition* N1 deficien*)
S23 AB (nutrition* N1 deficien*)
S24 S17 OR S18 OR S19 OR S20 OR S21 OR S22 OR S23
S25 S5 AND S9 AND S16 AND S24
S26 S25 Exclude MEDLINE records

Embase

#1 'iron'/exp OR 'iron intake'/exp
#2 iron:ti,ab OR 56fe:ti,ab OR fe:ti,ab OR ferro:ti,ab OR ferrum:ti,ab
#3 #1 OR #2
#4 'ready to use therapeutic food'/exp OR 'fortified food'/exp OR 'ready to use supplementary food'/exp OR 'lipid based nutrient supplement'/exp OR 'dietary supplement'/exp
#5 ((enriched OR fortified OR supplement* OR therapeutic) NEXT/1 (food* OR diet*)):ti,ab
#6 rutf:ti,ab OR rutfs:ti,ab OR 'ready to use therapeutic food*':ti,ab OR 'ready to use therapeutic feed*':ti,ab OR rusf:ti,ab OR rusfs:ti,ab OR 'ready to use supplemental food*':ti,ab OR 'ready to use supplementary food*':ti,ab OR 'lipid based nutrient supplement*':ti,ab OR lns:ti,ab
#7 #4 OR #5 OR #6
#8 'malnutrition'/exp OR 'wasting syndrome'/exp OR 'cachexia'/exp
#9 (nutrition* NEAR/1 deficien*):ti,ab
#10 'severe acute malnutrition':ti,ab OR sam:ti,ab OR malnutrition:ti,ab OR malnourish*:ti,ab OR cachexia:ti,ab OR undernutrition:ti,ab OR undernourish*:ti,ab OR wasting:ti,ab OR wasted:ti,ab
#11 #8 OR #9 OR #10
#12 'infant'/exp OR 'infancy'/exp OR 'baby'/exp OR 'newborn'/exp OR 'toddler'/exp OR 'child'/exp OR 'preschool child'/exp OR 'juvenile'/exp OR 'girl'/exp OR 'boy'/exp
#13 infant*:ti,ab OR infancy:ti,ab OR baby:ti,ab OR babies:ti,ab OR newborn*:ti,ab OR neonat*:ti,ab OR 'neo nat*':ti,ab OR child*:ti,ab OR toddler*:ti,ab OR youth:ti,ab OR juvenile*:ti,ab OR girl*:ti,ab OR boy:ti,ab OR boys:ti,ab OR preschool*:ti,ab OR 'pre school*':ti,ab
#14 #12 OR #13
#15 #3 AND #7 AND #11 AND #14
#16 #15 NOT [medline]/lim

SCOPUS

(TITLE-ABS (iron OR 56fe OR fe OR ferro OR ferrum)) AND ((TITLE-ABS (enriched OR fortified OR supplement* OR therapeutic) PRE/1 TITLE-ABS (food* OR diet*)) OR (TITLE-ABS (rutf OR rutfs OR "ready to use therapeutic food*" OR "ready to use therapeutic feed*" OR rusf OR rusfs OR "ready to use supplemental food*" OR "ready to use supplementary food*" OR "lipid based nutrient supplement*" OR lns))) AND ((TITLE-ABS (nutrition* W/1 deficien*)) OR (TITLE-ABS ("Severe Acute Malnutrition" OR sam OR malnutrition OR malnourish* OR cachexia OR undernutrition OR undernourish* OR wasting OR wasted))) AND (TITLE-ABS (infant* OR infancy OR baby OR babies OR newborn* OR neonat* OR neo-nat* OR child* OR toddler* OR youth OR juvenile* OR girl* OR boy OR boys OR preschool* OR pre-school*)) AND NOT INDEX (medline)

CENTRAL

#1 MeSH descriptor: [Iron] in all MeSH products
#2 MeSH descriptor: [Iron, Dietary] explode all trees
#3 Iron:ti,ab OR 56Fe:ti,ab OR Fe:ti,ab OR ferro:ti,ab OR ferrum:ti,ab
#4 #1 OR #2 OR #3
#5 MeSH descriptor: [Food, Formulated] explode all trees
#6 MeSH descriptor: [Food, Fortified] explode all trees
#7 ((enriched OR fortified OR supplement* OR therapeutic) NEXT (food* OR diet*)):ti,ab

For peer review only

#8 (ready NEXT to NEXT use NEXT therapeutic NEXT food*):ti,ab
#9 (ready NEXT to NEXT use NEXT therapeutic NEXT feed*):ti,ab
#10 (ready NEXT to NEXT use NEXT supplemental NEXT food*):ti,ab
#11 (ready NEXT to NEXT use NEXT supplementary NEXT food*):ti,ab
#12 (lipid NEXT based NEXT nutrient NEXT supplement*):ti,ab
#13 rutf:ti,ab OR rutfs:ti,ab OR rusf:ti,ab OR rusfs:ti,ab OR lns:ti,ab
#14 #5 OR #6 OR #7 OR #8 OR #9 OR #10 OR #11 OR #12 OR #13
#15 MeSH descriptor: [Severe Acute Malnutrition] explode all trees
#16 MeSH descriptor: [Malnutrition] explode all trees
#17 MeSH descriptor: [Cachexia] explode all trees
#18 MeSH descriptor: [Wasting Syndrome] explode all trees
#19 (nutrition* NEAR/1 deficien*):ti,ab
#20 (severe NEXT acute NEXT malnutrition):ti,ab
#21 sam:ti,ab OR malnutrition:ti,ab OR malnourish*:ti,ab OR cachexia:ti,ab OR undernutrition:ti,ab OR undernourish*:ti,ab OR wasting:ti,ab OR wasted:ti,ab
#22 #15 OR #16 OR #17 OR #18 OR #19 OR #20 OR #21
#23 MeSH descriptor: [Infant] explode all trees
#24 MeSH descriptor: [Child] explode all trees
#25 infant*:ti,ab OR infancy:ti,ab OR baby:ti,ab OR babies:ti,ab OR newborn*:ti,ab OR neonat*:ti,ab OR neo NEXT nat*':ti,ab OR child*:ti,ab OR toddler*:ti,ab OR youth:ti,ab OR juvenile*:ti,ab OR girl*:ti,ab OR boy:ti,ab OR boys:ti,ab OR preschool*:ti,ab OR pre NEXT school*:ti,ab
#26 #23 OR #24 OR #25
#27 #4 AND #14 AND #22 AND #26
#28 "accession number" near pubmed
#29 #27 NOT #28

Web of Science

#1 TI=(iron OR 56fe OR fe OR ferro OR ferrum)
#2 AB=(iron OR 56fe OR fe OR ferro OR ferrum)
#3 #1 OR #2
#4 TI=(RUTF OR RUTFs OR "ready to use therapeutic food*" OR "ready to use therapeutic feed*" OR "therapeutic food*" OR "therapeutic diet*" OR "enriched food*" OR "enriched diet*" OR "fortified food*" OR "fortified diet*" OR "supplemented food*" OR "supplemental food*" OR "supplementary food*" OR "supplement food*" OR "supplemented diet*" OR "supplemental diet*" OR "supplementary diet*" OR "supplement diet*" OR RUSF OR RUSFs OR "ready to use supplemental food*" OR "ready to use supplementary food*" OR "lipid based nutrient supplement*" OR LNS)
#5 AB=(RUTF OR RUTFs OR "ready to use therapeutic food*" OR "ready to use therapeutic feed*" OR "therapeutic food*" OR "therapeutic diet*" OR "enriched food*" OR "enriched diet*" OR "fortified food*" OR "fortified diet*" OR "supplemented food*" OR "supplemental food*" OR "supplementary food*" OR "supplement food*" OR "supplemented diet*" OR "supplemental diet*" OR "supplementary diet*" OR "supplement diet*" OR RUSF OR RUSFs OR "ready to use supplemental food*" OR "ready to use supplementary food*" OR "lipid based nutrient supplement*" OR LNS)
#6 #4 OR #5
#7 TI=(nutrition* NEAR/1 deficien*)
#8 AB=(nutrition* NEAR/1 deficien*)
#9 TI=("Severe Acute Malnutrition" OR sam OR malnutrition OR malnourish* OR cachexia OR undernutrition OR undernourish* OR wasting OR wasted)
#10 AB=("Severe Acute Malnutrition" OR sam OR malnutrition OR malnourish* OR cachexia OR undernutrition OR undernourish* OR wasting OR wasted)
#11 #7 OR #8 OR #9 OR #10
#12 TI=(infant* OR infancy OR baby OR babies OR newborn* OR neonat* OR "neo nat*" OR child* OR toddler* OR youth OR juvenile* OR girl* OR boy OR boys OR preschool* OR "pre school*")

For peer review only

#13 AB=(infant* OR infancy OR baby OR babies OR newborn* OR neonat* OR "neo nat*" OR child* OR toddler* OR youth OR juvenile* OR girl* OR boy OR boys OR preschool* OR "pre school*")
#14 #12 OR #13
#15 #3 AND #6 AND #11 AND #14

LILACS

(mh:("Iron")) OR ((mh:("iron, dietary"))) OR ((ti:(iron OR 56fe OR fe OR ferro OR ferrum))) OR ((ab:(iron OR 56fe OR fe OR ferro OR ferrum))) AND ((mh:("food, formulated")) OR ((mh:("food, fortified"))) OR ((ti:(rutf OR rutfs OR "ready to use therapeutic food*" OR "ready to use therapeutic feed*" OR "therapeutic food*" OR "therapeutic diet*" OR "enriched food*" OR "enriched diet*" OR "fortified food*" OR "fortified diet*" OR "supplemented food*" OR "supplemental food*" OR "supplementary food*" OR "supplement food*" OR "supplemented diet*" OR "supplemental diet*" OR "supplementary diet*" OR "supplement diet*" OR rusf OR rusfs OR "ready to use supplemental food*" OR "ready to use supplementary food*" OR "lipid based nutrient supplement*" OR lns))) OR ((ab:(rutf OR rutfs OR "ready to use therapeutic food*" OR "ready to use therapeutic feed*" OR "therapeutic food*" OR "therapeutic diet*" OR "enriched food*" OR "enriched diet*" OR "fortified food*" OR "fortified diet*" OR "supplemented food*" OR "supplemental food*" OR "supplementary food*" OR "supplement food*" OR "supplemented diet*" OR "supplemental diet*" OR "supplementary diet*" OR "supplement diet*" OR rusf OR rusfs OR "ready to use supplemental food*" OR "ready to use supplementary food*" OR "lipid based nutrient supplement*" OR lns)))) AND ((mh:("Severe Acute Malnutrition")) OR ((mh:("Malnutrition"))) OR ((mh:("cachexia"))) OR ((mh:("wasting syndrome"))) OR ((ti:("Severe Acute Malnutrition" OR sam OR malnutrition OR malnourish* OR cachexia OR "nutritional deficien*" OR undernutrition OR "deficient nutrition" OR undernourish* OR wasting OR wasted))) OR ((ab:("Severe Acute Malnutrition" OR sam OR malnutrition OR malnourish* OR cachexia OR "nutritional deficien*" OR undernutrition OR "deficient nutrition" OR undernourish* OR wasting OR wasted)))) AND ((mh:(infant)) OR ((mh:(child))) OR ((ti:(infant* OR infancy OR baby OR babies OR newborn* OR neonat* OR "neo nat*" OR child* OR toddler* OR youth OR juvenile* OR girl* OR boy OR boys OR preschool* OR "pre school*"))) OR ((ab:(infant* OR infancy OR baby OR babies OR newborn* OR neonat* OR "neo nat*" OR child* OR toddler* OR youth OR juvenile* OR girl* OR boy OR boys OR preschool* OR "pre school*")))) AND (db:("LILACS"))

Global Index Medicus

((mh:("Iron")) OR ((mh:("iron, dietary"))) OR ((ti:(iron OR 56fe OR fe OR ferro OR ferrum))) OR ((ab:(iron OR 56fe OR fe OR ferro OR ferrum))) AND ((mh:("food, formulated")) OR ((mh:("food, fortified"))) OR ((ti:(rutf OR rutfs OR "ready to use therapeutic food*" OR "ready to use therapeutic feed*" OR "therapeutic food*" OR "therapeutic diet*" OR "enriched food*" OR "enriched diet*" OR "fortified food*" OR "fortified diet*" OR "supplemented food*" OR "supplemental food*" OR "supplementary food*" OR "supplement food*" OR "supplemented diet*" OR "supplemental diet*" OR "supplementary diet*" OR "supplement diet*" OR rusf OR rusfs OR "ready to use supplemental food*" OR "ready to use supplementary food*" OR "lipid based nutrient supplement*" OR lns))) OR ((ab:(rutf OR rutfs OR "ready to use therapeutic food*" OR "ready to use therapeutic feed*" OR "therapeutic food*" OR "therapeutic diet*" OR "enriched food*" OR "enriched diet*" OR "fortified food*" OR "fortified diet*" OR "supplemented food*" OR "supplemental food*" OR "supplementary food*" OR "supplement food*" OR "supplemented diet*" OR "supplemental diet*" OR "supplementary diet*" OR "supplement diet*" OR rusf OR rusfs OR "ready to use supplemental food*" OR "ready to use supplementary food*" OR "lipid based nutrient supplement*" OR lns)))) AND ((mh:("Severe Acute Malnutrition")) OR ((mh:("Malnutrition"))) OR ((mh:("cachexia"))) OR ((mh:("wasting syndrome"))) OR ((ti:("Severe Acute Malnutrition" OR sam OR malnutrition OR malnourish* OR cachexia OR "nutritional deficien*" OR undernutrition OR "deficient nutrition" OR undernourish* OR wasting OR wasted))) OR ((ab:("Severe Acute Malnutrition" OR sam OR malnutrition OR malnourish* OR cachexia OR "nutritional deficien*" OR undernutrition OR "deficient nutrition" OR undernourish* OR wasting OR wasted)))) AND ((mh:(infant)) OR ((mh:(child))) OR ((ti:(infant* OR infancy OR baby OR babies OR newborn* OR neonat* OR "neo nat*" OR child* OR toddler* OR youth OR juvenile* OR girl* OR boy OR boys OR preschool* OR "pre school*"))) OR ((ab:(infant* OR infancy OR baby OR babies OR newborn* OR neonat* OR "neo nat*" OR child* OR toddler* OR youth OR juvenile* OR girl* OR boy OR boys OR preschool* OR "pre school*"))))) AND (collection_gim:("IMSEAR" OR "IMEMR" OR "WPRIM"))

Routine antibiotics for infants less than six months of age with growth failure/faltering. A protocol for the systematic review

Aamer Imdad, Fanny F Chen, Melissa Francois, Emily Tanner-Smith, Abigail Smith, Olivia J Tsistinas, Jai Das, Zulfiqar Ahmed Bhutta

PRISMA-P (Preferred Reporting Items for Systematic review and Meta-Analysis Protocols) 2015 checklist: recommended items to address in a systematic review protocol*

Section and topic	Item No	Checklist item	This manuscript
**ADMINISTRATIVE INFORMATION**			
Title:			
Identification	1a	Identify the report as a protocol of a systematic review	Done
Update	1b	If the protocol is for an update of a previous systematic review, identify as such	
Registration	2	If registered, provide the name of the registry (such as PROSPERO) and registration number	Application pending
Authors:			
Contact	3a	Provide name, institutional affiliation, e-mail address of all protocol authors; provide physical mailing address of corresponding author	Done
Contributions	3b	Describe contributions of protocol authors and identify the guarantor of the review	
Amendments	4	If the protocol represents an amendment of a previously completed or published protocol, identify as such and list changes; otherwise, state plan for documenting important protocol amendments	Done
Support:			
Sources	5a	Indicate sources of financial or other support for the review	
Sponsor	5b	Provide name for the review funder and/or sponsor	Done
Role of sponsor or funder	5c	Describe roles of funder(s), sponsor(s), and/or institution(s), if any, in developing the protocol	
**INTRODUCTION**			
Rationale	6	Describe the rationale for the review in the context of what is already known	
Objectives	7	Provide an explicit statement of the question(s) the review will address with reference to participants, interventions, comparators, and outcomes (PICO)	Done
**METHODS**			

For peer review only

Eligibility criteria	8	Specify the study characteristics (such as PICO, study design, setting, time frame) and report characteristics (such as years considered, language, publication status) to be used as criteria for eligibility for the review	Done
Information sources	9	Describe all intended information sources (such as electronic databases, contact with study authors, trial registers or other grey literature sources) with planned dates of coverage	Done
Search strategy	10	Present draft of search strategy to be used for at least one electronic database, including planned limits, such that it could be repeated	Done
Study records:			Done
Data management	11a	Describe the mechanism(s) that will be used to manage records and data throughout the review	
Selection process	11b	State the process that will be used for selecting studies (such as two independent reviewers) through each phase of the review (that is, screening, eligibility and inclusion in meta-analysis)	
Data collection process	11c	Describe planned method of extracting data from reports (such as piloting forms, done independently, in duplicate), any processes for obtaining and confirming data from investigators	
Data items	12	List and define all variables for which data will be sought (such as PICO items, funding sources), any pre-planned data assumptions and simplifications	
Outcomes and prioritization	13	List and define all outcomes for which data will be sought, including prioritization of main and additional outcomes, with rationale	Done
Risk of bias in individual studies	14	Describe anticipated methods for assessing risk of bias of individual studies, including whether this will be done at the outcome or study level, or both; state how this information will be used in data synthesis	Done
Data synthesis	15a	Describe criteria under which study data will be quantitatively synthesised	
	15b	If data are appropriate for quantitative synthesis, describe planned summary measures, methods of handling data and methods of combining data from studies, including any planned exploration of consistency (such as I^2, Kendall's τ)	Done
	15c	Describe any proposed additional analyses (such as sensitivity or subgroup analyses, meta-regression)	
	15d	If quantitative synthesis is not appropriate, describe the type of summary planned	
Meta-bias(es)	16	Specify any planned assessment of meta-bias(es) (such as publication bias across studies, selective reporting within studies)	Done
Confidence in cumulative evidence	17	Describe how the strength of the body of evidence will be assessed (such as GRADE)	Done

For peer review only

BMJ Open

Optimal iron content in ready-to-use therapeutic foods for the treatment of severe acute malnutrition in the community settings. A protocol for the systematic review and meta-analysis

Journal:	*BMJ Open*
Manuscript ID	bmjopen-2021-057389.R1
Article Type:	Protocol
Date Submitted by the Author:	21-Jan-2022
Complete List of Authors:	Imdad, Aamer; SUNY Upstate Medical University, Pediatrics<br>François, Melissa; SUNY Upstate Medical University, College of Medicine<br>Chen, Fanny F; SUNY Upstate Medical University<br>Smith, Abigail; SUNY Upstate Medical University, Library Sciences<br>Tsistinas, Olivia; SUNY Upstate Medical University, Library sciences<br>Tanner-Smith, Emily; University of Oregon, College of Education<br>Das, Jai K.; The Aga Khan University<br>Bhutta, Zulfiqar; The Aga Khan University
**Primary Subject Heading**:	Paediatrics
Secondary Subject Heading:	Global health, Nutrition and metabolism, Gastroenterology and hepatology
Keywords:	PAEDIATRICS, NUTRITION & DIETETICS, PUBLIC HEALTH

SCHOLARONE™
Manuscripts

Optimal iron content in ready-to-use therapeutic foods for the treatment of severe acute malnutrition in the community settings. A protocol for the systematic review and meta-analysis

Aamer Imdad, Melissa François, Fanny F Chen, Abigail Smith, Olivia J Tsistinas, Emily Tanner-Smith, Jai K Das, Zulfiqar Ahmed Bhutta

Authors' information

Corresponding Author

Aamer Imdad, MBBS, MPH

Assistant Professor of Pediatrics

Division of Pediatric Gastroenterology, Hepatology and Nutrition

SUNY Upstate Medical University, Syracuse, NY

725 Irving Street, Syracuse, NY 13210

Email: imdada@upstate.edu

Phone: 315-464-8444

Fax: 315-464-8443

Melissa François

SUNY Upstate Medical University

Syracuse, New York, USA

FrancoMe@upstate.edu

Fanny Chen

SUNY Upstate Medical University

Syracuse, New York, USA

ChenF@upstate.edu

Abigail Smith

SUNY Upstate Medical University

Syracuse, New York, USA

SmithAb@upstate.edu

Olivia Tsistinas

For peer review only

For peer review only - http://bmjopen.bmj.com/site/about/guidelines.xhtml

SUNY Upstate Medical University
Syracuse, New York, USA
TsistiJO@upstate.edu

Emily Tanner-Smith
University of Oregon
etanners@uoregon.edu
Eugene, Oregon, USA

Jai Das
Aga Khan University
Karachi, Sindh, Pakistan
Jai.das@aku.edu

For peer review only

For peer review only - http://bmjopen.bmj.com/site/about/guidelines.xhtml

Zulfiqar Ahmed Bhutta
Institute for Global Health & Development, The Aga Khan University
Karachi, Sindh, Pakistan
zulfiqar.bhutta@aku.edu

Word Count: 3265

For peer review only

For peer review only - http://bmjopen.bmj.com/site/about/guidelines.xhtml

ABSTRACT

Introduction

The current standard of care for children with severe acute malnutrition involves using ready-to-use therapeutic food (RUTF) to promote growth; however, the precise formulation to achieve optimal recovery remains unclear. Emerging research suggests that alternative RUTF formulations may be more effective in correcting severe acute malnutrition (SAM) related complications such as anemia and iron deficiency. This systematic review commissioned by the World Health Organization (WHO) aims to synthesize the most recent research on the iron content in RUTF and related products in the community-based treatment of uncomplicated severe malnutrition in children aged six months and older.

Methods and Analysis

We will search multiple electronic databases. We will include randomized controls trials and non-randomized studies with a control arm. The intervention group will be infants who received RUTF treatments other than the current recommended guidelines set forth by the WHO. The comparison group is children receiving RUTF containing iron at the current WHO recommended level of 1.9 mg/100 kcal (10-14 mg/100 g). The primary outcomes of interest include blood hemoglobin concentration, any anemia, severe anemia, iron deficiency anemia, recovery from severe acute malnutrition, and any adverse outcomes. We will use meta-analysis to pool findings if sufficient homogeneity exists among included studies. The risk of bias in studies will be evaluated using the Cochrane risk of bias-2 (ROB 2.0) and Cochrane ROBINS-I tools. We will use the Grading of Recommendations Assessment, Development, and Evaluation (GRADE) approach to examine the overall certainty of evidence.

Ethics and Dissemination: This is a systematic review and will not involve direct contact with human subjects. The findings of this review will be published in a peer-review journal and will guide the WHO's recommendation on the optimal iron content in ready-to-use therapeutic foods for the treatment of severe acute malnutrition in children aged 6-59 months.

Key Words: Iron, ready-to-use therapeutic food, wasting, malnutrition, growth failure

For peer review only - http://bmjopen.bmj.com/site/about/guidelines.xhtml

ARTICLE SUMMARY: STRENGTHS AND LIMITATIONS OF THIS STUDY

- This systematic review commissioned by the WHO aims to synthesize the most recent research on iron content is ready to use therapeutic foods and related products in the community-based treatment of uncomplicated severe malnutrition in children aged six months and older.
- We will search several databases for relevant literature and include randomized and non-randomized studies.
- We will assess risk-of-bias for each outcome and use the Grading of Recommendations Assessment, Development, and Evaluation to assess the overall quality of evidence.
- We will assess the following outcomes: blood hemoglobin concentration, any anemia, severe anemia, iron deficiency anemia, recovery rates, all-cause mortality, adverse events, growth outcome, and serum levels of micronutrients.
- We will conduct meta-analyses if data are available from more than one study and there is clinical and methodological homogeneity in the included studies.
- All the included studies may not report the data for all the outcomes mentioned in this review which will be a limitation of the study.

For peer review only

For peer review only - http://bmjopen.bmj.com/site/about/guidelines.xhtml

Full manuscript

BACKGROUND

The World Health Organization (WHO) estimates over 45 million children under the age of five worldwide suffered from wasting (low weight for height) in 2020 alone[1]. Children with severe acute malnutrition (SAM) have about 6 fold increased mortality risk compared to well-nourished children[2]. In addition to the risk of mortality, children with SAM are at increased risk of morbidities such as diarrhea, pneumonia, measles, and micronutrient deficiencies, and long-term neurodevelopmental delay[2-4]. Nutritional rehabilitation of children with SAM is a key intervention that helps treat morbidity and prevent mortality[3,5,6]. The current standard of care for children with uncomplicated severe acute malnutrition involves using ready-to-use therapeutic food (RUTF) to promote growth[7]; however, the precise formulation to achieve optimal recovery remains unclear[6,8]. There are ongoing efforts to improve RUTF to optimize the composition and balance of macronutrients and micronutrients[9-11].

Severe acute malnutrition involves deficits of several key macronutrients and micronutrients, with studies showing higher rates of anemia in children suffering from malnutrition [12-14]. The prevalence of anemia in severely acutely malnourished children ranges from 40% to 90%, and

For peer review only - http://bmjopen.bmj.com/site/about/guidelines.xhtml

about half of the anemia is attributed to iron deficiency[15]. Iron deficiency in severely malnourished children could be due to low intake, increased losses, higher demand, and poor absorption. Iron is essential for adequate catch-up growth and neurological development in children with severe acute malnutrition[12,16,17]. Moreover, recent studies suggest alternative RUTF formulations may be more effective in correcting anemia and iron deficiency in children with severe acute malnutrition compared to current standard RUTF formulations [12,18,19]. In addition to the need for sufficient iron intake to treat iron deficiency, it remains unclear whether improving the iron status among the undernourished children increases the risk of complications, including the risk of morbidities such as diarrhea, malaria, and undesired changes in the microbiome[20-22]. This aspect is especially of concern in malaria-endemic regions in Africa because nearly a third (27%) of all children affected by wasting worldwide reside in Africa[22]. Due to the critical role of RUTF in the medical management of children with severe acute malnutrition, more information is needed to generate formulations with optimal iron levels for treatment of anemia and optimal growth and development in children suffering from malnutrition [16-18]. A number of recent studies have been completed to assess the optimal dose of iron in RUTF in the community management of SAM, and there is no existing systematic review on this topic[12,19,23]. Furthermore, WHO has started to synthesize the evidence to update the current guidelines for treating wasting in children. Therefore, this WHO commissioned review aims to synthesize the most recent research on the iron content in RUTF and related products in treating severe malnutrition in children 6-59 months of age.

OBJECTIVE

Primary Objective*:* In children aged 6 months or older with uncomplicated severe acute malnutrition being treated in the community settings, does increased iron dose in RUTF compared with the WHO recommendation for iron fortification of RUTF improve outcomes such as blood hemoglobin concentration, recovery from iron-deficiency anemia, etc.?

Secondary Objective*:* In children aged 6 months or older with uncomplicated severe acute malnutrition being treated in the community settings, what mechanisms other than increased dose of iron in RUTF (such as type, composition, zinc, phytate content etc.) can help increase the bioavailability of iron and improve the recovery from iron deficiency anemia?

For peer review only - http://bmjopen.bmj.com/site/about/guidelines.xhtml

METHODS AND ANALYSIS

We will follow the standard guidelines of the Cochrane Collaboration to conduct the systematic review. We registered a detailed protocol on PROSPERO (ID=CRD42021278006) and would report the systematic review findings according to PRISMA 2020 guidelines[24].

Study type: We will consider randomized trials, and that may include individual and cluster randomized trials. We will also consider non-randomized studies if there are not enough randomized studies. The data will be analyzed separately for randomized and non-randomized studies. We will include the following non-randomized studies: Non-randomized controlled clinical trials and controlled before and after studies (CBAs). Case-control studies, interrupted time series, program evaluations, case reports, case series, and commentaries will be excluded. The definitions of eligible studies design and consideration for inclusion in this review are available in table 1.

Population

The population of interest is children aged 6 months or older with severe acute malnutrition managed in outpatient settings. The definition of severe acute malnutrition will be based on weight for height z scores (< -3 SD for WHO growth standards) or mid-upper arm circumference (< 115 mm) or presence of bilateral edema[25]. We will consider studies that included children who were previously admitted to the hospital and are now being rehabilitated in community settings with the help of RUTF. We will exclude studies of children with complicated SAM who are admitted to the hospital, as those participants might have complications such as pneumonia or severe diarrhea. We will exclude studies done specifically on participants who have chronic diseases, genetic disorders, or congenital anomalies.

Intervention

The intervention of interest is the dose of iron in RUTF other than in the standard RUTF for the treatment of severe acute malnutrition in community settings. We will include studies of iron-fortified RUTF and exclude those studies where iron was given separately as supplements, such as tablets, syrup, multiple micronutrient powder, etc. We will include studies where RUTF is given as the main intervention, and it met total daily requirements or was given as a

For peer review only - http://bmjopen.bmj.com/site/about/guidelines.xhtml

supplementary intervention to the usual diet for children with severe acute malnutrition recovering at home. WHO recommends that a standard RUTF should have at least 1.9 mg/100 kcal (or 10-14 mg/100 g) of iron to treat severe acute malnutrition in children 6-59 months of age[25]. We will include studies irrespective of the type of RUTF used, i.e., standard RUTF vs. low milk-based vs. non-milk-based vs. locally prepared. We will exclude studies where RUTF was given to healthy children to prevent severe acute malnutrition; treatment of moderate acute malnutrition; as part of complementary feeding.

Comparison

For the primary objective of the review, the comparison group will be children receiving RUTF containing iron at the current WHO recommended level of 1.9 mg/100 kcal (10-14 mg/100 g) to treat severe acute malnutrition. For the secondary objective of the review, we will consider studies where different types/doses/techniques of RUTF are studied to improve the bioavailability of iron even though the iron content may be the same in all kinds of RUTF given to children. We will exclude studies where the comparison group did not receive any RUTF.

OUTCOMES

Primary Outcomes

- Blood hemoglobin concentration (continuous outcome)
- Any Anemia (dichotomous outcome, as defined by authors)
- Severe anemia (dichotomous outcome, as defined by authors)
- Iron deficiency anemia (dichotomous outcome as defined by authors)
- Recovery from severe acute malnutrition (dichotomous outcome, as defined by authors)
- Any Adverse events (Dichotomous outcome)

Secondary Outcomes

- Serum ferritin level (continuous outcome)
- Serum zinc level (continuous outcome)
- Serum copper level (continuous outcome)
- Serum iron level (continuous outcome)
- Adverse events Malaria: (as defined by authors) (dichotomous outcome)
- Relapse (dichotomous outcome)
- All-cause mortality (dichotomous outcome)

For peer review only - http://bmjopen.bmj.com/site/about/guidelines.xhtml

- Admission to an inpatient facility. (dichotomous outcome)
- Withdrawal from the trial (Dichotomous outcome)
- Constipation (<3 bowel movements per week) (dichotomous outcome)
- Adverse events Diarrhea: (>3 loose stools per day) (dichotomous outcome)
- Adverse events: Pneumonia (as defined by authors) (dichotomous outcome)
- Weight for age (kg or Z scores)
- Height for age (cm or Z scores)
- Weight for height Z score
- Microbiome outcomes: Alpha diversity and Beta Diversity
- Neurodevelopmental outcomes (continuous outcome) at 1 year and the longest follow-up.

  The term neurodevelopment is a composite term that refers to cognitive, neurological, and/or sensory outcomes. This assessment may include intellectual disability as measured on the Mental Developmental Index of the Bayley Scales of Infant Development, gross motor delay measured on the Gross Motor Function Classification System, and so on.

We will consider the data for all the above outcomes at the longest follow-up.

LITERATURE SEARCH

We will conduct systematic electronic queries using key terms in several databases, including PubMed, EMBASE, the Cochrane Central Register for Controlled Trials, Web of Science, CINHAL,
Scopus, LILACS, and WHO Global Index Medicus. No search restrictions will be used to exclude studies based on the outcome, publication year, publication status, or language. The references of formerly published reviews and recently published studies will be examined for potential inclusion. We will also use the citation tracking function of the included studies in the PubMed to look for any eligible studies. In addition to the above resources, ClinicalTrials.gov will be used to identify studies currently underway. We will also use the clinical trial registration number to find all the relevant studies published from a particular trial. Finally, we will search the grey literature and search the websites of pertinent international agencies such as WHO (including WHO's Reproductive Health Library, electronic Library of Evidence for Nutrition

For peer review only - http://bmjopen.bmj.com/site/about/guidelines.xhtml

Actions and Global database on the Implementation of Nutrition Action), UNICEF, Global Alliance for Improved Nutrition, International Food Policy Research Institute, International Initiative for Impact Evaluation (3ie), Nutrition International, World Bank, USAID and affiliates (e.g., FANTA, SPRING), and the World Food Program. We will also search the abstracts presented in major pediatric conferences such as Pediatric academic society meeting. Proposed search strategies for different electronic databases are shown in Appendix 1.

DATA EXTRACTION AND SYNTHESIS

Selection of studies

Searches from all the databases will be combined in bibliographic software (EndNote), and duplicates will be removed. Two authors will use a three-phase approach in duplicate to screen studies identified from the search for eligibility. In the first phase, authors will screen the titles and abstracts to identify potentially eligible ones; studies selected during this initial phase will then go on to a full-text review as the second phase. Lastly, studies determined to be eligible subsequent to full-text review will undergo data extraction during the third and final phase. The software Covidence14[26] will be used to assist the screening process. Two authors will independently extract data from screened studies and compare their findings. Potential conflicts will be resolved through discussion, and the senior author on the team may assist as needed to resolve any conflicts. If a study is only available in abstract form, we will write to authors to obtain details on methods and results. If we cannot get full details of the study methods available in the abstract, we will decide about inclusion based on details available in the abstract. If a study is available in a language other than English, we will attempt to complete the translation using local resources. If a study was published in more than one report (multiple publications), we will count those multiple reports as a single study and extract information from all the available reports as needed.

Data extraction

A data extraction form was designed to support the review and data extraction process (supplementary document 1). Two authors will independently extract the data and compare their findings. Any conflict will be resolved by discussion and with the help of the senior author on the team if needed. The following information will be extracted for each study where available: study design (randomized control trial, quasi-randomized experimental design or cohort study, CBA),

For peer review only - http://bmjopen.bmj.com/site/about/guidelines.xhtml

study site (country/region), study year, study type, intervention (dose of iron and dose of RUTF, duration, frequency, and composition), exposures, comparison, outcomes, whether the results were adjusted for confounders, and risk of bias. To avoid reviewer bias, we are deciding a priori the order of preference for extracting outcomes when data might be available in several formats. For randomized studies, we will prefer data that require the least manipulation by authors or inference by reviewers. We will extract the raw values (for example, means and standard deviations) rather than calculated effect sizes (e.g., Cohen's d). For mortality data, we will give preference to denominators in the following order: number with the definite outcome known (or imputed as described below), number randomized, and child-years. For morbidity outcomes to which both survivors and non-survivors may have contributed data (for example, the incidence of pneumonia), we will give preference to child years, number with the definite outcome known, and number randomized.

Studies with missing data

We will document attrition during data extraction. If data are missing for some cases, or if reasons for dropout are not reported, we will contact the trial authors to request the full data. If the authors report the missing data and report results using imputations for the missing data, we will use the latter. If a study does not report the standard deviation (SD) for the continuous outcome and the SD cannot be calculated from the reported data (such as standard error, confidence interval, p-value), we will write to the authors to request the data for SD. If the SD data are not available from authors, we will use SD from a similar study that has a similar study population. We will prefer to use the final values of a continuous outcome for a given follow-up. If the final values are not available but the difference between the end and the start of the study, we will write to authors to request the final values. If the final values are not available, we will use the difference or rate of change. We will use the data based on intention to treat analysis. If the data for intention to treat analysis is not given in the study, we will create our own intention to treat analysis for participants with known outcomes. If there is significant attrition between the randomized participants vs. participants completing the study, we will include such a study but will investigate further with sensitivity analysis.

Assessment of risk of bias in included studies

The risk of bias in studies will be evaluated using the Cochrane risk of bias (ROB 2.0) and Cochrane ROBINS-I for non-randomized and observational studies [27,28]. Two review authors will

For peer review only - http://bmjopen.bmj.com/site/about/guidelines.xhtml

independently assess and agree on the risk of bias for the individual studies for an outcome. The discussion will resolve any disagreements, and if no agreement can be made, the senior review author will be consulted. We will assess the effect of assignment to intervention (the intention-to-treat effect) for randomized trials by addressing five domains of signaling questions in ROB-2 including: bias arising from the randomization process, bias due to deviations from intended interventions, bias due to missing outcome data, bias in the measurement of the outcome and bias in the selection of the reported result. Each domain will receive a ranking of one of the following: low risk of bias, some concerns of bias, or high risk of bias. We will include quotes from the study for each signaling question as evidence for our ranking decision. The overall risk of bias will be determined based on the lowest ranking for individual domains. For example, if only one domain is ranked 'some concerns,' then the overall risk of bias will be 'some concerns'.

Data Synthesis

The findings from the systematic review will be reported both qualitatively and quantitatively. A narrative synthesis will be used to report the characteristics of all included studies. We will also narratively describe the data in a table for the secondary objective of this review (table 2). A random-effects meta-analysis will be employed when at least two studies possess sufficient clinical and methodological uniformity for synthesis for the primary objective of the review. We will use the generic inverse method to pool the studies in the meta-analysis. The software RevMan will be utilized for statistical analysis[29]. Dichotomous outcomes will be assessed using relative risk effect sizes and presented with 95% confidence intervals. In the case of morbidity outcomes, we will combine all available data whenever possible if outcomes are measured in different ways. For example, we will include all types of diarrhea (mild, moderate, and severe) as a dichotomous value (yes/no) if participants had greater than 3 instances of loose stools per day. We will include the occurrence of anemia, iron deficiency anemia, and pneumonia throughout the study as dichotomous values (yes/no). We will pool the data for continuous outcomes to obtain an average mean difference and report it with its 95% CI. If data are reported in different units (e.g., few studies report weight in kg and the others report in Z scores), we will use a standardized mean difference effect size and report it with its 95% CI. We will pool the data separately from randomized and observational studies. If an observational study reports both adjusted and unadjusted values, we will use the adjusted values for meta-analysis.

For peer review only - http://bmjopen.bmj.com/site/about/guidelines.xhtml

We will consider the following pairs of comparisons:

High iron content in RUTF vs. WHO standard iron content in RUTF

The Grading of Recommendations Assessment, Development, and Evaluation (GRADE) approach will be used to evaluate overall evidence quality using the software GRADEpro[30]. The GRADE approach is a comprehensive framework used to assess the overall quality of evidence for an outcome using characteristics such as study design, heterogeneity, directness of evidence, risk of bias, publication bias, and precision of effect estimates[31]. The results of the GRADE assessment will be included in a summary of the findings table. The table will contain quality ratings characterized as very low (we have very little confidence in the effect estimate), low (we have limited confidence in the effect estimate), moderate (we have moderate confidence in the effect estimate; the true effect is likely close to the estimate of the effect), or high based (we have high confidence that the true effect lies close to that of the estimate of the effect) on the primary outcomes of each study (Table 3). The study will be started on January 18, 2022, and hope to be completed by March 18, 2022.

Patient and public involvement

No patient or public involvement was considered in the preparation of this protocol.

UNIT OF ANALYSIS ISSUES

Multiple-arm trial

Studies with multiple treatment arms will be included if eligible. For multiple-arm trials, we will include data so that the only difference between the groups is the use of iron in RUTF.

Cluster trials

Cluster assignment trials will be analyzed together with individually randomized trials. We will use the cluster adjusted values; if the trial results are not adjusted for cluster design, we will adjust the result by methods given in the Cochrane[32] handbook for systematic reviews.

Assessment of heterogeneity

For peer review only - http://bmjopen.bmj.com/site/about/guidelines.xhtml

Statistical heterogeneity of effect sizes within any given meta-analysis will be assessed using the χ^2, I^2, and tau statistics. We will assess statistical heterogeneity by visual inspection of forest plots, performing

the χ^2test (assessing the p-value), and calculating the Tau^2 and I^2 statistics. Statistical heterogeneity will be considered significant if the p-value is less than 0.10, I^2 value exceeds 50%, and the examination of forest plots shows substantial variability in the effect of the intervention. We will perform subgroup analysis to determine the reasons for any identified statistical heterogeneity.

Assessment of reporting bias

Small study and publication bias will be assessed using funnel plots and regression tests for funnel plot asymmetry when a meta-analysis includes at least ten studies.

Subgroup analyses

- Settings: Country: Low-income country vs. middle-income country vs. high-income country
- Type of RUTF: Standard RUTF vs. Non-standard
- Role of RUTF: RUTF as main treatment vs. RUTF as a supplementary intervention
- Type of participants: Studies that included children with HIV vs. Studies with children without HIV.
- Age: <24 months vs. 24-59 months vs. > 59 months
- Hospitalization: Children hospitalized (due to medical complication) prior to starting RUTF vs. Children not hospitalized prior to starting RUTF
- Iron compound (formulation/chemical compound and amount)
- Dose: The intervention groups with a dose of iron > standard WHO RUTF Vs. Intervention group receiving dose lower the Standard WHO RUTF
- Anemia Status: Children with anemia at the baseline vs. without anemia at the baseline
- Time of follow up: 1 month vs. 3 months vs. 6 months follow up and the longest follow up

Sensitivity analyses:

-Studies with a high risk of bias

-Random vs. fixed effect model

Amendments

For peer review only - http://bmjopen.bmj.com/site/about/guidelines.xhtml

For peer review only

We will do the literature searches, screening of titles, selection of studies, data extraction, and analysis according to the aforementioned plan described in this protocol. If we do any additional analysis or change any of the a priori strategies, we will clearly describe that in the methods section.

Consent for publication

The authors give consent for the publication of the review.

Competing interests

None declared.

Availability of data and materials

We will provide data available for review by the editors and peer reviewers and provide raw data for the general public on request.

Funding

This work is funded by World Health Organization grant no 202712614.

Role of Funding source

WHO provided technical support for this work and guided the development of PICO (Population, Intervention, Comparison, Outcome) question for this review.

Acknowledgments

We are very thankful to Allison Daniel, Jaden Bendabenda, Lisa Rogers, and Maria De Las Nieves Garcia Casal for their input to improve this protocol.

Authors' contributions

AI and MF wrote the first draft of the manuscript. AS and OT developed the search strategy. ET-S

provided the support for methodology. ZAB, FC and JD provided the support for the content being evaluated in the review. All authors edited and reviewed the manuscript. AI is the guarantor of the review.

For peer review only - http://bmjopen.bmj.com/site/about/guidelines.xhtml

For peer review only

ETHICS AND DISSEMINATION

This is a systematic review and will not involve direct contact with human subjects. The findings of this review will be published in a peer-review journal and will guide the WHO's recommendation on the optimal iron content in ready-to-use therapeutic foods for the treatment of severe acute malnutrition in children aged 6-59 months.

For peer review only

For peer review only - http://bmjopen.bmj.com/site/about/guidelines.xhtml

REFERENCES

1. United Nations Children's Fund (UNICEF), World Health Organization, International Bank for Reconstruction and Development/The World Bank. *Levels and trends in child malnutrition: key findings of the 2021 edition of the joint child malnutrition estimates*. Geneva: World Health Organization; 2021.
2. Schroeder DG, Brown KH. Nutritional status as a predictor of child survival: summarizing the association and quantifying its global impact. *Bull World Health Organ.* 1994;72(4):569-579.
3. Dipasquale V, Cucinotta U, Romano C. Acute Malnutrition in Children: Pathophysiology, Clinical Effects and Treatment. *Nutrients.* 2020;12(8).
4. Mwene-Batu P, Bisimwa G, Baguma M, et al. Long-term effects of severe acute malnutrition during childhood on adult cognitive, academic and behavioural development in African fragile countries: The Lwiro cohort study in Democratic Republic of the Congo. *PLoS One.* 2020;15(12):e0244486.
5. Woeltje MM, Evanoff AB, Helmink BA, et al. Community-based management of acute malnutrition for infants under 6 months of age is safe and effective: analysis of operational data. *Public Health Nutr.* 2021:1-10.
6. Das JK, Salam RA, Saeed M, Kazmi FA, Bhutta ZA. Effectiveness of Interventions for Managing Acute Malnutrition in Children under Five Years of Age in Low-Income and Middle-Income Countries: A Systematic Review and Meta-Analysis. *Nutrients.* 2020;12(1).
7. WHO W, UNSCN, UNICEF. Community-based management of severe acute malnutrition. A joint statement by the World Health Organization, World Food Programme, United Nations Standing Committee on Nutrition, United Nations Children's Fund. Geneva, World Health Organization. 2007.
8. Weisstaub G, Medina M, Pizarro F, Araya M. Copper, iron, and zinc status in children with moderate and severe acute malnutrition recovered following WHO protocols. *Biol Trace Elem Res.* 2008;124(1):1-11.
9. Potani I, Spiegel-Feld C, Brixi G, et al. Ready-to-Use Therapeutic Food (RUTF) Containing Low or No Dairy Compared to Standard RUTF for Children with Severe Acute Malnutrition: A Systematic Review and Meta-Analysis. *Adv Nutr.* 2021;12(5):1930-1943.
10. Schoonees A, Lombard MJ, Musekiwa A, Nel E, Volmink J. Ready-to-use therapeutic food (RUTF) for home-based nutritional rehabilitation of severe acute malnutrition in children from six months to five years of age. *Cochrane Database Syst Rev.* 2019;5:CD009000.
11. Shokry E, Sadiq K, Soofi S, et al. Impact of Treatment with RUTF on Plasma Lipid Profiles of Severely Malnourished Pakistani Children. *Nutrients.* 2020;12(7).
12. Akomo P, Bahwere P, Murakami H, et al. Soya, maize and sorghum ready-to-use therapeutic foods are more effective in correcting anaemia and iron deficiency than the standard ready-to-use therapeutic food: randomized controlled trial. *BMC Public Health.* 2019;19(1):806.
13. Savadogo LGB, Zoetaba I, Ilboudo B, Kinda M, Donnen P. Impact of anemia on mortality and nutritional recovery among hospitalized severely malnourished children in Burkina Faso. *Open Journal of Pediatrics.* 2014;2014.

For peer review only - http://bmjopen.bmj.com/site/about/guidelines.xhtml

14. Thakur N, Chandra J, Pemde H, Singh V. Anemia in severe acute malnutrition. *Nutrition.* 2014;30(4):440-442.
15. Kangas ST, Salpeteur C, Nikiema V, et al. Vitamin A and iron status of children before and after treatment of uncomplicated severe acute malnutrition. *Clin Nutr.* 2020;39(11):3512-3519.
16. Babirekere-Iriso E, Mortensen CG, Mupere E, et al. Changes in whole-blood PUFA and their predictors during recovery from severe acute malnutrition. *Br J Nutr.* 2016;115(10):1730-1739.
17. Jones KD, Ali R, Khasira MA, et al. Ready-to-use therapeutic food with elevated n-3 polyunsaturated fatty acid content, with or without fish oil, to treat severe acute malnutrition: a randomized controlled trial. *BMC Med.* 2015;13:93.
18. Lopriore C, Guidoum Y, Briend A, Branca F. Spread fortified with vitamins and minerals induces catch-up growth and eradicates severe anemia in stunted refugee children aged 3-6 y. *Am J Clin Nutr.* 2004;80(4):973-981.
19. Bahwere P, Balaluka B, Wells JC, et al. Cereals and pulse-based ready-to-use therapeutic food as an alternative to the standard milk- and peanut paste-based formulation for treating severe acute malnutrition: a noninferiority, individually randomized controlled efficacy clinical trial. *Am J Clin Nutr.* 2016;103(4):1145-1161.
20. Soofi S, Cousens S, Iqbal SP, et al. Effect of provision of daily zinc and iron with several micronutrients on growth and morbidity among young children in Pakistan: a cluster-randomised trial. *Lancet.* 2013;382(9886):29-40.
21. Paganini D, Zimmermann MB. The effects of iron fortification and supplementation on the gut microbiome and diarrhea in infants and children: a review. *Am J Clin Nutr.* 2017;106(Suppl 6):1688S-1693S.
22. Sazawal S, Black RE, Ramsan M, et al. Effects of routine prophylactic supplementation with iron and folic acid on admission to hospital and mortality in preschool children in a high malaria transmission setting: community-based, randomised, placebo-controlled trial. *Lancet.* 2006;367(9505):133-143.
23. Irena AH, Bahwere P, Owino VO, et al. Comparison of the effectiveness of a milk-free soy-maize-sorghum-based ready-to-use therapeutic food to standard ready-to-use therapeutic food with 25% milk in nutrition management of severely acutely malnourished Zambian children: an equivalence non-blinded cluster randomised controlled trial. *Matern Child Nutr.* 2015;11 Suppl 4:105-119.
24. Page MJ, McKenzie JE, Bossuyt PM, et al. The PRISMA 2020 statement: an updated guideline for reporting systematic reviews. *BMJ.* 2021;372:n71.
25. WHO. Management of severe malnutrition : a manual for physicians and other senior health workers. 1999.
26. Covidence systematic review software vhi, Melbourne, Australia. Available: www.covidence.org.
27. Sterne JA, Hernan MA, Reeves BC, et al. ROBINS-I: a tool for assessing risk of bias in non-randomised studies of interventions. *BMJ.* 2016;355:i4919.
28. Sterne JAC, Savovic J, Page MJ, et al. RoB 2: a revised tool for assessing risk of bias in randomised trials. *BMJ.* 2019;366:l4898.

For peer review only - http://bmjopen.bmj.com/site/about/guidelines.xhtml

29. Review Manager (RevMan) [Computer program]. Version 5.3. Copenhagen: The Nordic Cochrane Centre TCC. Version 5.3. Copenhagen: The Nordic Cochrane Centre, The Cochrane Collaboration. Published 2014. Accessed. .
30. GRADEpro GDT: GRADEpro Guideline Development Tool [Software]. McMaster University, 2020 (developed by Evidence Prime, Inc.). Available from gradepro.org.
31. Guyatt G, Oxman AD, Akl EA, et al. GRADE guidelines: 1. Introduction-GRADE evidence profiles and summary of findings tables. *J Clin Epidemiol.* 2011;64(4):383-394.
32. Higgins JPT TJ CJ, Cumpston M. Cochrane handbook for systematic reviews of interventions version 6.2, 2021. https://training.cochrane.org/handbook/PDF/v6.2.
33. (EPOC) CEPaOoC. What study designs can be considered for inclusion in an EPOC review and what should they be called? Avaialble at https://epoc.cochrane.org/sites/epoc.cochrane.org/files/public/uploads/EPOC%20Study%20Designs%20About.pdf. July 2021.

For peer review only

For peer review only - http://bmjopen.bmj.com/site/about/guidelines.xhtml

Table 1: The definitions of Eligible Study Designs

Study Designs	Definition	Notes
Randomized controlled trial (RCT) OR randomized trial	An experimental study in which people are allocated to different interventions using methods that are random.”	We will include both individual and cluster randomized trials. In individual randomized trials, the randomized is done at the individual levels, while in cluster randomized trials, the randomized is based on cluster or groups of individuals. We will also consider factorial design trials where multiple interventions are studied in the same trial.
Non-randomised controlled trial (NRCT) OR non-randomised trial	“An experimental study in which people are allocated to different interventions using methods that are not random.”	We will avoid using the term quasi experimental studies as it means differently by different authors. We will exclude the experimental studies where there was no control group.
Controlled before-after study (CBA)	“A study in which observations are made before and after the implementation of an intervention, both in a group that receives the intervention and in a control group that does not.”	We will require two minimum criteria for the inclusion of CBAs.<br>-Data Collection: We will include CBAs if the data for the intervention and control groups were collected prospectively in the same time frame<br>-Choice of Control: We will include CBAs that include a control at a second site to avoid contamination of the intervention to the control group if the settings and populations are the same for the intervention and control groups.

Footnotes: The definitions of study designs were adopted from The Cochrane Effective Practice and Organization of Care (EPOC) group[33]

For peer review only

Table 2: Sample table to describe the studies that address delivery mechanism to increase the bioavailability of iron via RUTF in children 6 months and older with severe acute malnutrition

Study	Study Participants	Formulations used	Comparison group	Outcomes	Notes

For peer review only

Table 3: The Criteria for the Grading of Recommendations Assessment, Development, and Evaluation (GRADE) approach to examine the overall certainty of evidence[31]

<table>
<tr><td>Study Design</td><td>Quality of Evidence</td><td>Lower Certainty Score If</td><td>Higher Certainty Score If</td></tr>
<tr><td rowspan="2">Randomized trial</td><td>High</td><td rowspan="3">Risk of Bias
-1 Serious
-2 Very serious

Inconsistency
-1 Serious
-2 Very serious

Indirectness
-1 Serious
-2 Very serious

Imprecision</td><td rowspan="3">Large effect
+1 Large
+2 Very large

Dose-response
+1 evidence of a gradient

All plausible confounding would:
+1 Reduce a demonstrated effect

+1 Suggest a spurious effect</td></tr>
<tr><td>Moderate</td></tr>
<tr><td>Observational study</td><td>Low</td></tr>
</table>

For peer review only - http://bmjopen.bmj.com/site/about/guidelines.xhtml

		-1 Serious	when results show no effect
	Very Low	-2 Very serious<br><br>Publication Bias<br>-1 Likely<br>-2 Very likely	

For peer review only

Appendix 1: Search strategies for different electronic databases

For peer review only - http://bmjopen.bmj.com/site/about/guidelines.xhtml

PubMed

("Iron"[MeSH Terms] OR "iron, dietary"[MeSH Terms] OR "Iron"[Title/Abstract] OR "56Fe"[Title/Abstract] OR "Fe"[Title/Abstract] OR "ferro"[Title/Abstract] OR "ferrum"[Title/Abstract]) AND ("food, formulated"[MeSH Terms] OR "food, fortified"[MeSH Terms] OR "RUTF"[Title/Abstract] OR "RUTFs"[Title/Abstract] OR "ready to use therapeutic food*"[Title/Abstract] OR "ready to use therapeutic feed*"[Title/Abstract] OR "therapeutic food*"[Title/Abstract] OR "therapeutic diet*"[Title/Abstract] OR "enriched food*"[Title/Abstract] OR "enriched diet*"[Title/Abstract] OR "fortified food*"[Title/Abstract] OR "fortified diet*"[Title/Abstract] OR "supplemented food*"[Title/Abstract] OR "supplemental food*"[Title/Abstract] OR "supplementary food*"[Title/Abstract] OR "supplement food*"[Title/Abstract] OR "supplemented diet*"[Title/Abstract] OR "supplemental diet*"[Title/Abstract] OR "supplementary diet*"[Title/Abstract] OR "supplement diet*"[Title/Abstract] OR "RUSF"[Title/Abstract] OR "RUSFs"[Title/Abstract] OR "ready to use supplemental food*"[Title/Abstract] OR "ready to use supplementary food*"[Title/Abstract] OR "lipid based nutrient supplement*"[Title/Abstract] OR "LNS"[Title/Abstract]) AND ("Severe Acute Malnutrition"[MeSH Terms] OR "Malnutrition"[MeSH Terms] OR "Cachexia"[MeSH Terms] OR "Wasting Syndrome"[MeSH Terms] OR "Severe Acute Malnutrition"[Title/Abstract] OR "SAM"[Title/Abstract] OR "Malnutrition"[Title/Abstract] OR "malnourish*"[Title/Abstract] OR "Cachexia"[Title/Abstract] OR "nutritional deficien*"[Title/Abstract] OR "undernutrition"[Title/Abstract] OR "deficient nutrition"[Title/Abstract] OR "undernourish*"[Title/Abstract] OR "wasting"[Title/Abstract] OR "wasted"[Title/Abstract]) AND ("Infant"[MeSH Terms] OR "Child"[MeSH Terms] OR "infant*"[Title/Abstract] OR "infancy"[Title/Abstract] OR "baby"[Title/Abstract] OR "babies"[Title/Abstract] OR "newborn*"[Title/Abstract] OR "neonat*"[Title/Abstract] OR "neo nat*"[Title/Abstract] OR "child*"[Title/Abstract] OR "toddler*"[Title/Abstract] OR "youth"[Title/Abstract] OR "juvenile*"[Title/Abstract] OR "girl*"[Title/Abstract] OR "boy"[Title/Abstract] OR "boys"[Title/Abstract] OR "preschool*"[Title/Abstract] OR "pre school*"[Title/Abstract])

CINAHL

S1 (MH "Infant+")

S2 (MH "Child+")

S3 TI (infant* OR infancy OR baby OR babies OR newborn* OR neonat* OR "neo nat*" OR child* OR toddler* OR youth OR juvenile* OR girl* OR boy OR boys OR preschool* OR "preschool*")

S4 AB (infant* OR infancy OR baby OR babies OR newborn* OR neonat* OR "neo nat*" OR child* OR toddler* OR youth OR juvenile* OR girl* OR boy OR boys OR preschool* OR "preschool*")

S5 S1 OR S2 OR S3 OR S4

S6 (MH "Iron")

S7 TI (iron OR 56Fe OR Fe Or ferro OR ferrum)

For peer review only - http://bmjopen.bmj.com/site/about/guidelines.xhtml

S8 AB (iron OR 56Fe OR Fe Or Ferro OR ferrum)

S9 S6 OR S7 OR S8

S10 (MH "Food, Formulated")

S11 (MH "Food, Fortified")

S12 TI (RUTF OR RUTFs OR "ready to use therapeutic food*" OR "ready to use therapeutic feed*" OR RUSF OR RUSFs OR "ready to use supplemental food*" OR "ready to use supplementary food*" OR "lipid-based nutrient supplement*" OR LNS)

S13 AB (RUTF OR RUTFs OR "ready to use therapeutic food*" OR "ready to use therapeutic feed*" OR RUSF OR RUSFs OR "ready to use supplemental food*" OR "ready to use supplementary food*" OR "lipid-based nutrient supplement*" OR LNS)

S14 TI((enriched OR fortified OR supplement* OR therapeutic) W1 (food* OR diet*))

S15 AB((enriched OR fortified OR supplement* OR therapeutic) W1 (food* OR diet*))

S16 S10 OR S11 OR S12 OR S13 OR S14 OR S15

S17 (MH "Cachexia")

S18 (MH "Malnutrition")

S19 (MH "Wasting Syndrome")

S20 TI ("Severe Acute Malnutrition" OR SAM OR Malnutrition OR malnourish* OR Cachexia OR undernutrition OR undernourish* OR wasting OR wasted)

S21 AB ("Severe Acute Malnutrition" OR SAM OR Malnutrition OR malnourish* OR Cachexia OR undernutrition OR undernourish* OR wasting OR wasted)

S22 TI (nutrition* N1 deficien*)

S23 AB (nutrition* N1 deficien*)

S24 S17 OR S18 OR S19 OR S20 OR S21 OR S22 OR S23

S25 S5 AND S9 AND S16 AND S24

S26 S25 Exclude MEDLINE records

Embase

#1 'iron'/exp OR 'iron intake'/exp

#2 iron:ti,ab OR 56fe:ti,ab OR fe:ti,ab OR ferro:ti,ab OR ferrum:ti,ab

#3 #1 OR #2

#4 'ready to use therapeutic food'/exp OR 'fortified food'/exp OR 'ready to use supplementary food'/exp OR 'lipid based nutrient supplement'/exp OR 'dietary supplement'/exp

#5 ((enriched OR fortified OR supplement* OR therapeutic) NEXT/1 (food* OR diet*)):ti,ab

#6 rutf:ti,ab OR rutfs:ti,ab OR 'ready to use therapeutic food*':ti,ab OR 'ready to use therapeutic feed*':ti,ab OR rusf:ti,ab OR rusfs:ti,ab OR 'ready to use supplemental food*':ti,ab OR 'ready to use supplementary food*':ti,ab OR 'lipid based nutrient supplement*':ti,ab OR lns:ti,ab

#7 #4 OR #5 OR #6

#8 'malnutrition'/exp OR 'wasting syndrome'/exp OR 'cachexia'/exp

For peer review only - http://bmjopen.bmj.com/site/about/guidelines.xhtml

#9 (nutrition* NEAR/1 deficien*):ti,ab

#10 'severe acute malnutrition':ti,ab OR sam:ti,ab OR malnutrition:ti,ab OR malnourish*:ti,ab OR cachexia:ti,ab OR undernutrition:ti,ab OR undernourish*:ti,ab OR wasting:ti,ab OR wasted:ti,ab

#11 #8 OR #9 OR #10

#12 'infant'/exp OR 'infancy'/exp OR 'baby'/exp OR 'newborn'/exp OR 'toddler'/exp OR 'child'/exp OR 'preschool child'/exp OR 'juvenile'/exp OR 'girl'/exp OR 'boy'/exp

#13 infant*:ti,ab OR infancy:ti,ab OR baby:ti,ab OR babies:ti,ab OR newborn*:ti,ab OR neonat*:ti,ab OR 'neo nat*':ti,ab OR child*:ti,ab OR toddler*:ti,ab OR youth:ti,ab OR juvenile*:ti,ab OR girl*:ti,ab OR boy:ti,ab OR boys:ti,ab OR preschool*:ti,ab OR 'pre school*':ti,ab

#14 #12 OR #13

#15 #3 AND #7 AND #11 AND #14

#16 #15 NOT [medline]/lim

SCOPUS

(TITLE-ABS (iron OR 56fe OR fe OR Ferro OR ferrum)) AND ((TITLE-ABS (enriched OR fortified OR supplement* OR therapeutic) PRE/1 TITLE-ABS (food* OR diet*)) OR (TITLE-ABS (rutf OR rutfs OR "ready to use therapeutic food*" OR "ready to use therapeutic feed*" OR rusf OR rusfs OR "ready to use supplemental food*" OR "ready to use supplementary food*" OR "lipid-based nutrient supplement*" OR lns))) AND ((TITLE-ABS (nutrition* W/1 deficien*)) OR (TITLE-ABS ("Severe Acute Malnutrition" OR sam OR malnutrition OR malnourish* OR cachexia OR undernutrition OR undernourish* OR wasting OR wasted))) AND (TITLE-ABS (infant* OR infancy OR baby OR babies OR newborn* OR neonat* OR neo-nat* OR child* OR toddler* OR youth OR juvenile* OR girl* OR boy OR boys OR preschool* OR pre-school*)) AND NOT INDEX (Medline)

CENTRAL

#1 MeSH descriptor: [Iron] in all MeSH products

#2 MeSH descriptor: [Iron, Dietary] explode all trees

#3 Iron:ti,ab OR 56Fe:ti,ab OR Fe:ti,ab OR ferro:ti,ab OR ferrum:ti,ab

#4 #1 OR #2 OR #3

#5 MeSH descriptor: [Food, Formulated] explode all trees

#6 MeSH descriptor: [Food, Fortified] explode all trees

#7 ((enriched OR fortified OR supplement* OR therapeutic) NEXT (food* OR diet*)):ti,ab

#8 (ready NEXT to NEXT use NEXT therapeutic NEXT food*):ti,ab

#9 (ready NEXT to NEXT use NEXT therapeutic NEXT feed*):ti,ab

#10 (ready NEXT to NEXT use NEXT supplemental NEXT food*):ti,ab

For peer review only - http://bmjopen.bmj.com/site/about/guidelines.xhtml

#11 (ready NEXT to NEXT use NEXT supplementary NEXT food*):ti,ab

#12 (lipid NEXT based NEXT nutrient NEXT supplement*):ti,ab

#13 rutf:ti,ab OR rutfs:ti,ab OR rusf:ti,ab OR rusfs:ti,ab OR lns:ti,ab

#14 #5 OR #6 OR #7 OR #8 OR #9 OR #10 OR #11 OR #12 OR #13

#15 MeSH descriptor: [Severe Acute Malnutrition] explode all trees

#16 MeSH descriptor: [Malnutrition] explode all trees

#17 MeSH descriptor: [Cachexia] explode all trees

#18 MeSH descriptor: [Wasting Syndrome] explode all trees

#19 (nutrition* NEAR/1 deficien*):ti,ab

#20 (severe NEXT acute NEXT malnutrition):ti,ab

#21 sam:ti,ab OR malnutrition:ti,ab OR malnourish*:ti,ab OR cachexia:ti,ab OR undernutrition:ti,ab OR undernourish*:ti,ab OR wasting:ti,ab OR wasted:ti,ab

#22 #15 OR #16 OR #17 OR #18 OR #19 OR #20 OR #21

#23 MeSH descriptor: [Infant] explode all trees

#24 MeSH descriptor: [Child] explode all trees

#25 infant*:ti,ab OR infancy:ti,ab OR baby:ti,ab OR babies:ti,ab OR newborn*:ti,ab OR neonat*:ti,ab OR neo NEXT nat*':ti,ab OR child*:ti,ab OR toddler*:ti,ab OR youth:ti,ab OR juvenile*:ti,ab OR girl*:ti,ab OR boy:ti,ab OR boys:ti,ab OR preschool*:ti,ab OR pre NEXT school*:ti,ab

#26 #23 OR #24 OR #25

#27 #4 AND #14 AND #22 AND #26

#28 "accession number" near pubmed

#29 #27 NOT #28

Web of Science

#1 TI=(iron OR 56fe OR fe OR ferro OR ferrum)

#2 AB=(iron OR 56fe OR fe OR ferro OR ferrum)

#3 #1 OR #2

#4 TI=(RUTF OR RUTFs OR "ready to use therapeutic food*" OR "ready to use therapeutic feed*" OR "therapeutic food*" OR "therapeutic diet*" OR "enriched food*" OR "enriched diet*" OR "fortified food*" OR "fortified diet*" OR "supplemented food*" OR "supplemental food*" OR "supplementary food*" OR "supplement food*" OR "supplemented diet*" OR "supplemental diet*" OR "supplementary diet*" OR "supplement diet*" OR RUSF OR RUSFs OR "ready to use supplemental food*" OR "ready to use supplementary food*" OR "lipid based nutrient supplement*" OR LNS)

#5 AB=(RUTF OR RUTFs OR "ready to use therapeutic food*" OR "ready to use therapeutic feed*" OR "therapeutic food*" OR "therapeutic diet*" OR "enriched food*" OR "enriched diet*" OR "fortified food*" OR "fortified diet*" OR "supplemented food*" OR "supplemental food*" OR "supplementary food*" OR

For peer review only - http://bmjopen.bmj.com/site/about/guidelines.xhtml

"supplement food*" OR "supplemented diet*" OR "supplemental diet*" OR "supplementary diet*" OR "supplement diet*" OR RUSF OR RUSFs OR "ready to use supplemental food*" OR "ready to use supplementary food*" OR "lipid based nutrient supplement*" OR LNS)

#6 #4 OR #5

#7 TI=(nutrition* NEAR/1 deficien*)

#8 AB=(nutrition* NEAR/1 deficien*)

#9 TI=("Severe Acute Malnutrition" OR sam OR malnutrition OR malnourish* OR cachexia OR undernutrition OR undernourish* OR wasting OR wasted)

#10 AB=("Severe Acute Malnutrition" OR sam OR malnutrition OR malnourish* OR cachexia OR undernutrition OR undernourish* OR wasting OR wasted)

#11 #7 OR #8 OR #9 OR #10

#12 TI=(infant* OR infancy OR baby OR babies OR newborn* OR neonat* OR "neo nat*" OR child* OR toddler* OR youth OR juvenile* OR girl* OR boy OR boys OR preschool* OR "pre school*")

#13 AB=(infant* OR infancy OR baby OR babies OR newborn* OR neonat* OR "neo nat*" OR child* OR toddler* OR youth OR juvenile* OR girl* OR boy OR boys OR preschool* OR "pre school*")

#14 #12 OR #13

#15 #3 AND #6 AND #11 AND #14

LILACS

(mh:("Iron")) OR ((mh:("iron, dietary"))) OR ((ti:(iron OR 56fe OR fe OR ferro OR ferrum))) OR ((ab:(iron OR 56fe OR fe OR ferro OR ferrum))) AND ((mh:("food, formulated")) OR ((mh:("food, fortified"))) OR ((ti:(rutf OR rutfs OR "ready to use therapeutic food*" OR "ready to use therapeutic feed*" OR "therapeutic food*" OR "therapeutic diet*" OR "enriched food*" OR "enriched diet*" OR "fortified food*" OR "fortified diet*" OR "supplemented food*" OR "supplemental food*" OR "supplementary food*" OR "supplement food*" OR "supplemented diet*" OR "supplemental diet*" OR "supplementary diet*" OR "supplement diet*" OR rusf OR rusfs OR "ready to use supplemental food*" OR "ready to use supplementary food*" OR "lipid based nutrient supplement*" OR lns))) OR ((ab:(rutf OR rutfs OR "ready to use therapeutic food*" OR "ready to use therapeutic feed*" OR "therapeutic food*" OR "therapeutic diet*" OR "enriched food*" OR "enriched diet*" OR "fortified food*" OR "fortified diet*" OR "supplemented food*" OR "supplemental food*" OR "supplementary food*" OR "supplement food*" OR "supplemented diet*" OR "supplemental diet*" OR "supplementary diet*" OR "supplement diet*" OR rusf OR rusfs OR "ready to use supplemental food*" OR "ready to use supplementary food*" OR "lipid based nutrient supplement*" OR lns)))) AND ((mh:("Severe Acute Malnutrition")) OR ((mh:("Malnutrition"))) OR ((mh:("cachexia"))) OR ((mh:("wasting syndrome"))) OR ((ti:("Severe Acute Malnutrition" OR sam OR malnutrition OR malnourish* OR cachexia OR "nutritional deficien*" OR undernutrition OR "deficient nutrition" OR undernourish* OR wasting OR wasted))) OR ((ab:("Severe Acute Malnutrition" OR sam OR malnutrition

For peer review only - http://bmjopen.bmj.com/site/about/guidelines.xhtml

OR malnourish* OR cachexia OR "nutritional deficien*" OR undernutrition OR "deficient nutrition" OR undernourish* OR wasting OR wasted)))) AND ((mh:(infant)) OR ((mh:(child))) OR ((ti:(infant* OR infancy OR baby OR babies OR newborn* OR neonat* OR "neo nat*" OR child* OR toddler* OR youth OR juvenile* OR girl* OR boy OR boys OR preschool* OR "pre school*"))) OR ((ab:(infant* OR infancy OR baby OR babies OR newborn* OR neonat* OR "neo nat*" OR child* OR toddler* OR youth OR juvenile* OR girl* OR boy OR boys OR preschool* OR "pre school*")))) AND (db:("LILACS"))

Global Index Medicus

((mh:("Iron")) OR ((mh:("iron, dietary"))) OR ((ti:(iron OR 56fe OR fe OR ferro OR ferrum))) OR ((ab:(iron OR 56fe OR fe OR ferro OR ferrum))) AND ((mh:("food, formulated")) OR ((mh:("food, fortified"))) OR ((ti:(rutf OR rutfs OR "ready to use therapeutic food*" OR "ready to use therapeutic feed*" OR "therapeutic food*" OR "therapeutic diet*" OR "enriched food*" OR "enriched diet*" OR "fortified food*" OR "fortified diet*" OR "supplemented food*" OR "supplemental food*" OR "supplementary food*" OR "supplement food*" OR "supplemented diet*" OR "supplemental diet*" OR "supplementary diet*" OR "supplement diet*" OR rusf OR rusfs OR "ready to use supplemental food*" OR "ready to use supplementary food*" OR "lipid based nutrient supplement*" OR lns))) OR ((ab:(rutf OR rutfs OR "ready to use therapeutic food*" OR "ready to use therapeutic feed*" OR "therapeutic food*" OR "therapeutic diet*" OR "enriched food*" OR "enriched diet*" OR "fortified food*" OR "fortified diet*" OR "supplemented food*" OR "supplemental food*" OR "supplementary food*" OR "supplement food*" OR "supplemented diet*" OR "supplemental diet*" OR "supplementary diet*" OR "supplement diet*" OR rusf OR rusfs OR "ready to use supplemental food*" OR "ready to use supplementary food*" OR "lipid based nutrient supplement*" OR lns)))) AND ((mh:("Severe Acute Malnutrition")) OR ((mh:("Malnutrition"))) OR ((mh:("cachexia"))) OR ((mh:("wasting syndrome"))) OR ((ti:("Severe Acute Malnutrition" OR sam OR malnutrition OR malnourish* OR cachexia OR "nutritional deficien*" OR undernutrition OR "deficient nutrition" OR undernourish* OR wasting OR wasted))) OR ((ab:("Severe Acute Malnutrition" OR sam OR malnutrition OR malnourish* OR cachexia OR "nutritional deficien*" OR undernutrition OR "deficient nutrition" OR undernourish* OR wasting OR wasted)))) AND ((mh:(infant)) OR ((mh:(child))) OR ((ti:(infant* OR infancy OR baby OR babies OR newborn* OR neonat* OR "neo nat*" OR child* OR toddler* OR youth OR juvenile* OR girl* OR boy OR boys OR preschool* OR "pre school*"))) OR ((ab:(infant* OR infancy OR baby OR babies OR newborn* OR neonat* OR "neo nat*" OR child* OR toddler* OR youth OR juvenile* OR girl* OR boy OR boys OR preschool* OR "pre school*"))))) AND (collection_gim:("IMSEAR" OR "IMEMR" OR "WPRIM"))

For peer review only - http://bmjopen.bmj.com/site/about/guidelines.xhtml

Author	Publication year	Name of Data Extractor	Form: conference abstract only (1), journal publication (2), multiple publications of the same trial (3)	Study Design	Study Setting		Population
					Country	Year(s) of Data Collection	Description of Participants
							Inclusion Criteria

For peer review only

	% Female (N) (Give for total study population)
Exclusion Criteria	

For peer review only

	Macronutrient composition of RUTF in the intervention group		Iron dos/100g of RUTF in the experimental group	Zinc dos/100 mg of RUTF in the experimental group	Frequency of RUTF (packets per day)
Total number of eligible participants in all the study groups					

For peer review only

Duration of intervention RUTF (How many days of intervention was given)	Characteristic of comparison		Study confounders		What outcome did authors report	Notes
	Standard RTUFT? (Yes or No)	Iron content in Standard RUTF (Normal dose is 10-14 mg/100 g)	Were results adjusted for confounders? (Y/N) (Fill only for observational studies)	What confounders were considered?		

For peer review only

Study (name, year)	Hemoglobin at longest follow up		
	No of events in interventional RUTF	Total number in interventional RUTF	number of events in control RUTF group

For peer review only

	**Summary estimate of intervention RUTF vs. Standard RUTF (OR, RR, HR)**		Intervention
number of participants in control RUTF group	notes	notes	Mean

For peer review only

RUTF		Control RUTF			Intervention in RUTF	
SD	Number in F	Mean	SD	Number in t	median	IQR

For peer review only

	Control RUTF		
Number in g	Median	IQR	Number in g

For peer review only

Summary estimate (mean difference or Standardized mean difference)

group

For peer review only

Notes

For peer review only

	A	B	C	D	E	F	G	H	I	J	K	L	M	N	O	P
1		**Anemia at longest follow up**				**Summary estimate of intervention RUTF vs. Standard RUTF (OR, RR, HR)**		Intervention RUTF			Control RUTF			Intervention in RUTF		
2	Study (name, year)	No of events in interventional RUTF	RUTF	control RUTF group	RUTF group	notes	notes	Mean	SD	RUTF	Mean	SD	control RUTF	median	IQR	group

For peer review only

	Q	R	S	T
1	Control RUTF			Summary estimate (mean difference or Standardized mean difference)
2	Median	IQR	group	

For peer review only

Study (name, year)	Iron deficiancy anemia at longest follow up		
	No of events in interventional RUTF	Total number in interventional RUTF	number of events in control RUTF group

For peer review only

	**Summary estimate of intervention RUTF vs. Standard RUTF (OR, RR, HR)**		Intervention RUTF			Control RUT
number of participants in control RUTF group	notes	notes	Mean	SD	Number in RUTF interventional group	Mean

For peer review only

TF		Intervention in RUTF			Control RUTF		
SD	Number in the control RUTF group	median	IQR	Number in group	Median	IQR	Number in group

For peer review only

Summary estimate (mean difference or Standardized mean difference)

For peer review only

Study (name, year)	SAM Recovery at longest follow up				Summary estimate of intervention RUTF vs. Standard RUTF (OR, RR, HR)	
	No of events in interventional RUTF	Total number in interventional RUTF	number of events in control RUTF group	number of participants in control RUTF group	notes	notes

For peer review only

Study (name, year)	Adverse effects at longest follow up				Summary estimate of intervention RUTF vs. Standard RUTF (OR, RR, HR)
	No of events in interventional RUTF	Total number in interventional RUTF	number of events in control RUTF group	number of participants in control RUTF group	notes

For peer review only

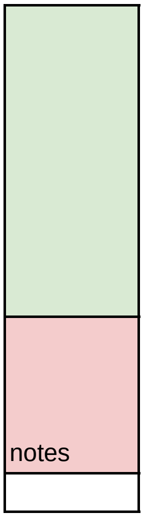

For peer review only

Study (name, year)	Adverse effects at longest follow up				Summary estimate of intervention RUTF vs. Standard RUTF (OR, RR, HR)	
	No of events in interventional RUTF	Total number in interventional RUTF	number of events in control RUTF group	number of participants in control RUTF group	notes	notes

For peer review only

	A	B	C	D	E	F	G	H	I	J	K	L	M	N
1	Study Name	Intervention RUTF			Control RUTF			Intervention in RUTF			Control RUTF			Summary esti
2		Mean	SD	Number in RUTF inter	Mean	SD	Number in the	median	IQR	Number in gro	Median	IQR	Number in group	
3														
4														

For peer review only

Study (name, year)	Adverse effects at longest follow up				Summary estimate of intervention RUTF vs. Standard RUTF (OR, RR, HR)	
	No of events in interventional RUTF	Total number in interventional RUTF	number of events in control RUTF group	number of participants in control RUTF group	notes	notes

For peer review only

	A	B	C	D	E	F	G	H	I	J	K	L	M	N	O	P	Q	R	S	T
1	Study ID (Last name and yea		Z scores						Kg						Change in weight (Kg) or Z scores					
2			Intervention			Control			Intervention			Control			Intervention			Control		
3			Mean	SD	Number in gro	Mean	SD	Number in gro	Mean	SD	Number in gro	Mean	SD	Number in gro	Mean	SD	Number in gro	Mean	SD	Number in gro
4																				
5																				

For peer review only

	U
1	Comments
2	
3	up
4	
5	

For peer review only

	A	B	C	D	E	F	G	H	I	J	K	L	M	N	O	P	Q	R	S	T
1	Study ID (Last name and yea		Z scores						Kg						Change in weight (Kg) or Z scores					
2			Intervention			Control			Intervention			Control			Intervention			Control		
3			Mean	SD	Number in gro	Mean	SD	Number in gro	Mean	SD	Number in gro	Mean	SD	Number in gro	Mean	SD	Number in gro	Mean	SD	Number in gro
4																				
5																				

For peer review only

	U
1	Comments
2	
3	up
4	
5	

For peer review only

	A	B	C	D	E	F	G	H	I	J	K	L	M	N	O	P	Q	R	S	T	U
1	Study ID (Last name and year)		Z scores						Centimeters						Change in height (cm) or Z scores						Comments
2			Intervention			Control			Intervention			Control			Intervention			Control			
3			Mean	SD	Number in gro	Mean	SD	Number in gro	Mean	SD	Number in gro	Mean	SD	Number in gro	Mean	SD	Number in gro	Mean	SD	Number in group	
4																					
5																					

For peer review only

	A	B	C	D	E	F	G	H	I	J	K	L	M	N	O	P	Q	R	S	T
1	Study ID (Last name and yea		Z scores						Centimeters						Change in height (cm) or Z scores					
2			Intervention			Control			Intervention			Control			Intervention			Control		
3			Mean	SD	Number in gro	Mean	SD	Number in gro	Mean	SD	Number in gro	Mean	SD	Number in gro	Mean	SD	Number in gro	Mean	SD	Number in gro
4																				
5																				

For peer review only

	U
1	Comments
2	
3	up
4	
5	

For peer review only

	A	B	C	D	E	F	G	H	I	J	K	L	M	N	O	P	Q	R	S	T
1	Study ID (Last name and year)		Z scores						Centimeters						Change in height (cm) or Z scores					
2			Intervention			Control			Intervention			Control			Intervention			Control		
3			Mean	SD	Number in gro	Mean	SD	Number in gro	Mean	SD	Number in gro	Mean	SD	Number in gro	Mean	SD	Number in gro	Mean	SD	Number in gro
4																				
5																				

For peer review only

	U
1	Comments
2	
3	up
4	
5	

For peer review only

	A	B	C	D	E	F	G	H	I	J	K	L	M	N	O	P	Q	R	S	T
1	Study ID (Last name and year)		Z scores						BMI						Change in BMI or Z scores					
2			Intervention			Control			Intervention			Control			Intervention			Control		
3			Mean	SD	Number in gro	Mean	SD	Number in gro	Mean	SD	Number in gro	Mean	SD	Number in gro	Mean	SD	Number in gro	Mean	SD	Number in gro
4																				
5																				

For peer review only

	U
1	Comments
2	
3	up
4	
5	

For peer review only

Study ID (Last name and year)	Z scores						BMI						Change in BMI or Z scores						Comments
	Intervention			Control			Intervention			Control			Intervention			Control			
	Mean	SD	Number in gro	Mean	SD	Number in gro	Mean	SD	Number in gro	Mean	SD	Number in gro	Mean	SD	Number in gro	Mean	SD	Number in group	

For peer review only

Study ID (Last name and year	Z scores						BMI						Change in BMI or Z scores						Comments
	Intervention			Control			Intervention			Control			Intervention			Control			
	Mean	SD	Number in gro	Mean	SD	Number in gro	Mean	SD	Number in gro	Mean	SD	Number in gro	Mean	SD	Number in gro	Mean	SD	Number in group	

For peer review only

Routine antibiotics for infants less than six months of age with growth failure/faltering. A protocol for the systematic review

Aamer Imdad, Fanny F Chen, Melissa Francois, Emily Tanner-Smith, Abigail Smith, Olivia J Tsistinas, Jai Das, Zulfiqar Ahmed Bhutta

PRISMA-P (Preferred Reporting Items for Systematic review and Meta-Analysis Protocols) 2015 checklist: recommended items to address in a systematic review protocol*

Section and topic	Item No	Checklist item	This manuscript
**ADMINISTRATIVE INFORMATION**			
Title:			
Identification	1a	Identify the report as a protocol of a systematic review	Page 1
Update	1b	If the protocol is for an update of a previous systematic review, identify as such	
Registration	2	If registered, provide the name of the registry (such as PROSPERO) and registration number	Application pending
Authors:			
Contact	3a	Provide name, institutional affiliation, e-mail address of all protocol authors; provide physical mailing address of corresponding author	Page 1
Contributions	3b	Describe contributions of protocol authors and identify the guarantor of the review	Page 11
Amendments	4	If the protocol represents an amendment of a previously completed or published protocol, identify as such and list changes; otherwise, state plan for documenting important protocol amendments	Not Applicable
Support:			
Sources	5a	Indicate sources of financial or other support for the review	Page 11
Sponsor	5b	Provide name for the review funder and/or sponsor	Page 11
Role of sponsor or funder	5c	Describe roles of funder(s), sponsor(s), and/or institution(s), if any, in developing the protocol	Page 11
**INTRODUCTION**			
Rationale	6	Describe the rationale for the review in the context of what is already known	Page 5
Objectives	7	Provide an explicit statement of the question(s) the review will address with reference to participants, interventions, comparators, and outcomes (PICO)	Page 6 and 7
**METHODS**			

For peer review only

Eligibility criteria	8	Specify the study characteristics (such as PICO, study design, setting, time frame) and report characteristics (such as years considered, language, publication status) to be used as criteria for eligibility for the review	Page 6
Information sources	9	Describe all intended information sources (such as electronic databases, contact with study authors, trial registers or other grey literature sources) with planned dates of coverage	Page 7
Search strategy	10	Present draft of search strategy to be used for at least one electronic database, including planned limits, such that it could be repeated	Appendix 1
Study records:			
Data management	11a	Describe the mechanism(s) that will be used to manage records and data throughout the review	Page 8
Selection process	11b	State the process that will be used for selecting studies (such as two independent reviewers) through each phase of the review (that is, screening, eligibility and inclusion in meta-analysis)	Page 8
Data collection process	11c	Describe planned method of extracting data from reports (such as piloting forms, done independently, in duplicate), any processes for obtaining and confirming data from investigators	Page 8
Data items	12	List and define all variables for which data will be sought (such as PICO items, funding sources), any pre-planned data assumptions and simplifications	Page 9
Outcomes and prioritization	13	List and define all outcomes for which data will be sought, including prioritization of main and additional outcomes, with rationale	Page 6 and 7
Risk of bias in individual studies	14	Describe anticipated methods for assessing risk of bias of individual studies, including whether this will be done at the outcome or study level, or both; state how this information will be used in data synthesis	Page 9
Data synthesis	15a	Describe criteria under which study data will be quantitatively synthesised	Page 9
	15b	If data are appropriate for quantitative synthesis, describe planned summary measures, methods of handling data and methods of combining data from studies, including any planned exploration of consistency (such as I^2, Kendall's τ)	Page 9
	15c	Describe any proposed additional analyses (such as sensitivity or subgroup analyses, meta-regression)	Page 10
	15d	If quantitative synthesis is not appropriate, describe the type of summary planned	Page 10
Meta-bias(es)	16	Specify any planned assessment of meta-bias(es) (such as publication bias across studies, selective reporting within studies)	Page 10
Confidence in cumulative evidence	17	Describe how the strength of the body of evidence will be assessed (such as GRADE)	Page 9 and 10

For peer review only

BMJ Open

Optimal iron content in ready-to-use therapeutic foods for the treatment of severe acute malnutrition in the community settings. A protocol for the systematic review and meta-analysis

Journal:	*BMJ Open*
Manuscript ID	bmjopen-2021-057389.R2
Article Type:	Protocol
Date Submitted by the Author:	16-Feb-2022

Complete List of Authors:	Imdad, Aamer; SUNY Upstate Medical University, Pediatrics<br>François, Melissa; SUNY Upstate Medical University, College of Medicine<br>Chen, Fanny F; SUNY Upstate Medical University<br>Smith, Abigail; SUNY Upstate Medical University, Library Sciences<br>Tsistinas, Olivia; SUNY Upstate Medical University, Library sciences<br>Tanner-Smith, Emily; University of Oregon, College of Education<br>Das, Jai K.; The Aga Khan University<br>Bhutta, Zulfiqar; The Aga Khan University

**Primary Subject Heading**:	Paediatrics
Secondary Subject Heading:	Global health, Nutrition and metabolism, Gastroenterology and hepatology
Keywords:	PAEDIATRICS, NUTRITION & DIETETICS, PUBLIC HEALTH

SCHOLARONE™ Manuscripts

Optimal iron content in ready-to-use therapeutic foods for the treatment of severe acute malnutrition in the community settings. A protocol for the systematic review and meta-analysis

Aamer Imdad, Melissa François, Fanny F Chen, Abigail Smith, Olivia J Tsistinas, Emily Tanner-Smith, Jai K Das, Zulfiqar Ahmed Bhutta

Authors' information

Corresponding Author
Aamer Imdad, MBBS, MPH
Assistant Professor of Pediatrics
Division of Pediatric Gastroenterology, Hepatology and Nutrition
SUNY Upstate Medical University, Syracuse, NY
725 Irving Street, Syracuse, NY 13210
Email: imdada@upstate.edu
Phone: 315-464-8444
Fax: 315-464-8443

Melissa François
SUNY Upstate Medical University
Syracuse, New York, USA
FrancoMe@upstate.edu

Fanny Chen
SUNY Upstate Medical University
Syracuse, New York, USA
ChenF@upstate.edu

Abigail Smith
SUNY Upstate Medical University
Syracuse, New York, USA
SmithAb@upstate.edu

Olivia Tsistinas

For peer review only

For peer review only - http://bmjopen.bmj.com/site/about/guidelines.xhtml

SUNY Upstate Medical University
Syracuse, New York, USA
TsistiJO@upstate.edu

Emily Tanner-Smith
University of Oregon
etanners@uoregon.edu
Eugene, Oregon, USA

Jai Das
Aga Khan University
Karachi, Sindh, Pakistan
Jai.das@aku.edu

For peer review only

For peer review only - http://bmjopen.bmj.com/site/about/guidelines.xhtml

Zulfiqar Ahmed Bhutta

Institute for Global Health & Development, The Aga Khan University

Karachi, Sindh, Pakistan

zulfiqar.bhutta@aku.edu

Word Count: 3265

For peer review only

For peer review only - http://bmjopen.bmj.com/site/about/guidelines.xhtml

ABSTRACT

Introduction

The current standard of care for children with severe acute malnutrition involves using ready-to-use therapeutic food (RUTF) to promote growth; however, the precise formulation to achieve optimal recovery remains unclear. Emerging research suggests that alternative RUTF formulations may be more effective in correcting severe acute malnutrition (SAM) related complications such as anemia and iron deficiency. This systematic review commissioned by the World Health Organization (WHO) aims to synthesize the most recent research on the iron content in RUTF and related products in the community-based treatment of uncomplicated severe malnutrition in children aged six months and older.

Methods and Analysis

We will search multiple electronic databases. We will include randomized controls trials and non-randomized studies with a control arm. The intervention group will be infants who received RUTF treatments other than the current recommended guidelines set forth by the WHO. The comparison group is children receiving RUTF containing iron at the current WHO recommended level of 1.9 mg/100 kcal (10-14 mg/100 g). The primary outcomes of interest include blood hemoglobin concentration, any anemia, severe anemia, iron deficiency anemia, recovery from severe acute malnutrition, and any adverse outcomes. We will use meta-analysis to pool findings if sufficient homogeneity exists among included studies. The risk of bias in studies will be evaluated using the Cochrane risk of bias-2 (ROB 2.0) and Cochrane ROBINS-I tools. We will use the Grading of Recommendations Assessment, Development, and Evaluation (GRADE) approach to examine the overall certainty of evidence.

Ethics and Dissemination: This is a systematic review and will not involve direct contact with human subjects. The findings of this review will be published in a peer-review journal and will guide the WHO's recommendation on the optimal iron content in ready-to-use therapeutic foods for the treatment of severe acute malnutrition in children aged 6-59 months.

Key Words: Iron, ready-to-use therapeutic food, wasting, malnutrition, growth failure

For peer review only

ARTICLE SUMMARY: STRENGTHS AND LIMITATIONS OF THIS STUDY

- This systematic review commissioned by the WHO will synthesize the most recent research on iron content in ready to use therapeutic foods and related products in the community-based treatment of uncomplicated severe malnutrition in children aged six months and older.
- We will search several databases for relevant literature and include randomized and non-randomized studies.
- We will assess risk-of-bias for each outcome and use the Grading of Recommendations Assessment, Development, and Evaluation to assess the overall quality of evidence.
- We will assess the following outcomes: blood hemoglobin concentration, any anemia, severe anemia, iron deficiency anemia, recovery rates, all-cause mortality, adverse events, growth outcome, and serum levels of micronutrients.
- We will conduct meta-analyses if data are available from more than one study and there is clinical and methodological homogeneity in the included studies.

For peer review only

For peer review only - http://bmjopen.bmj.com/site/about/guidelines.xhtml

For peer review only

Full manuscript

BACKGROUND

The World Health Organization (WHO) estimates over 45 million children under the age of five worldwide suffered from wasting (low weight for height) in 2020 alone[1]. Children with severe acute malnutrition (SAM) have about 6 fold increased mortality risk compared to well-nourished children[2]. In addition to the risk of mortality, children with SAM are at increased risk of morbidities such as diarrhea, pneumonia, measles, and micronutrient deficiencies, and long-term neurodevelopmental delay[2-4]. Nutritional rehabilitation of children with SAM is a key intervention that helps treat morbidity and prevent mortality[3,5,6]. The current standard of care for children with uncomplicated severe acute malnutrition involves using ready-to-use therapeutic food (RUTF) to promote growth[7]; however, the precise formulation to achieve optimal recovery remains unclear[6,8]. There are ongoing efforts to improve RUTF to optimize the composition and balance of macronutrients and micronutrients[9-11].

Severe acute malnutrition involves deficits of several key macronutrients and micronutrients, with studies showing higher rates of anemia in children suffering from malnutrition [12-14]. The

For peer review only - http://bmjopen.bmj.com/site/about/guidelines.xhtml

prevalence of anemia in severely acutely malnourished children ranges from 40% to 90%, and about half of the anemia is attributed to iron deficiency[15]. Iron deficiency in severely malnourished children could be due to low intake, increased losses, higher demand, and poor absorption. Iron is essential for adequate catch-up growth and neurological development in children with severe acute malnutrition[12,16,17]. Moreover, recent studies suggest alternative RUTF formulations may be more effective in correcting anemia and iron deficiency in children with severe acute malnutrition compared to current standard RUTF formulations [12,18,19]. In addition to the need for sufficient iron intake to treat iron deficiency, it remains unclear whether improving the iron status among the undernourished children increases the risk of complications, including the risk of morbidities such as diarrhea, malaria, and undesired changes in the microbiome[20-22]. This aspect is especially of concern in malaria-endemic regions in Africa because nearly a third (27%) of all children affected by wasting worldwide reside in Africa[22]. Due to the critical role of RUTF in the medical management of children with severe acute malnutrition, more information is needed to generate formulations with optimal iron levels for treatment of anemia and optimal growth and development in children suffering from malnutrition [16-18]. A number of recent studies have been completed to assess the optimal dose of iron in RUTF in the community management of SAM, and there is no existing systematic review on this topic[12,19,23]. Furthermore, WHO has started to synthesize the evidence to update the current guidelines for treating wasting in children. Therefore, this WHO commissioned review aims to synthesize the most recent research on the iron content in RUTF and related products in treating severe malnutrition in children 6-59 months of age.

OBJECTIVE

Primary Objective*:* In children aged 6 months or older with uncomplicated severe acute malnutrition being treated in the community settings, does increased iron dose in RUTF compared with the WHO recommendation for iron fortification of RUTF improve outcomes such as blood hemoglobin concentration, recovery from iron-deficiency anemia, etc.?

Secondary Objective*:* In children aged 6 months or older with uncomplicated severe acute malnutrition being treated in the community settings, what mechanisms other than increased dose of iron in RUTF (such as type, composition, zinc, phytate content etc.) can help increase the bioavailability of iron and improve the recovery from iron deficiency anemia?

For peer review only - http://bmjopen.bmj.com/site/about/guidelines.xhtml

METHODS AND ANALYSIS

We will follow the standard guidelines of the Cochrane Collaboration to conduct the systematic review. We registered a detailed protocol on PROSPERO (ID=CRD42021278006) and would report the systematic review findings according to PRISMA 2020 guidelines[24].

Study type: We will consider randomized trials, and that may include individual and cluster randomized trials. We will also consider non-randomized studies if there are not enough randomized studies. The data will be analyzed separately for randomized and non-randomized studies. We will include the following non-randomized studies: Non-randomized controlled clinical trials and controlled before and after studies (CBAs). Case-control studies, interrupted time series, program evaluations, case reports, case series, and commentaries will be excluded. The definitions of eligible studies design and consideration for inclusion in this review are available in table 1.

Population

The population of interest is children aged 6 months or older with severe acute malnutrition managed in outpatient settings. The definition of severe acute malnutrition will be based on weight for height z scores (< -3 SD for WHO growth standards) or mid-upper arm circumference (< 115 mm) or presence of bilateral edema[25]. We will consider studies that included children who were previously admitted to the hospital and are now being rehabilitated in community settings with the help of RUTF. We will exclude studies of children with complicated SAM who are admitted to the hospital, as those participants might have complications such as pneumonia or severe diarrhea. We will exclude studies done specifically on participants who have chronic diseases, genetic disorders, or congenital anomalies.

Intervention

The intervention of interest is the dose of iron in RUTF other than in the standard RUTF for the treatment of severe acute malnutrition in community settings. We will include studies of iron-fortified RUTF and exclude those studies where iron was given separately as supplements, such as tablets, syrup, multiple micronutrient powder, etc. We will include studies where RUTF

For peer review only - http://bmjopen.bmj.com/site/about/guidelines.xhtml

is given as the main intervention, and it met total daily requirements or was given as a supplementary intervention to the usual diet for children with severe acute malnutrition recovering at home. WHO recommends that a standard RUTF should have at least 1.9 mg/100 kcal (or 10-14 mg/100 g) of iron to treat severe acute malnutrition in children 6-59 months of age[25]. We will include studies irrespective of the type of RUTF used, i.e., standard RUTF vs. low milk-based vs. non-milk-based vs. locally prepared. We will exclude studies where RUTF was given to healthy children to prevent severe acute malnutrition; treatment of moderate acute malnutrition; as part of complementary feeding.

Comparison

For the primary objective of the review, the comparison group will be children receiving RUTF containing iron at the current WHO recommended level of 1.9 mg/100 kcal (10-14 mg/100 g) to treat severe acute malnutrition. For the secondary objective of the review, we will consider studies where different types/doses/techniques of RUTF are studied to improve the bioavailability of iron even though the iron content may be the same in all kinds of RUTF given to children. We will exclude studies where the comparison group did not receive any RUTF.

OUTCOMES

Primary Outcomes

- Blood hemoglobin concentration (continuous outcome)
- Any Anemia (dichotomous outcome, as defined by authors)
- Severe anemia (dichotomous outcome, as defined by authors)
- Iron deficiency anemia (dichotomous outcome as defined by authors)
- Recovery from severe acute malnutrition (dichotomous outcome, as defined by authors)
- Any Adverse events (Dichotomous outcome)

Secondary Outcomes

- Serum ferritin level (continuous outcome)
- Serum zinc level (continuous outcome)
- Serum copper level (continuous outcome)
- Serum iron level (continuous outcome)
- Adverse events Malaria: (as defined by authors) (dichotomous outcome)
- Relapse (dichotomous outcome)

For peer review only - http://bmjopen.bmj.com/site/about/guidelines.xhtml

- All-cause mortality (dichotomous outcome)
- Admission to an inpatient facility. (dichotomous outcome)
- Withdrawal from the trial (Dichotomous outcome)
- Constipation (<3 bowel movements per week) (dichotomous outcome)
- Adverse events Diarrhea: (>3 loose stools per day) (dichotomous outcome)
- Adverse events: Pneumonia (as defined by authors) (dichotomous outcome)
- Weight for age (kg or Z scores)
- Height for age (cm or Z scores)
- Weight for height Z score
- Microbiome outcomes: Alpha diversity and Beta Diversity
- Neurodevelopmental outcomes (continuous outcome) at 1 year and the longest follow-up.

  The term neurodevelopment is a composite term that refers to cognitive, neurological, and/or sensory outcomes. This assessment may include intellectual disability as measured on the Mental Developmental Index of the Bayley Scales of Infant Development, gross motor delay measured on the Gross Motor Function Classification System, and so on.

We will consider the data for all the above outcomes at the longest follow-up.

LITERATURE SEARCH

We will conduct systematic electronic queries using key terms in several databases, including PubMed, EMBASE, the Cochrane Central Register for Controlled Trials, Web of Science, CINHAL,

Scopus, LILACS, and WHO Global Index Medicus. No search restrictions will be used to exclude studies based on the outcome, publication year, publication status, or language. The references of formerly published reviews and recently published studies will be examined for potential inclusion. We will also use the citation tracking function of the included studies in the PubMed to look for any eligible studies. In addition to the above resources, ClinicalTrials.gov will be used to identify studies currently underway. We will also use the clinical trial registration number to find all the relevant studies published from a particular trial. Finally, we will search the grey literature and search the websites of pertinent international agencies such as WHO

For peer review only

For peer review only - http://bmjopen.bmj.com/site/about/guidelines.xhtml

(including WHO's Reproductive Health Library, electronic Library of Evidence for Nutrition Actions and Global database on the Implementation of Nutrition Action), UNICEF, Global Alliance for Improved Nutrition, International Food Policy Research Institute, International Initiative for Impact Evaluation (3ie), Nutrition International, World Bank, USAID and affiliates (e.g., FANTA, SPRING), and the World Food Program. We will also search the abstracts presented in major pediatric conferences such as Pediatric academic society meeting. Proposed search strategies for different electronic databases are shown in Appendix 1.

DATA EXTRACTION AND SYNTHESIS

Selection of studies

Searches from all the databases will be combined in bibliographic software (EndNote), and duplicates will be removed. Two authors will use a three-phase approach in duplicate to screen studies identified from the search for eligibility. In the first phase, authors will screen the titles and abstracts to identify potentially eligible ones; studies selected during this initial phase will then go on to a full-text review as the second phase. Lastly, studies determined to be eligible subsequent to full-text review will undergo data extraction during the third and final phase. The software Covidence14[26] will be used to assist the screening process. Two authors will independently extract data from screened studies and compare their findings. Potential conflicts will be resolved through discussion, and the senior author on the team may assist as needed to resolve any conflicts. If a study is only available in abstract form, we will write to authors to obtain details on methods and results. If we cannot get full details of the study methods available in the abstract, we will decide about inclusion based on details available in the abstract. If a study is available in a language other than English, we will attempt to complete the translation using local resources. If a study was published in more than one report (multiple publications), we will count those multiple reports as a single study and extract information from all the available reports as needed.

Data extraction

A data extraction form was designed to support the review and data extraction process (supplementary document 1). Two authors will independently extract the data and compare their findings. Any conflict will be resolved by discussion and with the help of the senior author on the team if needed. The following information will be extracted for each study where available: study

For peer review only - http://bmjopen.bmj.com/site/about/guidelines.xhtml

design (randomized control trial, quasi-randomized experimental design or cohort study, CBA), study site (country/region), study year, study type, intervention (dose of iron and dose of RUTF, duration, frequency, and composition), exposures, comparison, outcomes, whether the results were adjusted for confounders, and risk of bias. To avoid reviewer bias, we are deciding a priori the order of preference for extracting outcomes when data might be available in several formats. For randomized studies, we will prefer data that require the least manipulation by authors or inference by reviewers. We will extract the raw values (for example, means and standard deviations) rather than calculated effect sizes (e.g., Cohen's d). For mortality data, we will give preference to denominators in the following order: number with the definite outcome known (or imputed as described below), number randomized, and child-years. For morbidity outcomes to which both survivors and non-survivors may have contributed data (for example, the incidence of pneumonia), we will give preference to child years, number with the definite outcome known, and number randomized.

Studies with missing data

We will document attrition during data extraction. If data are missing for some cases, or if reasons for dropout are not reported, we will contact the trial authors to request the full data. If the authors report the missing data and report results using imputations for the missing data, we will use the latter. If a study does not report the standard deviation (SD) for the continuous outcome and the SD cannot be calculated from the reported data (such as standard error, confidence interval, p-value), we will write to the authors to request the data for SD. If the SD data are not available from authors, we will use SD from a similar study that has a similar study population. We will prefer to use the final values of a continuous outcome for a given follow-up. If the final values are not available but the difference between the end and the start of the study, we will write to authors to request the final values. If the final values are not available, we will use the difference or rate of change. We will use the data based on intention to treat analysis. If the data for intention to treat analysis is not given in the study, we will create our own intention to treat analysis for participants with known outcomes. If there is significant attrition between the randomized participants vs. participants completing the study, we will include such a study but will investigate further with sensitivity analysis.

Assessment of risk of bias in included studies

For peer review only - http://bmjopen.bmj.com/site/about/guidelines.xhtml

The risk of bias in studies will be evaluated using the Cochrane risk of bias (ROB 2.0) and Cochrane ROBINS-I for non-randomized and observational studies [27,28]. Two review authors will independently assess and agree on the risk of bias for the individual studies for an outcome. The discussion will resolve any disagreements, and if no agreement can be made, the senior review author will be consulted. We will assess the effect of assignment to intervention (the intention-to-treat effect) for randomized trials by addressing five domains of signaling questions in ROB-2 including: bias arising from the randomization process, bias due to deviations from intended interventions, bias due to missing outcome data, bias in the measurement of the outcome and bias in the selection of the reported result. Each domain will receive a ranking of one of the following: low risk of bias, some concerns of bias, or high risk of bias. We will include quotes from the study for each signaling question as evidence for our ranking decision. The overall risk of bias will be determined based on the lowest ranking for individual domains. For example, if only one domain is ranked 'some concerns,' then the overall risk of bias will be 'some concerns'.

Data Synthesis

The findings from the systematic review will be reported both qualitatively and quantitatively. A narrative synthesis will be used to report the characteristics of all included studies. We will also narratively describe the data in a table for the secondary objective of this review (table 2). A random-effects meta-analysis will be employed when at least two studies possess sufficient clinical and methodological uniformity for synthesis for the primary objective of the review. We will use the generic inverse method to pool the studies in the meta-analysis. The software RevMan will be utilized for statistical analysis[29]. Dichotomous outcomes will be assessed using relative risk effect sizes and presented with 95% confidence intervals. In the case of morbidity outcomes, we will combine all available data whenever possible if outcomes are measured in different ways. For example, we will include all types of diarrhea (mild, moderate, and severe) as a dichotomous value (yes/no) if participants had greater than 3 instances of loose stools per day. We will include the occurrence of anemia, iron deficiency anemia, and pneumonia throughout the study as dichotomous values (yes/no). We will pool the data for continuous outcomes to obtain an average mean difference and report it with its 95% CI. If data are reported in different units (e.g., few studies report weight in kg and the others report in Z scores), we will use a standardized mean difference effect size and report it with its 95% CI. We will pool the data separately from randomized and observational studies. If an observational

For peer review only - http://bmjopen.bmj.com/site/about/guidelines.xhtml

study reports both adjusted and unadjusted values, we will use the adjusted values for meta-analysis.

We will consider the following pairs of comparisons:
High iron content in RUTF vs. WHO standard iron content in RUTF

The Grading of Recommendations Assessment, Development, and Evaluation (GRADE) approach will be used to evaluate overall evidence quality using the software GRADEpro[30]. The GRADE approach is a comprehensive framework used to assess the overall quality of evidence for an outcome using characteristics such as study design, heterogeneity, directness of evidence, risk of bias, publication bias, and precision of effect estimates[31]. The results of the GRADE assessment will be included in a summary of the findings table. The table will contain quality ratings characterized as very low (we have very little confidence in the effect estimate), low (we have limited confidence in the effect estimate), moderate (we have moderate confidence in the effect estimate; the true effect is likely close to the estimate of the effect), or high based (we have high confidence that the true effect lies close to that of the estimate of the effect) on the primary outcomes of each study (Table 3). The study will be started on February 18, 2022, and hope to be completed by April 18, 2022.

Patient and public involvement

No patient or public involvement was considered in the preparation of this protocol.

UNIT OF ANALYSIS ISSUES

Multiple-arm trial

Studies with multiple treatment arms will be included if eligible. For multiple-arm trials, we will include data so that the only difference between the groups is the use of iron in RUTF.

Cluster trials

Cluster assignment trials will be analyzed together with individually randomized trials. We will use the cluster adjusted values; if the trial results are not adjusted for cluster design, we will adjust the result by methods given in the Cochrane[32] handbook for systematic reviews.

For peer review only - http://bmjopen.bmj.com/site/about/guidelines.xhtml

Assessment of heterogeneity

Statistical heterogeneity of effect sizes within any given meta-analysis will be assessed using the χ^2, I^2, and tau statistics. We will assess statistical heterogeneity by visual inspection of forest plots, performing

the χ^2test (assessing the p-value), and calculating the Tau^2 and I^2 statistics. Statistical heterogeneity will be considered significant if the p-value is less than 0.10, I^2 value exceeds 50%, and the examination of forest plots shows substantial variability in the effect of the intervention. We will perform subgroup analysis to determine the reasons for any identified statistical heterogeneity.

Assessment of reporting bias

Small study and publication bias will be assessed using funnel plots and regression tests for funnel plot asymmetry when a meta-analysis includes at least ten studies.

Subgroup analyses

- Settings: Country: Low-income country vs. middle-income country vs. high-income country
- Type of RUTF: Standard RUTF vs. Non-standard
- Role of RUTF: RUTF as main treatment vs. RUTF as a supplementary intervention
- Type of participants: Studies that included children with HIV vs. Studies with children without HIV.
- Age: <24 months vs. 24-59 months vs. > 59 months
- Hospitalization: Children hospitalized (due to medical complication) prior to starting RUTF vs. Children not hospitalized prior to starting RUTF
- Iron compound (formulation/chemical compound and amount)
- Dose: The intervention groups with a dose of iron > standard WHO RUTF Vs. Intervention group receiving dose lower the Standard WHO RUTF
- Anemia Status: Children with anemia at the baseline vs. without anemia at the baseline
- Time of follow up: 1 month vs. 3 months vs. 6 months follow up and the longest follow up

Sensitivity analyses:

-Studies with a high risk of bias

-Random vs. fixed effect model

For peer review only

For peer review only - http://bmjopen.bmj.com/site/about/guidelines.xhtml

Amendments

We will do the literature searches, screening of titles, selection of studies, data extraction, and analysis according to the aforementioned plan described in this protocol. If we do any additional analysis or change any of the a priori strategies, we will clearly describe that in the methods section.

Consent for publication

The authors give consent for the publication of the review.

Competing interests

None declared.

Availability of data and materials

We will provide data available for review by the editors and peer reviewers and provide raw data for the general public on request.

Funding

This work is funded by World Health Organization grant no 202712614.

Role of Funding source

WHO provided technical support for this work and guided the development of PICO (Population, Intervention, Comparison, Outcome) question for this review.

Acknowledgments

We are very thankful to Allison Daniel, Jaden Bendabenda, Lisa Rogers, and Maria De Las Nieves Garcia Casal for their input to improve this protocol.

Authors' contributions

AI and MF wrote the first draft of the manuscript. AS and OT developed the search strategy. ET-S

For peer review only - http://bmjopen.bmj.com/site/about/guidelines.xhtml

provided the support for methodology. ZAB, FC and JD provided the support for the content being evaluated in the review. All authors edited and reviewed the manuscript. AI is the guarantor of the review.

ETHICS AND DISSEMINATION

This is a systematic review and will not involve direct contact with human subjects. The findings of this review will be published in a peer-review journal and will guide the WHO's recommendation on the optimal iron content in ready-to-use therapeutic foods for the treatment of severe acute malnutrition in children aged 6-59 months.

For peer review only

For peer review only - http://bmjopen.bmj.com/site/about/guidelines.xhtml

REFERENCES

1. United Nations Children's Fund (UNICEF), World Health Organization, International Bank for Reconstruction and Development/The World Bank. *Levels and trends in child malnutrition: key findings of the 2021 edition of the joint child malnutrition estimates*. Geneva: World Health Organization; 2021.
2. Schroeder DG, Brown KH. Nutritional status as a predictor of child survival: summarizing the association and quantifying its global impact. *Bull World Health Organ.* 1994;72(4):569-579.
3. Dipasquale V, Cucinotta U, Romano C. Acute Malnutrition in Children: Pathophysiology, Clinical Effects and Treatment. *Nutrients.* 2020;12(8).
4. Mwene-Batu P, Bisimwa G, Baguma M, et al. Long-term effects of severe acute malnutrition during childhood on adult cognitive, academic and behavioural development in African fragile countries: The Lwiro cohort study in Democratic Republic of the Congo. *PLoS One.* 2020;15(12):e0244486.
5. Woeltje MM, Evanoff AB, Helmink BA, et al. Community-based management of acute malnutrition for infants under 6 months of age is safe and effective: analysis of operational data. *Public Health Nutr.* 2021:1-10.
6. Das JK, Salam RA, Saeed M, Kazmi FA, Bhutta ZA. Effectiveness of Interventions for Managing Acute Malnutrition in Children under Five Years of Age in Low-Income and Middle-Income Countries: A Systematic Review and Meta-Analysis. *Nutrients.* 2020;12(1).
7. WHO W, UNSCN, UNICEF. Community-based management of severe acute malnutrition. A joint statement by the World Health Organization, World Food Programme, United Nations Standing Committee on Nutrition, United Nations Children's Fund. Geneva, World Health Organization. 2007.
8. Weisstaub G, Medina M, Pizarro F, Araya M. Copper, iron, and zinc status in children with moderate and severe acute malnutrition recovered following WHO protocols. *Biol Trace Elem Res.* 2008;124(1):1-11.
9. Potani I, Spiegel-Feld C, Brixi G, et al. Ready-to-Use Therapeutic Food (RUTF) Containing Low or No Dairy Compared to Standard RUTF for Children with Severe Acute Malnutrition: A Systematic Review and Meta-Analysis. *Adv Nutr.* 2021;12(5):1930-1943.
10. Schoonees A, Lombard MJ, Musekiwa A, Nel E, Volmink J. Ready-to-use therapeutic food (RUTF) for home-based nutritional rehabilitation of severe acute malnutrition in children from six months to five years of age. *Cochrane Database Syst Rev.* 2019;5:CD009000.
11. Shokry E, Sadiq K, Soofi S, et al. Impact of Treatment with RUTF on Plasma Lipid Profiles of Severely Malnourished Pakistani Children. *Nutrients.* 2020;12(7).
12. Akomo P, Bahwere P, Murakami H, et al. Soya, maize and sorghum ready-to-use therapeutic foods are more effective in correcting anaemia and iron deficiency than the standard ready-to-use therapeutic food: randomized controlled trial. *BMC Public Health.* 2019;19(1):806.
13. Savadogo LGB, Zoetaba I, Ilboudo B, Kinda M, Donnen P. Impact of anemia on mortality and nutritional recovery among hospitalized severely malnourished children in Burkina Faso. *Open Journal of Pediatrics.* 2014;2014.

For peer review only - http://bmjopen.bmj.com/site/about/guidelines.xhtml

14. Thakur N, Chandra J, Pemde H, Singh V. Anemia in severe acute malnutrition. *Nutrition.* 2014;30(4):440-442.
15. Kangas ST, Salpeteur C, Nikiema V, et al. Vitamin A and iron status of children before and after treatment of uncomplicated severe acute malnutrition. *Clin Nutr.* 2020;39(11):3512-3519.
16. Babirekere-Iriso E, Mortensen CG, Mupere E, et al. Changes in whole-blood PUFA and their predictors during recovery from severe acute malnutrition. *Br J Nutr.* 2016;115(10):1730-1739.
17. Jones KD, Ali R, Khasira MA, et al. Ready-to-use therapeutic food with elevated n-3 polyunsaturated fatty acid content, with or without fish oil, to treat severe acute malnutrition: a randomized controlled trial. *BMC Med.* 2015;13:93.
18. Lopriore C, Guidoum Y, Briend A, Branca F. Spread fortified with vitamins and minerals induces catch-up growth and eradicates severe anemia in stunted refugee children aged 3-6 y. *Am J Clin Nutr.* 2004;80(4):973-981.
19. Bahwere P, Balaluka B, Wells JC, et al. Cereals and pulse-based ready-to-use therapeutic food as an alternative to the standard milk- and peanut paste-based formulation for treating severe acute malnutrition: a noninferiority, individually randomized controlled efficacy clinical trial. *Am J Clin Nutr.* 2016;103(4):1145-1161.
20. Soofi S, Cousens S, Iqbal SP, et al. Effect of provision of daily zinc and iron with several micronutrients on growth and morbidity among young children in Pakistan: a cluster-randomised trial. *Lancet.* 2013;382(9886):29-40.
21. Paganini D, Zimmermann MB. The effects of iron fortification and supplementation on the gut microbiome and diarrhea in infants and children: a review. *Am J Clin Nutr.* 2017;106(Suppl 6):1688S-1693S.
22. Sazawal S, Black RE, Ramsan M, et al. Effects of routine prophylactic supplementation with iron and folic acid on admission to hospital and mortality in preschool children in a high malaria transmission setting: community-based, randomised, placebo-controlled trial. *Lancet.* 2006;367(9505):133-143.
23. Irena AH, Bahwere P, Owino VO, et al. Comparison of the effectiveness of a milk-free soy-maize-sorghum-based ready-to-use therapeutic food to standard ready-to-use therapeutic food with 25% milk in nutrition management of severely acutely malnourished Zambian children: an equivalence non-blinded cluster randomised controlled trial. *Matern Child Nutr.* 2015;11 Suppl 4:105-119.
24. Page MJ, McKenzie JE, Bossuyt PM, et al. The PRISMA 2020 statement: an updated guideline for reporting systematic reviews. *BMJ.* 2021;372:n71.
25. WHO. Management of severe malnutrition : a manual for physicians and other senior health workers. 1999.
26. Covidence systematic review software vhi, Melbourne, Australia. Available: www.covidence.org.
27. Sterne JA, Hernan MA, Reeves BC, et al. ROBINS-I: a tool for assessing risk of bias in non-randomised studies of interventions. *BMJ.* 2016;355:i4919.
28. Sterne JAC, Savovic J, Page MJ, et al. RoB 2: a revised tool for assessing risk of bias in randomised trials. *BMJ.* 2019;366:l4898.

For peer review only - http://bmjopen.bmj.com/site/about/guidelines.xhtml

29. Review Manager (RevMan) [Computer program]. Version 5.3. Copenhagen: The Nordic Cochrane Centre TCC. Version 5.3. Copenhagen: The Nordic Cochrane Centre, The Cochrane Collaboration. Published 2014. Accessed. .
30. GRADEpro GDT: GRADEpro Guideline Development Tool [Software]. McMaster University, 2020 (developed by Evidence Prime, Inc.). Available from gradepro.org.
31. Guyatt G, Oxman AD, Akl EA, et al. GRADE guidelines: 1. Introduction-GRADE evidence profiles and summary of findings tables. *J Clin Epidemiol.* 2011;64(4):383-394.
32. Higgins JPT TJ CJ, Cumpston M. Cochrane handbook for systematic reviews of interventions version 6.2, 2021. https://training.cochrane.org/handbook/PDF/v6.2.
33. (EPOC) CEPaOoC. What study designs can be considered for inclusion in an EPOC review and what should they be called? Avaialble at https://epoc.cochrane.org/sites/epoc.cochrane.org/files/public/uploads/EPOC%20Study%20Designs%20About.pdf. July 2021.

For peer review only

Table 1: The definitions of Eligible Study Designs

Study Designs	Definition	Notes
Randomized controlled trial (RCT) OR randomized trial	An experimental study in which people are allocated to different interventions using methods that are random."	We will include both individual and cluster randomized trials. In individual randomized trials, the randomized is done at the individual levels, while in cluster randomized trials, the randomized is based on cluster or groups of individuals. We will also consider factorial design trials where multiple interventions are studied in the same trial.
Non-randomised controlled trial (NRCT) OR non-randomised trial	"An experimental study in which people are allocated to different interventions using methods that are not random."	We will avoid using the term quasi experimental studies as it means differently by different authors. We will exclude the experimental studies where there was no control group.
Controlled before-after study (CBA)	"A study in which observations are made before and after the implementation of an intervention, both in a group that receives the intervention and in a control group that does not."	We will require two minimum criteria for the inclusion of CBAs.<br>-Data Collection: We will include CBAs if the data for the intervention and control groups were collected prospectively in the same time frame<br>-Choice of Control: We will include CBAs that include a control at a second site to avoid contamination of the intervention to the control group if the settings and populations are the same for the intervention and control groups.

Footnotes: The definitions of study designs were adopted from The Cochrane Effective Practice and Organization of Care (EPOC) group[33]

For peer review only

Table 2: Sample table to describe the studies that address delivery mechanism to increase the bioavailability of iron via RUTF in children 6 months and older with severe acute malnutrition

Study	Study Participants	Formulations used	Comparison group	Outcomes	Notes

For peer review only

Table 3: The Criteria for the Grading of Recommendations Assessment, Development, and Evaluation (GRADE) approach to examine the overall certainty of evidence[31]

<table>
<tr><th>Study Design</th><th>Quality of Evidence</th><th>Lower Certainty Score If</th><th>Higher Certainty Score If</th></tr>
<tr><td rowspan="2">Randomized trial</td><td>High</td><td rowspan="3">Risk of Bias
-1 Serious
-2 Very serious

Inconsistency
-1 Serious
-2 Very serious

Indirectness
-1 Serious
-2 Very serious

Imprecision</td><td rowspan="3">Large effect
+1 Large
+2 Very large

Dose-response
+1 evidence of a gradient

All plausible confounding would:
+1 Reduce a demonstrated effect

+1 Suggest a spurious effect</td></tr>
<tr><td>Moderate</td></tr>
<tr><td>Observational study</td><td>Low</td></tr>
</table>

		-1 Serious	when results show no effect
	Very Low	-2 Very serious<br><br>Publication Bias<br>-1 Likely<br>-2 Very likely	

For peer review only

For peer review only - http://bmjopen.bmj.com/site/about/guidelines.xhtml

Author	Publication year	Name of Data Extractor	Form: conference abstract only (1), journal publication (2), multiple publications of the same trial (3)	Study Design	Study Setting		Population	
					Country	Year(s) of Data Collection	Description of Participants	Inclusion Criteria

For peer review only

	% Female (N) (Give for total study population)
Exclusion Criteria	

For peer review only

	Macronutrient composition of RUTF in the intervention group		Iron dos/100g of RUTF in the experimental group	Zinc dos/100 mg of RUTF in the experimental group	Frequency of RUTF (packets per day)
Total number of eligible participants in all the study groups					

For peer review only

Duration of intervention RUTF (How many days of intervention was given)	Characteristic of comparison		Study confounders		What outcome did authors report	Notes
	Standard RTUFT? (Yes or No)	**Iron content in Standard RUTF (Normal dose is 10-14 mg/100 g)**	Were results adjusted for confounders? (Y/N) (Fill only for observational studies)	What confounders were considered?		

For peer review only

Study (name, year)	**Hemoglobin at longest follow up**		
	No of events in interventional RUTF	Total number in interventional RUTF	number of events in control RUTF group

For peer review only

	**Summary estimate of intervention RUTF vs. Standard RUTF (OR, RR, HR)**		Intervention
number of participants in control RUTF group	notes	notes	Mean

For peer review only

RUTF		Control RUTF			Intervention in RUTF	
SD	Number in F	Mean	SD	Number in t	median	IQR

For peer review only

	Control RUTF		
Number in g	Median	IQR	Number in g

For peer review only

Summary estimate (mean difference or Standardized mean difference)

ɟroup

For peer review only

Notes

For peer review only

	A	B	C	D	E	F	G	H	I	J	K	L	M	N	O	P
1		**Anemia at longest follow up**				**Summary estimate of intervention RUTF vs. Standard RUTF (OR, RR, HR)**		Intervention RUTF			Control RUTF			Intervention in RUTF		
2	Study (name, year)	No of events in interventional RUTF	RUTF	control RUTF group	RUTF group	notes	notes	Mean	SD	RUTF	Mean	SD	control RUTF	median	IQR	group

For peer review only

	Q	R	S	T
1	Control RUTF			Summary estimate (mean difference or Standardized mean difference)
2	Median	IQR	group	

For peer review only

Study (name, year)	Iron deficiancy anemia at longest follow up		
	No of events in interventional RUTF	Total number in interventional RUTF	number of events in control RUTF group

For peer review only

	**Summary estimate of intervention RUTF vs. Standard RUTF (OR, RR, HR)**		Intervention RUTF			Control RUT
number of participants in control RUTF group	notes	notes	Mean	SD	Number in RUTF interventional group	Mean

For peer review only

TF		Intervention in RUTF			Control RUTF		
SD	Number in the control RUTF group	median	IQR	Number in group	Median	IQR	Number in group

For peer review only

Summary estimate (mean difference or Standardized mean difference)

For peer review only

Study (name, year)	**SAM Recovery at longest follow up**				**Summary estimate of intervention RUTF vs. Standard RUTF (OR, RR, HR)**	
	No of events in interventional RUTF	Total number in interventional RUTF	number of events in control RUTF group	number of participants in control RUTF group	notes	notes

For peer review only

Study (name, year)	Adverse effects at longest follow up				Summary estimate of intervention RUTF vs. Standard RUTF (OR, RR, HR)
	No of events in interventional RUTF	Total number in interventional RUTF	number of events in control RUTF group	number of participants in control RUTF group	notes

For peer review only

notes

For peer review only

Study (name, year)	Adverse effects at longest follow up				Summary estimate of intervention RUTF vs. Standard RUTF (OR, RR, HR)	
	No of events in interventional RUTF	Total number in interventional RUTF	number of events in control RUTF group	number of participants in control RUTF group	notes	notes

For peer review only

	A	B	C	D	E	F	G	H	I	J	K	L	M	N
1	Study Name	Intervention RUTF			Control RUTF			Intervention in RUTF			Control RUTF			Summary esti
2		Mean	SD	Number in RUTF inter	Mean	SD	Number in the	median	IQR	Number in gro	Median	IQR	Number in group	
3														
4														

For peer review only

Study (name, year)	Adverse effects at longest follow up				Summary estimate of intervention RUTF vs. Standard RUTF (OR, RR, HR)	
	No of events in interventional RUTF	Total number in interventional RUTF	number of events in control RUTF group	number of participants in control RUTF group	notes	notes

For peer review only

	A	B	C	D	E	F	G	H	I	J	K	L	M	N	O	P	Q	R	S	T
1	Study ID (Last name and yea		Z scores						Kg						Change in weight (Kg) or Z scores					
2			Intervention			Control			Intervention			Control			Intervention			Control		
3			Mean	SD	Number in gro	Mean	SD	Number in gro	Mean	SD	Number in gro	Mean	SD	Number in gro	Mean	SD	Number in gro	Mean	SD	Number in gro
4																				
5																				

For peer review only

	U
1	Comments
2	
3	up
4	
5	

For peer review only

	A	B	C	D	E	F	G	H	I	J	K	L	M	N	O	P	Q	R	S	T
1	Study ID (Last name and yea		Z scores						Kg						Change in weight (Kg) or Z scores					
2			Intervention			Control			Intervention			Control			Intervention			Control		
3			Mean	SD	Number in gro	Mean	SD	Number in gro	Mean	SD	Number in gro	Mean	SD	Number in gro	Mean	SD	Number in gro	Mean	SD	Number in gro
4																				
5																				

For peer review only

	U
1	Comments
2	
3	up
4	
5	

For peer review only

	A	B	C	D	E	F	G	H	I	J	K	L	M	N	O	P	Q	R	S	T	U
1	Study ID (Last name and year)		Z scores						Centimeters						Change in height (cm) or Z scores						Comments
2			Intervention			Control			Intervention			Control			Intervention			Control			
3			Mean	SD	Number in gro	Mean	SD	Number in gro	Mean	SD	Number in gro	Mean	SD	Number in gro	Mean	SD	Number in gro	Mean	SD	Number in group	
4																					
5																					

For peer review only

	A	B	C	D	E	F	G	H	I	J	K	L	M	N	O	P	Q	R	S	T
1	Study ID (Last name and yea		Z scores						Centimeters						Change in height (cm) or Z scores					
2			Intervention			Control			Intervention			Control			Intervention			Control		
3			Mean	SD	Number in gro	Mean	SD	Number in gro	Mean	SD	Number in gro	Mean	SD	Number in gro	Mean	SD	Number in gro	Mean	SD	Number in gro
4																				
5																				

For peer review only

	U
1	Comments
2	
3	up
4	
5	

For peer review only

	A	B	C	D	E	F	G	H	I	J	K	L	M	N	O	P	Q	R	S	T
1	Study ID (Last name and year)		Z scores						Centimeters						Change in height (cm) or Z scores					
2			Intervention			Control			Intervention			Control			Intervention			Control		
3			Mean	SD	Number in gro	Mean	SD	Number in gro	Mean	SD	Number in gro	Mean	SD	Number in gro	Mean	SD	Number in gro	Mean	SD	Number in gro
4																				
5																				

For peer review only

	U
1	Comments
2	
3	up
4	
5	

For peer review only

	A	B	C	D	E	F	G	H	I	J	K	L	M	N	O	P	Q	R	S	T
1	Study ID (Last name and year)		Z scores						BMI						Change in BMI or Z scores					
2			Intervention			Control			Intervention			Control			Intervention			Control		
3			Mean	SD	Number in gro	Mean	SD	Number in gro	Mean	SD	Number in gro	Mean	SD	Number in gro	Mean	SD	Number in gro	Mean	SD	Number in gro
4																				
5																				

For peer review only

	U
1	Comments
2	
3	up
4	
5	

For peer review only

Study ID (Last name and year)	Z scores						BMI						Change in BMI or Z scores						Comments
	Intervention			Control			Intervention			Control			Intervention			Control			
	Mean	SD	Number in gro	Mean	SD	Number in gro	Mean	SD	Number in gro	Mean	SD	Number in gro	Mean	SD	Number in gro	Mean	SD	Number in group	

For peer review only

Study ID (Last name and year)	Z scores						BMI						Change in BMI or Z scores						Comments
	Intervention			Control			Intervention			Control			Intervention			Control			
	Mean	SD	Number in gro	Mean	SD	Number in gro	Mean	SD	Number in gro	Mean	SD	Number in gro	Mean	SD	Number in gro	Mean	SD	Number in group	

For peer review only

Appendix 1: Search strategies for different electronic databases

PubMed

("Iron"[MeSH Terms] OR "iron, dietary"[MeSH Terms] OR "Iron"[Title/Abstract] OR "56Fe"[Title/Abstract] OR "Fe"[Title/Abstract] OR "ferro"[Title/Abstract] OR "ferrum"[Title/Abstract]) AND ("food, formulated"[MeSH Terms] OR "food, fortified"[MeSH Terms] OR "RUTF"[Title/Abstract] OR "RUTFs"[Title/Abstract] OR "ready to use therapeutic food*"[Title/Abstract] OR "ready to use therapeutic feed*"[Title/Abstract] OR "therapeutic food*"[Title/Abstract] OR "therapeutic diet*"[Title/Abstract] OR "enriched food*"[Title/Abstract] OR "enriched diet*"[Title/Abstract] OR "fortified food*"[Title/Abstract] OR "fortified diet*"[Title/Abstract] OR "supplemented food*"[Title/Abstract] OR "supplemental food*"[Title/Abstract] OR "supplementary food*"[Title/Abstract] OR "supplement food*"[Title/Abstract] OR "supplemented diet*"[Title/Abstract] OR "supplemental diet*"[Title/Abstract] OR "supplementary diet*"[Title/Abstract] OR "supplement diet*"[Title/Abstract] OR "RUSF"[Title/Abstract] OR "RUSFs"[Title/Abstract] OR "ready to use supplemental food*"[Title/Abstract] OR "ready to use supplementary food*"[Title/Abstract] OR "lipid based nutrient supplement*"[Title/Abstract] OR "LNS"[Title/Abstract]) AND ("Severe Acute Malnutrition"[MeSH Terms] OR "Malnutrition"[MeSH Terms] OR "Cachexia"[MeSH Terms] OR "Wasting Syndrome"[MeSH Terms] OR "Severe Acute Malnutrition"[Title/Abstract] OR "SAM"[Title/Abstract] OR "Malnutrition"[Title/Abstract] OR "malnourish*"[Title/Abstract] OR "Cachexia"[Title/Abstract] OR "nutritional deficien*"[Title/Abstract] OR "undernutrition"[Title/Abstract] OR "deficient nutrition"[Title/Abstract] OR "undernourish*"[Title/Abstract] OR "wasting"[Title/Abstract] OR "wasted"[Title/Abstract]) AND ("Infant"[MeSH Terms] OR "Child"[MeSH Terms] OR "infant*"[Title/Abstract] OR "infancy"[Title/Abstract] OR "baby"[Title/Abstract] OR "babies"[Title/Abstract] OR "newborn*"[Title/Abstract] OR "neonat*"[Title/Abstract] OR "neo nat*"[Title/Abstract] OR "child*"[Title/Abstract] OR "toddler*"[Title/Abstract] OR "youth"[Title/Abstract] OR "juvenile*"[Title/Abstract] OR "girl*"[Title/Abstract] OR "boy"[Title/Abstract] OR "boys"[Title/Abstract] OR "preschool*"[Title/Abstract] OR "pre school*"[Title/Abstract])

CINAHL

S1 (MH "Infant+")
S2 (MH "Child+")
S3 TI (infant* OR infancy OR baby OR babies OR newborn* OR neonat* OR "neo nat*" OR child* OR toddler* OR youth OR juvenile* OR girl* OR boy OR boys OR preschool* OR "preschool*")
S4 AB (infant* OR infancy OR baby OR babies OR newborn* OR neonat* OR "neo nat*" OR child* OR toddler* OR youth OR juvenile* OR girl* OR boy OR boys OR preschool* OR "preschool*")
S5 S1 OR S2 OR S3 OR S4
S6 (MH "Iron")
S7 TI (iron OR 56Fe OR Fe Or ferro OR ferrum)
S8 AB (iron OR 56Fe OR Fe Or Ferro OR ferrum)
S9 S6 OR S7 OR S8
S10 (MH "Food, Formulated")
S11 (MH "Food, Fortified")
S12 TI (RUTF OR RUTFs OR "ready to use therapeutic food*" OR "ready to use therapeutic feed*" OR RUSF OR RUSFs OR "ready to use supplemental food*" OR "ready to use supplementary food*" OR "lipid-based nutrient supplement*" OR LNS)
S13 AB (RUTF OR RUTFs OR "ready to use therapeutic food*" OR "ready to use therapeutic feed*" OR RUSF OR RUSFs OR "ready to use supplemental food*" OR "ready to use supplementary food*" OR "lipid-based nutrient supplement*" OR LNS)
S14 TI((enriched OR fortified OR supplement* OR therapeutic) W1 (food* OR diet*))
S15 AB((enriched OR fortified OR supplement* OR therapeutic) W1 (food* OR diet*))
S16 S10 OR S11 OR S12 OR S13 OR S14 OR S15
S17 (MH "Cachexia")
S18 (MH "Malnutrition")
S19 (MH "Wasting Syndrome")

For peer review only - http://bmjopen.bmj.com/site/about/guidelines.xhtml

S20 TI ("Severe Acute Malnutrition" OR SAM OR Malnutrition OR malnourish* OR Cachexia OR undernutrition OR undernourish* OR wasting OR wasted)
S21 AB ("Severe Acute Malnutrition" OR SAM OR Malnutrition OR malnourish* OR Cachexia OR undernutrition OR undernourish* OR wasting OR wasted)
S22 TI (nutrition* N1 deficien*)
S23 AB (nutrition* N1 deficien*)
S24 S17 OR S18 OR S19 OR S20 OR S21 OR S22 OR S23
S25 S5 AND S9 AND S16 AND S24
S26 S25 Exclude MEDLINE records

Embase

#1 'iron'/exp OR 'iron intake'/exp
#2 iron:ti,ab OR 56fe:ti,ab OR fe:ti,ab OR ferro:ti,ab OR ferrum:ti,ab
#3 #1 OR #2
#4 'ready to use therapeutic food'/exp OR 'fortified food'/exp OR 'ready to use supplementary food'/exp OR 'lipid based nutrient supplement'/exp OR 'dietary supplement'/exp
#5 ((enriched OR fortified OR supplement* OR therapeutic) NEXT/1 (food* OR diet*)):ti,ab
#6 rutf:ti,ab OR rutfs:ti,ab OR 'ready to use therapeutic food*':ti,ab OR 'ready to use therapeutic feed*':ti,ab OR rusf:ti,ab OR rusfs:ti,ab OR 'ready to use supplemental food*':ti,ab OR 'ready to use supplementary food*':ti,ab OR 'lipid based nutrient supplement*':ti,ab OR lns:ti,ab
#7 #4 OR #5 OR #6
#8 'malnutrition'/exp OR 'wasting syndrome'/exp OR 'cachexia'/exp
#9 (nutrition* NEAR/1 deficien*):ti,ab
#10 'severe acute malnutrition':ti,ab OR sam:ti,ab OR malnutrition:ti,ab OR malnourish*:ti,ab OR cachexia:ti,ab OR undernutrition:ti,ab OR undernourish*:ti,ab OR wasting:ti,ab OR wasted:ti,ab
#11 #8 OR #9 OR #10
#12 'infant'/exp OR 'infancy'/exp OR 'baby'/exp OR 'newborn'/exp OR 'toddler'/exp OR 'child'/exp OR 'preschool child'/exp OR 'juvenile'/exp OR 'girl'/exp OR 'boy'/exp
#13 infant*:ti,ab OR infancy:ti,ab OR baby:ti,ab OR babies:ti,ab OR newborn*:ti,ab OR neonat*:ti,ab OR 'neo nat*':ti,ab OR child*:ti,ab OR toddler*:ti,ab OR youth:ti,ab OR juvenile*:ti,ab OR girl*:ti,ab OR boy:ti,ab OR boys:ti,ab OR preschool*:ti,ab OR 'pre school*':ti,ab
#14 #12 OR #13
#15 #3 AND #7 AND #11 AND #14
#16 #15 NOT [medline]/lim

SCOPUS

(TITLE-ABS (iron OR 56fe OR fe OR Ferro OR ferrum)) AND ((TITLE-ABS (enriched OR fortified OR supplement* OR therapeutic) PRE/1 TITLE-ABS (food* OR diet*)) OR (TITLE-ABS (rutf OR rutfs OR "ready to use therapeutic food*" OR "ready to use therapeutic feed*" OR rusf OR rusfs OR "ready to use supplemental food*" OR "ready to use supplementary food*" OR "lipid-based nutrient supplement*" OR lns))) AND ((TITLE-ABS (nutrition* W/1 deficien*)) OR (TITLE-ABS ("Severe Acute Malnutrition" OR sam OR malnutrition OR malnourish* OR cachexia OR undernutrition OR undernourish* OR wasting OR wasted))) AND (TITLE-ABS (infant* OR infancy OR baby OR babies OR newborn* OR neonat* OR neo-nat* OR child* OR toddler* OR youth OR juvenile* OR girl* OR boy OR boys OR preschool* OR pre-school*)) AND NOT INDEX (Medline)

CENTRAL

#1 MeSH descriptor: [Iron] in all MeSH products
#2 MeSH descriptor: [Iron, Dietary] explode all trees
#3 Iron:ti,ab OR 56Fe:ti,ab OR Fe:ti,ab OR ferro:ti,ab OR ferrum:ti,ab
#4 #1 OR #2 OR #3
#5 MeSH descriptor: [Food, Formulated] explode all trees
#6 MeSH descriptor: [Food, Fortified] explode all trees
#7 ((enriched OR fortified OR supplement* OR therapeutic) NEXT (food* OR diet*)):ti,ab

For peer review only - http://bmjopen.bmj.com/site/about/guidelines.xhtml

#8 (ready NEXT to NEXT use NEXT therapeutic NEXT food*):ti,ab
#9 (ready NEXT to NEXT use NEXT therapeutic NEXT feed*):ti,ab
#10 (ready NEXT to NEXT use NEXT supplemental NEXT food*):ti,ab
#11 (ready NEXT to NEXT use NEXT supplementary NEXT food*):ti,ab
#12 (lipid NEXT based NEXT nutrient NEXT supplement*):ti,ab
#13 rutf:ti,ab OR rutfs:ti,ab OR rusf:ti,ab OR rusfs:ti,ab OR lns:ti,ab
#14 #5 OR #6 OR #7 OR #8 OR #9 OR #10 OR #11 OR #12 OR #13
#15 MeSH descriptor: [Severe Acute Malnutrition] explode all trees
#16 MeSH descriptor: [Malnutrition] explode all trees
#17 MeSH descriptor: [Cachexia] explode all trees
#18 MeSH descriptor: [Wasting Syndrome] explode all trees
#19 (nutrition* NEAR/1 deficien*):ti,ab
#20 (severe NEXT acute NEXT malnutrition):ti,ab
#21 sam:ti,ab OR malnutrition:ti,ab OR malnourish*:ti,ab OR cachexia:ti,ab OR undernutrition:ti,ab OR undernourish*:ti,ab OR wasting:ti,ab OR wasted:ti,ab
#22 #15 OR #16 OR #17 OR #18 OR #19 OR #20 OR #21
#23 MeSH descriptor: [Infant] explode all trees
#24 MeSH descriptor: [Child] explode all trees
#25 infant*:ti,ab OR infancy:ti,ab OR baby:ti,ab OR babies:ti,ab OR newborn*:ti,ab OR neonat*:ti,ab OR neo NEXT nat*':ti,ab OR child*:ti,ab OR toddler*:ti,ab OR youth:ti,ab OR juvenile*:ti,ab OR girl*:ti,ab OR boy:ti,ab OR boys:ti,ab OR preschool*:ti,ab OR pre NEXT school*:ti,ab
#26 #23 OR #24 OR #25
#27 #4 AND #14 AND #22 AND #26
#28 "accession number" near pubmed
#29 #27 NOT #28

Web of Science

#1 TI=(iron OR 56fe OR fe OR ferro OR ferrum)
#2 AB=(iron OR 56fe OR fe OR ferro OR ferrum)
#3 #1 OR #2
#4 TI=(RUTF OR RUTFs OR "ready to use therapeutic food*" OR "ready to use therapeutic feed*" OR "therapeutic food*" OR "therapeutic diet*" OR "enriched food*" OR "enriched diet*" OR "fortified food*" OR "fortified diet*" OR "supplemented food*" OR "supplemental food*" OR "supplementary food*" OR "supplement food*" OR "supplemented diet*" OR "supplemental diet*" OR "supplementary diet*" OR "supplement diet*" OR RUSF OR RUSFs OR "ready to use supplemental food*" OR "ready to use supplementary food*" OR "lipid based nutrient supplement*" OR LNS)
#5 AB=(RUTF OR RUTFs OR "ready to use therapeutic food*" OR "ready to use therapeutic feed*" OR "therapeutic food*" OR "therapeutic diet*" OR "enriched food*" OR "enriched diet*" OR "fortified food*" OR "fortified diet*" OR "supplemented food*" OR "supplemental food*" OR "supplementary food*" OR "supplement food*" OR "supplemented diet*" OR "supplemental diet*" OR "supplementary diet*" OR "supplement diet*" OR RUSF OR RUSFs OR "ready to use supplemental food*" OR "ready to use supplementary food*" OR "lipid based nutrient supplement*" OR LNS)
#6 #4 OR #5
#7 TI=(nutrition* NEAR/1 deficien*)
#8 AB=(nutrition* NEAR/1 deficien*)
#9 TI=("Severe Acute Malnutrition" OR sam OR malnutrition OR malnourish* OR cachexia OR undernutrition OR undernourish* OR wasting OR wasted)
#10 AB=("Severe Acute Malnutrition" OR sam OR malnutrition OR malnourish* OR cachexia OR undernutrition OR undernourish* OR wasting OR wasted)
#11 #7 OR #8 OR #9 OR #10
#12 TI=(infant* OR infancy OR baby OR babies OR newborn* OR neonat* OR "neo nat*" OR child* OR toddler* OR youth OR juvenile* OR girl* OR boy OR boys OR preschool* OR "pre school*")

For peer review only - http://bmjopen.bmj.com/site/about/guidelines.xhtml

#13 AB=(infant* OR infancy OR baby OR babies OR newborn* OR neonat* OR "neo nat*" OR child* OR toddler* OR youth OR juvenile* OR girl* OR boy OR boys OR preschool* OR "pre school*")
#14 #12 OR #13
#15 #3 AND #6 AND #11 AND #14

LILACS

(mh:("Iron")) OR ((mh:("iron, dietary"))) OR ((ti:(iron OR 56fe OR fe OR ferro OR ferrum))) OR ((ab:(iron OR 56fe OR fe OR ferro OR ferrum))) AND ((mh:("food, formulated")) OR ((mh:("food, fortified"))) OR ((ti:(rutf OR rutfs OR "ready to use therapeutic food*" OR "ready to use therapeutic feed*" OR "therapeutic food*" OR "therapeutic diet*" OR "enriched food*" OR "enriched diet*" OR "fortified food*" OR "fortified diet*" OR "supplemented food*" OR "supplemental food*" OR "supplementary food*" OR "supplement food*" OR "supplemented diet*" OR "supplemental diet*" OR "supplementary diet*" OR "supplement diet*" OR rusf OR rusfs OR "ready to use supplemental food*" OR "ready to use supplementary food*" OR "lipid based nutrient supplement*" OR lns))) OR ((ab:(rutf OR rutfs OR "ready to use therapeutic food*" OR "ready to use therapeutic feed*" OR "therapeutic food*" OR "therapeutic diet*" OR "enriched food*" OR "enriched diet*" OR "fortified food*" OR "fortified diet*" OR "supplemented food*" OR "supplemental food*" OR "supplementary food*" OR "supplement food*" OR "supplemented diet*" OR "supplemental diet*" OR "supplementary diet*" OR "supplement diet*" OR rusf OR rusfs OR "ready to use supplemental food*" OR "ready to use supplementary food*" OR "lipid based nutrient supplement*" OR lns)))) AND ((mh:("Severe Acute Malnutrition")) OR ((mh:("Malnutrition"))) OR ((mh:("cachexia"))) OR ((mh:("wasting syndrome"))) OR ((ti:("Severe Acute Malnutrition" OR sam OR malnutrition OR malnourish* OR cachexia OR "nutritional deficien*" OR undernutrition OR "deficient nutrition" OR undernourish* OR wasting OR wasted))) OR ((ab:("Severe Acute Malnutrition" OR sam OR malnutrition OR malnourish* OR cachexia OR "nutritional deficien*" OR undernutrition OR "deficient nutrition" OR undernourish* OR wasting OR wasted)))) AND ((mh:(infant)) OR ((mh:(child))) OR ((ti:(infant* OR infancy OR baby OR babies OR newborn* OR neonat* OR "neo nat*" OR child* OR toddler* OR youth OR juvenile* OR girl* OR boy OR boys OR preschool* OR "pre school*"))) OR ((ab:(infant* OR infancy OR baby OR babies OR newborn* OR neonat* OR "neo nat*" OR child* OR toddler* OR youth OR juvenile* OR girl* OR boy OR boys OR preschool* OR "pre school*")))) AND (db:("LILACS"))

Global Index Medicus

((mh:("Iron")) OR ((mh:("iron, dietary"))) OR ((ti:(iron OR 56fe OR fe OR ferro OR ferrum))) OR ((ab:(iron OR 56fe OR fe OR ferro OR ferrum))) AND ((mh:("food, formulated")) OR ((mh:("food, fortified"))) OR ((ti:(rutf OR rutfs OR "ready to use therapeutic food*" OR "ready to use therapeutic feed*" OR "therapeutic food*" OR "therapeutic diet*" OR "enriched food*" OR "enriched diet*" OR "fortified food*" OR "fortified diet*" OR "supplemented food*" OR "supplemental food*" OR "supplementary food*" OR "supplement food*" OR "supplemented diet*" OR "supplemental diet*" OR "supplementary diet*" OR "supplement diet*" OR rusf OR rusfs OR "ready to use supplemental food*" OR "ready to use supplementary food*" OR "lipid based nutrient supplement*" OR lns))) OR ((ab:(rutf OR rutfs OR "ready to use therapeutic food*" OR "ready to use therapeutic feed*" OR "therapeutic food*" OR "therapeutic diet*" OR "enriched food*" OR "enriched diet*" OR "fortified food*" OR "fortified diet*" OR "supplemented food*" OR "supplemental food*" OR "supplementary food*" OR "supplement food*" OR "supplemented diet*" OR "supplemental diet*" OR "supplementary diet*" OR "supplement diet*" OR rusf OR rusfs OR "ready to use supplemental food*" OR "ready to use supplementary food*" OR "lipid based nutrient supplement*" OR lns)))) AND ((mh:("Severe Acute Malnutrition")) OR ((mh:("Malnutrition"))) OR ((mh:("cachexia"))) OR ((mh:("wasting syndrome"))) OR ((ti:("Severe Acute Malnutrition" OR sam OR malnutrition OR malnourish* OR cachexia OR "nutritional deficien*" OR undernutrition OR "deficient nutrition" OR undernourish* OR wasting OR wasted))) OR ((ab:("Severe Acute Malnutrition" OR sam OR malnutrition OR malnourish* OR cachexia OR "nutritional deficien*" OR undernutrition OR "deficient nutrition" OR undernourish* OR wasting OR wasted)))) AND ((mh:(infant)) OR ((mh:(child))) OR ((ti:(infant* OR infancy OR baby OR babies OR newborn* OR neonat* OR "neo nat*" OR child* OR toddler* OR youth OR juvenile* OR girl* OR boy OR boys OR preschool* OR "pre school*"))) OR ((ab:(infant* OR infancy OR baby OR babies OR newborn* OR neonat* OR "neo nat*" OR child* OR toddler* OR youth OR juvenile* OR girl* OR boy OR boys OR preschool* OR "pre school*"))))) AND (collection_gim:("IMSEAR" OR "IMEMR" OR "WPRIM"))

For peer review only - http://bmjopen.bmj.com/site/about/guidelines.xhtml

Routine antibiotics for infants less than six months of age with growth failure/faltering. A protocol for the systematic review

Aamer Imdad, Fanny F Chen, Melissa Francois, Emily Tanner-Smith, Abigail Smith, Olivia J Tsistinas, Jai Das, Zulfiqar Ahmed Bhutta

PRISMA-P (Preferred Reporting Items for Systematic review and Meta-Analysis Protocols) 2015 checklist: recommended items to address in a systematic review protocol*

Section and topic	Item No	Checklist item	This manuscript
**ADMINISTRATIVE INFORMATION**			
Title:			
Identification	1a	Identify the report as a protocol of a systematic review	Page 1
Update	1b	If the protocol is for an update of a previous systematic review, identify as such	
Registration	2	If registered, provide the name of the registry (such as PROSPERO) and registration number	Application pending
Authors:			
Contact	3a	Provide name, institutional affiliation, e-mail address of all protocol authors; provide physical mailing address of corresponding author	Page 1
Contributions	3b	Describe contributions of protocol authors and identify the guarantor of the review	Page 11
Amendments	4	If the protocol represents an amendment of a previously completed or published protocol, identify as such and list changes; otherwise, state plan for documenting important protocol amendments	Not Applicable
Support:			
Sources	5a	Indicate sources of financial or other support for the review	Page 11
Sponsor	5b	Provide name for the review funder and/or sponsor	Page 11
Role of sponsor or funder	5c	Describe roles of funder(s), sponsor(s), and/or institution(s), if any, in developing the protocol	Page 11
**INTRODUCTION**			
Rationale	6	Describe the rationale for the review in the context of what is already known	Page 5
Objectives	7	Provide an explicit statement of the question(s) the review will address with reference to participants, interventions, comparators, and outcomes (PICO)	Page 6 and 7
**METHODS**			

For peer review only

Eligibility criteria	8	Specify the study characteristics (such as PICO, study design, setting, time frame) and report characteristics (such as years considered, language, publication status) to be used as criteria for eligibility for the review	Page 6
Information sources	9	Describe all intended information sources (such as electronic databases, contact with study authors, trial registers or other grey literature sources) with planned dates of coverage	Page 7
Search strategy	10	Present draft of search strategy to be used for at least one electronic database, including planned limits, such that it could be repeated	Appendix 1
Study records:			
Data management	11a	Describe the mechanism(s) that will be used to manage records and data throughout the review	Page 8
Selection process	11b	State the process that will be used for selecting studies (such as two independent reviewers) through each phase of the review (that is, screening, eligibility and inclusion in meta-analysis)	Page 8
Data collection process	11c	Describe planned method of extracting data from reports (such as piloting forms, done independently, in duplicate), any processes for obtaining and confirming data from investigators	Page 8
Data items	12	List and define all variables for which data will be sought (such as PICO items, funding sources), any pre-planned data assumptions and simplifications	Page 9
Outcomes and prioritization	13	List and define all outcomes for which data will be sought, including prioritization of main and additional outcomes, with rationale	Page 6 and 7
Risk of bias in individual studies	14	Describe anticipated methods for assessing risk of bias of individual studies, including whether this will be done at the outcome or study level, or both; state how this information will be used in data synthesis	Page 9
Data synthesis	15a	Describe criteria under which study data will be quantitatively synthesised	Page 9
	15b	If data are appropriate for quantitative synthesis, describe planned summary measures, methods of handling data and methods of combining data from studies, including any planned exploration of consistency (such as I^2, Kendall's τ)	Page 9
	15c	Describe any proposed additional analyses (such as sensitivity or subgroup analyses, meta-regression)	Page 10
	15d	If quantitative synthesis is not appropriate, describe the type of summary planned	Page 10
Meta-bias(es)	16	Specify any planned assessment of meta-bias(es) (such as publication bias across studies, selective reporting within studies)	Page 10
Confidence in cumulative evidence	17	Describe how the strength of the body of evidence will be assessed (such as GRADE)	Page 9 and 10

For peer review only
